# Efficacy and safety of acupuncture therapy for chronic spontaneous urticaria: a network meta-analysis based on randomized controlled trials

**DOI:** 10.3389/fmed.2026.1875737

**Published:** 2026-09-30

**Authors:** Mingde Chang, Linna Wu, Zhao Wang, Yurou Wang, Shuihan Zhou, Rong Zhao, Mingling Chen

**Affiliations:** 1 Clinical Medical College, Chengdu University of Traditional Chinese Medicine, Chengdu, Sichuan, China; 2 Department of Dermatology, Hospital of Chengdu University of Traditional Chinese Medicine, Chengdu, Sichuan, China; 3 The First Clinical Medical School, Yunnan University of Chinese Medicine, Kunming, Yunnan, China; 4 National Clinical Research Center for Chinese Medicine Acupuncture and Moxibustion, First Teaching Hospital of Tianjin University of Traditional Chinese Medicine, Tianjin, China; 5 Jiangxi University of Chinese Medicine, Jiangxi, Nanchang, China

**Keywords:** acupuncture therapy, chronic spontaneous urticaria, efficacy, network meta-analysis, systematic review

## Abstract

**Background:**

Chronic spontaneous urticaria (CSU) is a common refractory skin disorder impairing quality of life. This study systematically evaluated the clinical efficacy and safety of acupuncture therapies for CSU, providing evidence for clinical decision-making.

**Methods:**

Eight databases—CNKI, VIP, WanFang, SinoMed, PubMed, Embase, Cochrane Library, and Web of Science—were searched from their inception to 31 July 2025, for randomized controlled trials (RCTs) investigating various acupuncture therapies for CSU. The quality of the included studies was assessed using the Risk of Bias 2 tool. Data analysis was performed using R4.4.2 and Stata 18. This study was registered in PROSPERO (https://www.crd.york.ac.uk/PROSPERO/view/CRD42024608056).

**Results:**

Ninety-three RCTs involving 7,746 patients were included. Based on surface under the cumulative ranking curve (SUCRA) analysis, acupuncture combined with autohemotherapy ranked first in improving efficacy [SUCRA = 92.79%, relative risk (RR) = 1.46, 95% credible interval (CrI): 1.19, 1.84]. Moxibustion ranked first in reducing the Dermatology Life Quality Index (DLQI) score [SUCRA = 99.94%, mean difference (MD) = −9.45, 95% CrI: −12.48, −6.44]. In terms of wheal count, most pairwise comparisons among interventions showed no significant differences, and none of the acupuncture therapies demonstrated statistical superiority over antihistamines. Acupuncture combined with moxibustion ranked first in reducing the severity of pruritus (SUCRA = 96.27%, MD = −1.03, 95% CrI: −1.15, −0.91). Autohemotherapy combined with moxibustion ranked first in reducing the levels of immunoglobulin E (IgE) (SUCRA = 93.05%, MD = −36.23, 95% CrI: −55.33, −17.33). The combination of acupuncture, bloodletting puncture and cupping (BLP-C), and antihistamine ranked first in both reducing interleukin-4 (SUCRA = 97.95%, MD = −194.39, 95% CrI: −317.68, −71.10) and increasing IFN-γ (SUCRA = 99.78%, MD = 32.36, 95% CrI: 19.00, 46.49). Acupuncture combined with cupping therapy ranked first in reducing the incidence of adverse reactions (SUCRA = 91.69%, RR = 0.04, 95% CrI: 0.04, 0.24) and relapse rate (SUCRA = 91.79%, RR = 0.02, 95% CrI: 0, 0.09).

**Conclusion:**

Acupuncture therapies demonstrate good efficacy and safety in treating CSU. Nevertheless, optimal interventions should be selected based on the characteristics of symptoms, immune profiles, and patient tolerability. These findings should be interpreted cautiously and validated in larger, higher-quality trials due to study limitations.

**Systematic Review Registration:**

identifier CRD42024608056.

## 1 Introduction

Chronic spontaneous urticaria (CSU) is a localized edematous reaction resulting from dilation and increased permeability of cutaneous and mucosal microvessels. It is clinically characterized by recurrent wheals that occur daily or intermittently, with the condition persisting for at least 6 weeks. CSU has high incidence rates, prolonged disease course, complex pathogenesis, and is difficult to cure naturally (1). The point prevalence of CSU is 1.4% in Asia, 0.5% in Europe, and 0.1% in North America. The global prevalence is approximately 0.7% (2). Recurrent outbreaks of CSU serve as a major source of psychological distress, contributing to irritation, anxiety, depression, and impaired social functioning in patients (3, 4). Individuals with CSU exhibit long-term consumption of healthcare resources (5), imposing long-term pressure on public health systems and society.

Second-generation H1-antihistamines are widely employed as first-line therapy for CSU due to their rapid and sustained antihistaminic effects (6, 7). However, a substantial proportion of patients fail to achieve symptom remission with standard-dosage regimens (8–11). As a second-line treatment for CSU (7), Omalizumab has demonstrated efficacy in reducing wheal counts and alleviating pruritus (12–14). Nevertheless, its high cost imposes a significant financial burden on patients (15). For refractory cases, the third-line agent cyclosporine may be employed. Cyclosporine is primarily used for rapid control of acute exacerbations (16) and can relieve symptoms in most patients who are resistant to antihistamines. However, it has significant adverse effects (17), including hypertension, gastrointestinal damage, hepatotoxicity, nephrotoxicity, and even skin cancer (18). As none of these therapies provide complete control of CSU symptoms, additional effective and safe treatment options are required (7).

Acupuncture therapy ranks among the most widely used complementary and alternative therapies worldwide (19). Acupuncture activates immune cells and enhances immunity by stimulating specific cutaneous acupoints. It has been extensively applied in the management of various diseases (20–22) and is increasingly used in the treatment of CSU. Acupuncture therapies include acupuncture, moxibustion, acupoint catgut embedding (ACE), electroacupuncture, autohemotherapy, bloodletting (BLT), and point application. Current evidence indicates that these techniques are clinically effective in alleviating the symptoms of CSU (23–25), although the results of these studies are inconsistent.

To better evaluate the effectiveness and safety of acupuncture in treating CSU, this study sought to conduct a network meta-analysis (NMA) of published randomized controlled trials (RCTs) to integrate both direct and indirect comparative evidence, constructing a network structure, estimating the efficacy of interventions, and ranking their advantages (26). This approach is anticipated to provide robust evidence that may guide evidence-based clinical decision-making for practitioners and patients. Moreover, this study compared the effects of acupuncture therapies with other conventional treatments and identified the differences between various acupuncture treatments.

## 2 Methods

This study followed the Preferred Reporting Items for Systematic Reviews and Meta-Analyses (PRISMA) guidelines and its requirements for NMA. The study protocol was registered in the international prospective systematic review registry (PROSPERO CRD42024608056).

### 2.1 Search strategy

Eight databases—CNKI, VIP, WanFang, SinoMed, PubMed, Embase, Cochrane Library, and Web of Science—were searched from their inception to 31 July 2025 (Initial search: 1 September 2024; updated search: 31 July 2025). The search strategy was designed using a combination of medical subject headings (MeSH) and free terms. The MeSH used were as follows: (antihistamine + warm-needle moxibustion + electroacupuncture + moxibustion + auricular point sticking + ACE + acupoint injection therapy + fire needle + head needle + point application + intradermal needle + microneedle + navel moxibustion + transcutaneous electrical nerve stimulation + body acupuncture + scalp acupuncture + laser acupuncture on the wrist and ankle) and (CSU). Moreover, we manually searched the reference lists of related articles and gray literature to identify eligible studies. The specific search strategy is provided in Supplementary Table S1.

### 2.2 Outcome measures

The primary outcome measures included efficacy and the Dermatology Life Quality Index (DLQI) score. This study adopted the Symptom Score Reduction Index (SSRI) as the key indicator for assessing efficacy, classifying treatment outcomes into four categories: cured (SSRI ≥ 90%), markedly effective (60% ≤ SSRI < 90%), effective (20% ≤ SSRI < 60%), and ineffective (SSRI < 20%). The determination of “cured” and “markedly effective” was based on objective improvement in major clinical signs, primarily the resolution of wheals and relief from pruritus. These criteria were further confirmed by long-term follow-up data on recurrence. The specific efficacy criteria used in each included study are provided in Supplementary Table S2. The DLQI score is a patient self-administered questionnaire comprising 10 items, each scored on a 4-point scale (0–3 points). It is designed to systematically assess the burden on quality of life (QoL) in adult patients across six dimensions, including skin symptoms, daily activities, and interpersonal relationships. The total score ranges from 0 to 30, with higher scores indicating more severe impairment of QoL.

Secondary outcome measures included wheal counts, the severity of pruritus, immunoglobulin E (IgE), interleukin-4 (IL-4), IFN-γ, adverse reactions, and relapse rate. Wheal counts refer to the total number of newly developed wheals in CSU patients within 24 h, quantified on a 0–3 point scale (0 = none, 1 = <20, 2 = 20–50, 3 = >50/confluent). It is a core component of the Urticaria Activity Score (UAS7). The severity of pruritus refers to the patient's self-assessment of pruritus sensation during the preceding 24 h according to UAS7. It is graded on a 0–3 point scale: 0 = no pruritus, 1 = mild (perceptible but not bothersome), 2 = moderate (noticeable and bothersome, but not interfering with daily activities or sleep), and 3 = severe (intense pruritus, significantly interfering with daily activities and/or sleep). The scoring scales used are presented in Supplementary Table S3.

### 2.3 Inclusion and exclusion criteria

Inclusion criteria were as follows: (i) Study population included patients with CSU. All included patients presented with wheals, angioedema, or both, with symptoms occurring daily or intermittently. The disease duration exceeded 6 weeks since initial onset. (ii) Intervention and exposure included acupuncture therapies (encompassing acupuncture, moxibustion, cupping therapy, autohemotherapy, point application, ACE, and fire needle) alone or in combination with other therapies. The specific procedures for each intervention are provided in Supplementary Table S2. (iii) Study design was RCTs. (iv) Outcome measures included efficacy, the DLQI score, wheal count, severity of pruritus, IgE, IL-4, IFN-γ, adverse reactions, and relapse rate.

Exclusion criteria were as follows: (i) animal or cell experiments, case reports, scientific research plans, reviews, letters, editorials, and conference abstracts; (ii) studies with missing or erroneous data; (iii) duplicate publications; (iv) studies for which full texts were not attainable; and (v) studies with overlapping participants.

### 2.4 Data extraction

The retrieved studies were imported into EndNote. Two researchers (CMD and WLN) independently screened the titles and abstracts based on the inclusion and exclusion criteria. Full texts of the studies were reviewed to determine final eligibility. Any disagreements were resolved through discussions or by consulting a third researcher (CML). Data extraction was independently performed by the two researchers using a predesigned electronic form. The extracted data included the first author, year of publication, country, randomization and blinding methods, intervention and control measures, duration of treatment, basic features of study participants, and outcome measures.

### 2.5 Quality assessment

Two authors (CMD and WLN) independently assessed the quality of eligible studies using the Cochrane Risk-of-Bias tool (RoB 2). This tool includes five domains: the generation of random sequence, allocation concealment, blinding implementation, data missingness, and selective reporting. Every domain was rated as high risk of bias, some concerns, or low risk of bias based on the levels of risks. In the overall quality assessment, if all five domains were rated as low risk of bias or if only one domain was rated as some concerns and the remaining domains as low risk of bias, that study was rated as low risk of bias. If four or more domains were rated as having some concerns or if any domain was rated as high risk of bias, that study was rated as high risk of bias. The remaining cases were classified as some concerns. The two authors independently conducted the quality assessment. Any disagreements were resolved through discussions or by consulting a third researcher (CML).

### 2.6 Statistical analysis

The outcome measures of the included studies were continuous and binary variables. Weighted mean difference and risk ratio (RR), along with their corresponding 95% credible intervals (CrI), were used as the metric of the effect size. This study employed the Markov chain Monte Carlo (MCMC) method to construct a Bayesian-based NMA model. The model was iterated to assess the relative effectiveness of different treatment options. During the testing process, the model chain value was set to 4, the annealing value to 10,000, and the iteration value to 50,000, with a step size of 10 and an initial value of 2.5. This process aimed to obtain posterior distributions. The implementation of the NMA should meet the three fundamental assumptions of transitivity, homogeneity, and consistency. The heterogeneity between studies was assessed using the mtc:anohe function in the GeMTC package. When the overall *I*^2^ < 50%, the heterogeneity among included studies within the same comparison was considered acceptable, indicating that the homogeneity assumption was met. The mtc.nodesplit function from the GeMTC package was employed to evaluate inconsistency between direct and indirect comparisons through the node-splitting method. *P* > 0.05 indicated that no inconsistency existed between direct and indirect comparisons, indicating that the consistency assumption was met. Convergence of the basic characteristics of the patients and study outcomes was assessed using the Gelman–Rubin diagnostic. A potential scale reduction factor value of 1 was used as the benchmark. The relationships between the standardized mean differences of various interventions were represented by lines, with interventions as nodes, collectively forming the network structure. The cumulative ranking probability plot was analyzed to assess all cumulative ranking probabilities, which were then reported as the surface under the cumulative ranking curve (SUCRA). This network structure used nodes to represent varied interventions. It also used connecting lines to represent head-to-head comparisons between interventions. The size of the nodes and the thickness of the connecting lines correspond to the total number of trials included for each intervention and the number of comparative studies between interventions, respectively. Funnel plots were employed to evaluate potential publication bias. All statistical analyses were performed using R (version 4.4.2) and STATA (version 18).

## 3 Results

### 3.1 Literature search and screening

In total, 3,231 articles were retrieved. After removing 2,063 duplicates, we screened the titles and abstracts of the remaining records and excluded 978 articles. The full texts of the remaining 190 studies were assessed for eligibility based on predefined criteria. Finally, 93 studies were included. The screening process is presented in Figure 1.

**Figure 1:**
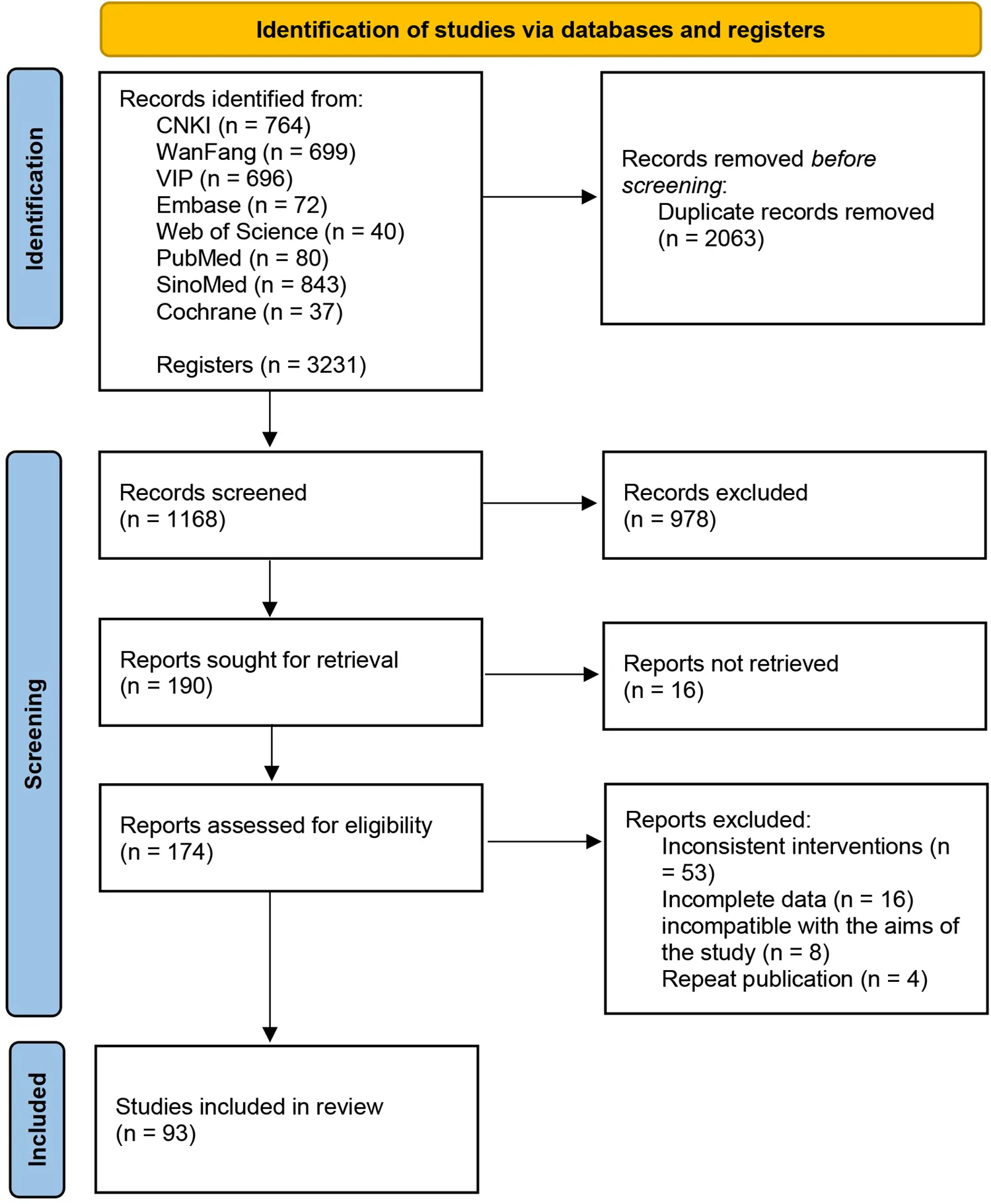
Flowchart of literature search and selection.

### 3.2 Basic characteristics and quality assessment of included studies

The 93 included studies (27–119) were all from China, involving 7,746 patients. Among them, there were 3,663 patients in the intervention group and 4,083 in the control group. The ages ranged from 13 to 71 years. The basic characteristics of the included studies are presented in Supplementary Table S4.

The majority of the included studies demonstrated a low risk of bias. Three studies (30, 31, 66) were rated as having some concerns, mainly due to the failure to report randomization methods. Among the included studies, 4 studies (43, 63, 73, 79) used random allocation through lottery, 39 studies (29, 34, 40, 48, 49, 53, 60, 69, 72, 75–78, 82, 83, 85, 88–91, 97, 99–101, 104, 105, 113–116, 118, 120) used a random number table to generate random sequences, 1 study (108) employed a combination of random number and envelope methods, 1 study (112) employed a simple numerical randomization method, and 38 studies (27, 28, 30–33, 35, 38, 39, 41, 44–46, 51, 52, 54, 55, 57, 62, 64–68, 70, 74, 80, 81, 87, 93, 95, 96, 98, 102, 110, 117, 121) mentioned randomization but did not specify the method used. Six studies (42, 48, 58, 61, 84, 92) used the order of patient visits for randomization and four studies (50, 71, 103, 111) did not mention randomization methods. Regarding allocation concealment and blinding, the blinding of practitioners was virtually impossible due to the nature of the interventions. For incomplete outcome data, 92 studies reported no attrition and provided complete datasets. One study (109) documented one withdrawal within the experimental group and three within the control group. Specific results are illustrated in Figure 2.

**Figure 2:**
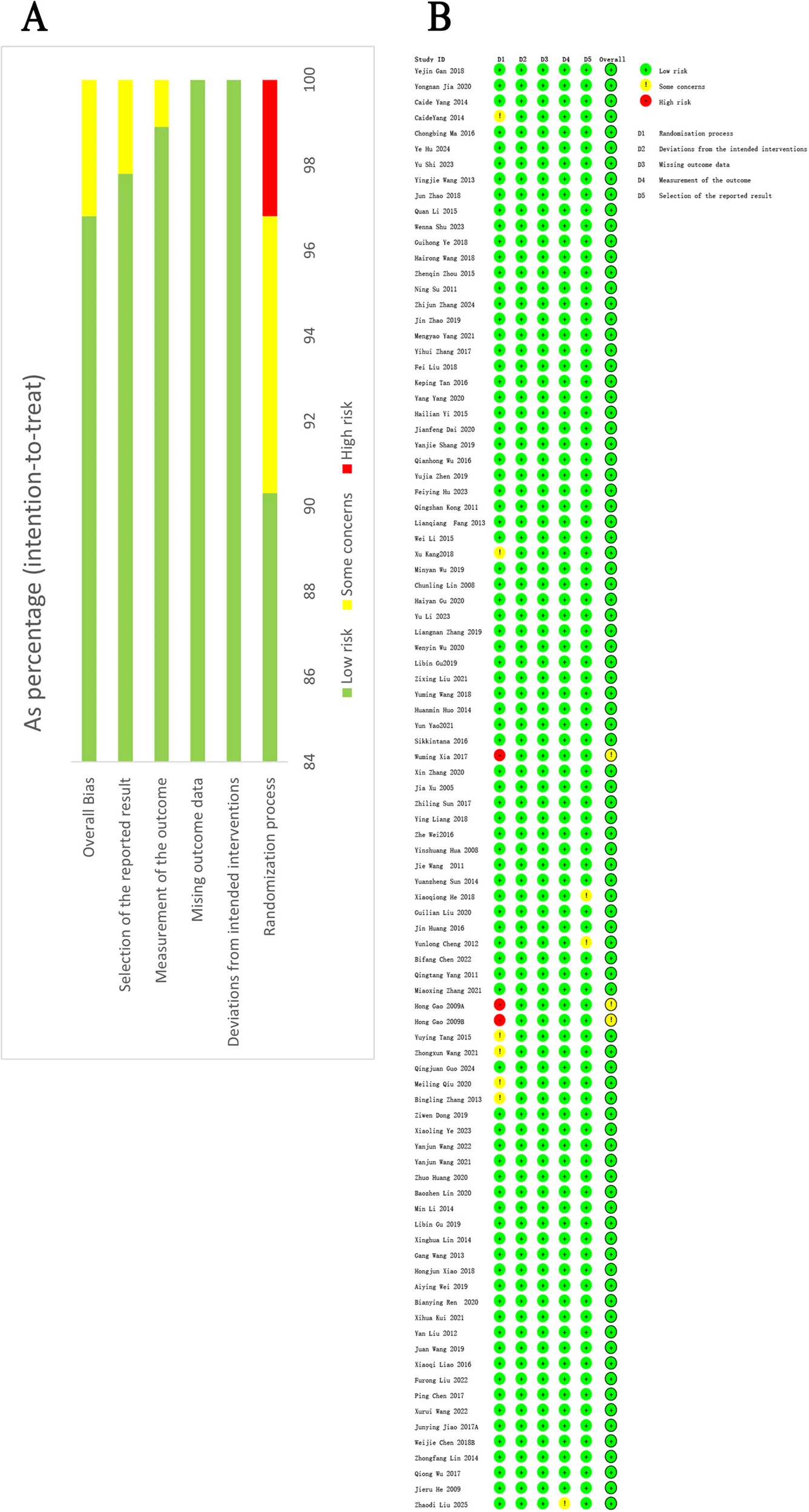
Quality assessment results. **(A)** Risk of bias plot; **(B)** summary of risk of bias.

### 3.3 Results of NMA

#### 3.3.1 Network diagram

In the network diagram, every node represented an intervention. The size of the node was positively associated with the number of studies involving that intervention. Larger nodes represented a greater number of included studies. The lines connecting two nodes represented direct comparative studies between interventions. The thickness of the line represented the number of studies comparing the two interventions, with thicker lines indicating more studies (Figure 3). Node-splitting analysis was conducted to assess the results of every closed loop (Figure 4). Significant local inconsistency was observed for efficacy (*P* < 0.05), whereas no local inconsistency was found for the other outcome indicators (all *P* > 0.05).

**Figure 3:**
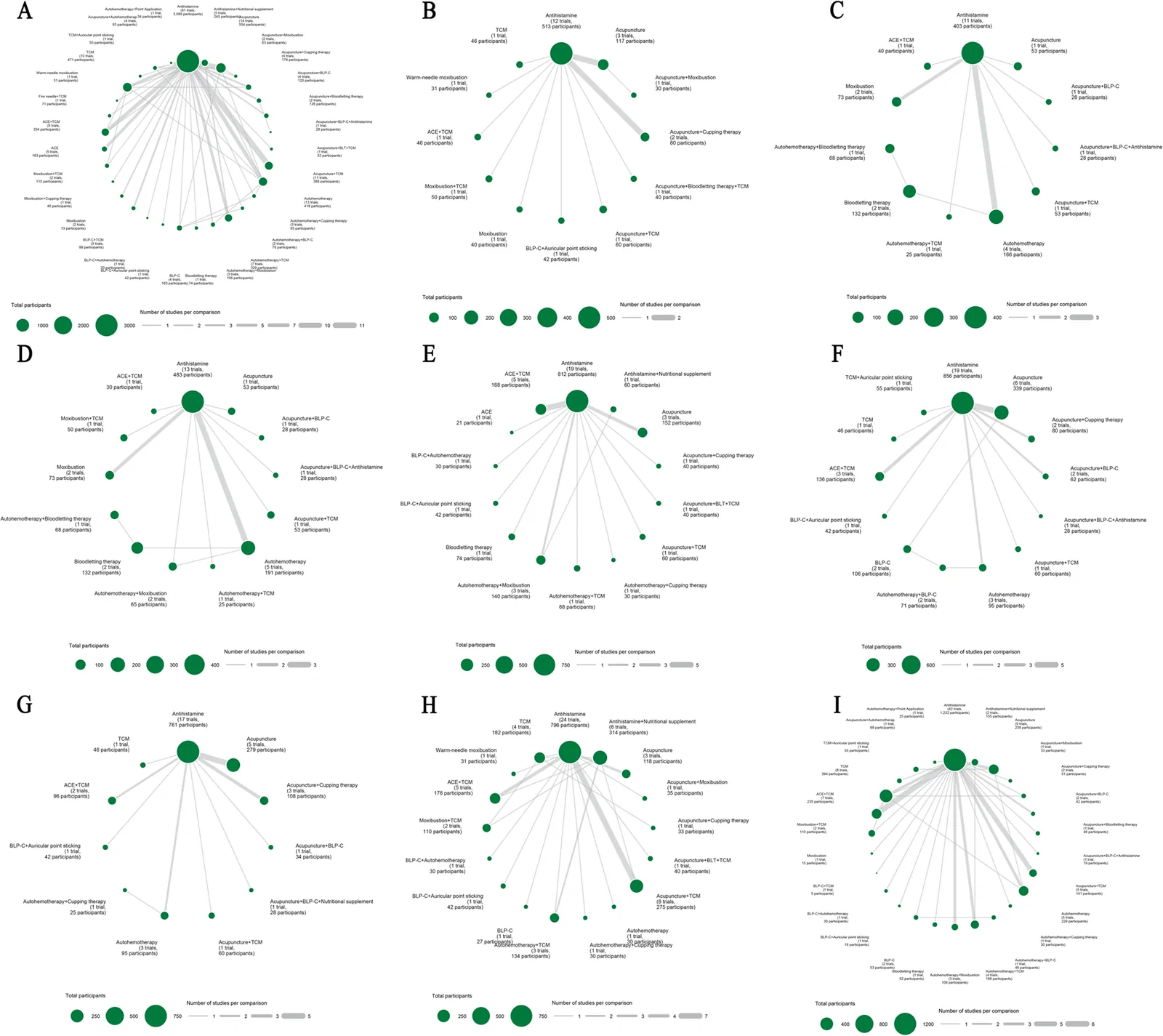
Network plot. **(A)** Efficacy; **(B)** DLQI score; **(C)** wheal count; **(D)** severity of pruritus; **(E)** IgE; **(F)** IL-4; **(G)** IFN-γ; **(H)** adverse reactions; **(I)**: relapse rate.

**Figure 4:**
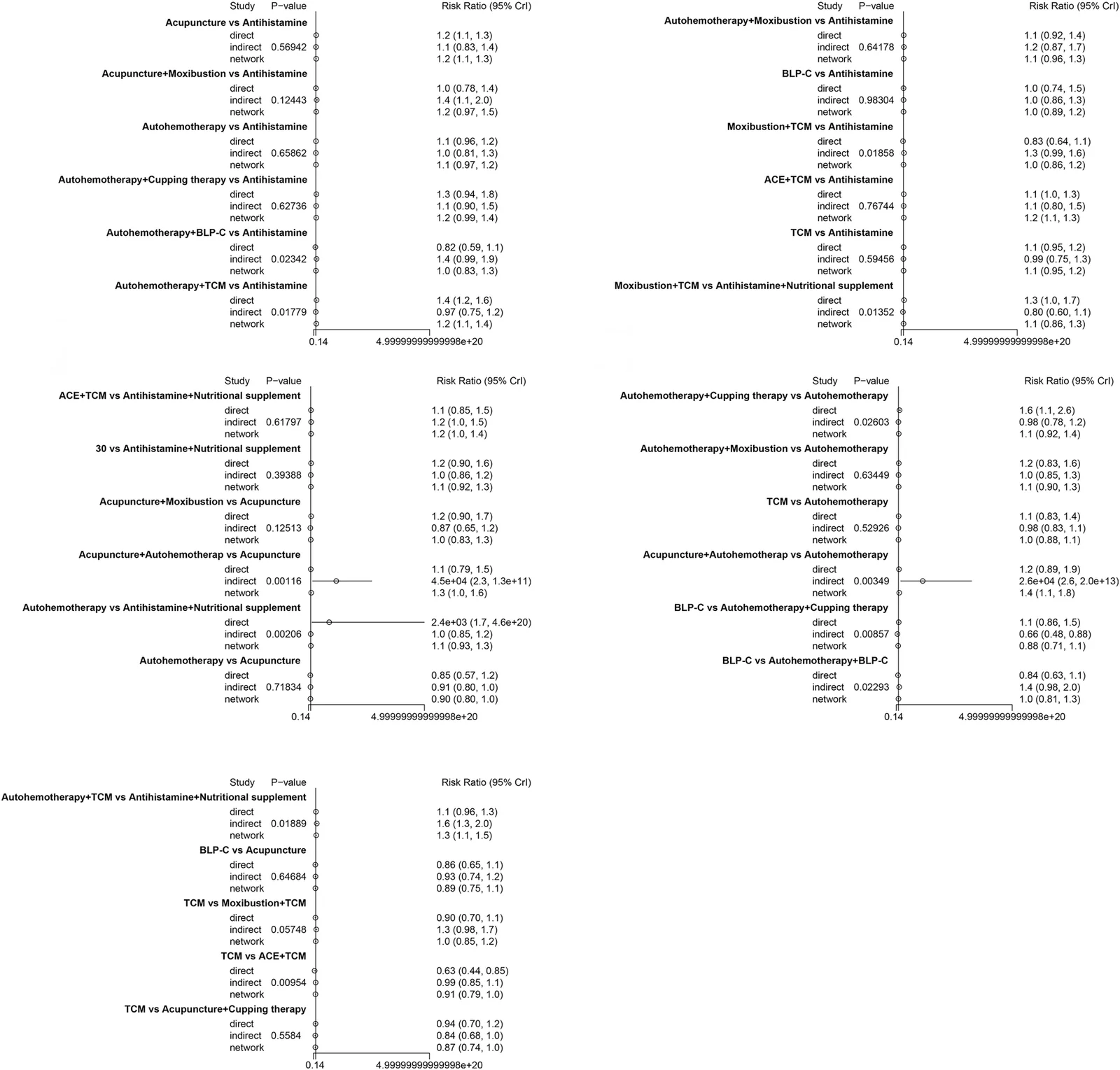
Node-splitting analysis of treatment efficacy, with *P* < 0.05 signifying local inconsistency.

#### 3.3.2 Efficacy

In total, 89 studies reported efficacy outcomes. The NMA results showed that, for patients with CSU, acupuncture combined with autohemotherapy was more effective than antihistamine alone [acupuncture combined with autohemotherapy vs. antihistamine: RR = 1.46, 95% CrI = (1.19, 1.84)] (Table 1).

**Table 1:** Pairwise comparison results of varied interventions on efficacy.

Antihistamine																														
0.98 (0.85, 1.12)	Antihistamine + nutritional supplements																													
**1.17** (**1.07, 1.28)**	**1.2** (**1.02, 1.42)**	Acupuncture																												
1.2 (0.97, 1.49)	1.23 (0.95, 1.59)	1.02 (0.82, 1.28)	Acupuncture + moxibustion																											
**1.21** (**1.05, 1.39)**	**1.24** (**1.02, 1.51)**	1.03 (0.87, 1.22)	1.01 (0.78, 1.3)	Acupuncture + cupping therapy																										
1.17 (0.97, 1.42)	1.2 (0.95, 1.52)	1 (0.81, 1.24)	0.98 (0.73, 1.3)	0.97 (0.77, 1.23)	Acupuncture + BLP-C																									
1.04 (0.88, 1.24)	1.07 (0.86, 1.33)	0.89 (0.73, 1.08)	0.87 (0.66, 1.15)	0.86 (0.69, 1.08)	0.89 (0.69, 1.15)	Acupuncture + bloodletting therapy																								
1.12 (0.82, 1.51)	1.15 (0.82, 1.6)	0.96 (0.69, 1.31)	0.94 (0.64, 1.35)	0.93 (0.66, 1.29)	0.96 (0.71, 1.27)	1.08 (0.75, 1.52)	Acupuncture + BLP-C + western medicine																							
1.06 (0.82, 1.39)	1.09 (0.81, 1.47)	0.91 (0.69, 1.2)	0.89 (0.63, 1.25)	0.88 (0.66, 1.19)	0.91 (0.66, 1.26)	1.02 (0.75, 1.4)	0.95 (0.64, 1.42)	Acupuncture + BLT + TCM																						
**1.28** (**1.16, 1.41)**	**1.31** (**1.11, 1.56)**	1.09 (0.96, 1.25)	1.07 (0.84, 1.35)	1.06 (0.89, 1.26)	1.09 (0.88, 1.35)	**1.22** (**1.01, 1.5)**	1.14 (0.83, 1.58)	1.2 (0.91, 1.58)	Acupuncture + TCM																					
1.06 (0.97, 1.15)	1.08 (0.93, 1.27)	0.9 (0.8, 1.02)	0.88 (0.7, 1.11)	0.87 (0.74, 1.03)	0.9 (0.73, 1.11)	1.01 (0.84, 1.23)	0.94 (0.69, 1.31)	0.99 (0.75, 1.31)	0.83 (0.72, 0.94)	Autohemotherapy																				
1.19 (0.99, 1.45)	1.22 (0.97, 1.55)	1.02 (0.83, 1.25)	0.99 (0.75, 1.32)	0.98 (0.78, 1.25)	1.02 (0.78, 1.33)	1.14 (0.89, 1.48)	1.06 (0.75, 1.53)	1.12 (0.81, 1.55)	0.93 (0.75, 1.16)	1.13 (0.93, 1.38)	Autohemotherapy + cupping therapy																			
1.04 (0.83, 1.3)	1.07 (0.82, 1.39)	0.89 (0.7, 1.12)	0.87 (0.64, 1.18)	0.86 (0.66, 1.12)	0.89 (0.66, 1.19)	1 (0.75, 1.33)	0.93 (0.64, 1.36)	0.98 (0.69, 1.38)	0.82 (0.64, 1.04)	0.99 (0.78, 1.25)	0.88 (0.66, 1.15)	Autohemotherapy + BLP-C																		
1.23 (1.08, 1.41)	1.26 (1.1, 1.46)	1.05 (0.9, 1.24)	1.03 (0.8, 1.32)	1.02 (0.85, 1.24)	1.05 (0.84, 1.32)	1.18 (0.96, 1.47)	1.09 (0.79, 1.54)	1.15 (0.87, 1.55)	0.96 (0.82, 1.14)	1.16 (1, 1.36)	1.03 (0.82, 1.3)	1.18 (0.91, 1.54)	Autohemotherapy + TCM	1.08 (0.88, 1.34)																
1.14 (0.97, 1.35)	1.16 (0.94, 1.45)	0.97 (0.81, 1.18)	0.95 (0.72, 1.25)	0.94 (0.76, 1.17)	0.97 (0.75, 1.25)	1.09 (0.86, 1.39)	1.01 (0.72, 1.45)	1.07 (0.78, 1.46)	0.89 (0.74, 1.08)	1.08 (0.9, 1.29)	0.96 (0.74, 1.22)	1.09 (0.83, 1.45)	0.92 (0.75, 1.14)	Autohemotherapy + moxibustion																
**1.41** (**1.08, 1.89)**	**1.45** (**1.07, 2)**	1.21 (0.91, 1.63)	1.18 (0.83, 1.69)	1.17 (0.86, 1.61)	1.21 (0.87, 1.7)	1.36 (0.98, 1.9)	1.26 (0.84, 1.92)	1.33 (0.91, 1.96)	1.11 (0.83, 1.5)	**1.34** (**1, 1.81)**	1.19 (0.85, 1.67)	1.36 (0.95, 1.96)	1.15 (0.85, 1.57)	1.25 (0.9, 1.73)	Bloodletting therapy															
1.05 (0.89, 1.24)	1.07 (0.87, 1.33)	0.89 (0.75, 1.06)	0.88 (0.67, 1.14)	0.87 (0.7, 1.08)	0.89 (0.69, 1.15)	1 (0.79, 1.28)	0.93 (0.66, 1.33)	0.98 (0.72, 1.34)	0.82 (0.68, 0.99)	0.99 (0.82, 1.19)	0.88 (0.71, 1.07)	1 (0.81, 1.25)	0.85 (0.69, 1.05)	0.92 (0.73, 1.16)	0.74 (0.53, 1.02)	BLP-C														
0.98 (0.73, 1.32)	1.01 (0.73, 1.39)	0.84 (0.61, 1.14)	0.82 (0.57, 1.18)	0.81 (0.59, 1.13)	0.84 (0.59, 1.19)	0.94 (0.67, 1.32)	0.87 (0.57, 1.34)	0.92 (0.62, 1.37)	0.77 (0.56, 1.05)	0.93 (0.68, 1.26)	0.82 (0.58, 1.16)	0.94 (0.65, 1.36)	0.8 (0.57, 1.1)	0.86 (0.61, 1.21)	0.69 (0.46, 1.04)	0.94 (0.67, 1.31)	BLP-C + auricular point sticking													
1.08 (0.81, 1.44)	1.11 (0.81, 1.52)	0.93 (0.68, 1.24)	0.91 (0.63, 1.29)	0.9 (0.65, 1.23)	0.93 (0.65, 1.3)	1.04 (0.74, 1.45)	0.97 (0.64, 1.47)	1.02 (0.69, 1.5)	0.85 (0.62, 1.14)	1.03 (0.76, 1.37)	0.91 (0.69, 1.18)	1.04 (0.73, 1.48)	0.88 (0.64, 1.2)	0.95 (0.68, 1.32)	0.77 (0.51, 1.14)	1.04 (0.75, 1.41)	1.1 (0.73, 1.66)	BLP-C + autohemotherapy												
**1.25** (**1.05, 1.52)**	**1.28** (**1.03, 1.63)**	1.07 (0.88, 1.32)	1.05 (0.79, 1.39)	1.04 (0.83, 1.31)	1.07 (0.82, 1.4)	1.2 (0.94, 1.56)	1.12 (0.79, 1.61)	1.18 (0.86, 1.62)	0.98 (0.8, 1.22)	1.19 (0.97, 1.46)	1.05 (0.81, 1.37)	1.2 (0.9, 1.61)	1.02 (0.81, 1.28)	1.1 (0.86, 1.42)	0.89 (0.63, 1.24)	1.2 (0.94, 1.54)	1.28 (0.91, 1.82)	1.16 (0.83, 1.64)	BLP-C + TCM											
1.15 (0.93, 1.43)	1.18 (0.91, 1.53)	0.98 (0.78, 1.25)	0.96 (0.7, 1.3)	0.95 (0.74, 1.23)	0.98 (0.74, 1.31)	1.1 (0.84, 1.46)	1.02 (0.71, 1.5)	1.08 (0.77, 1.52)	0.9 (0.71, 1.14)	1.09 (0.86, 1.38)	0.97 (0.72, 1.29)	1.1 (0.81, 1.51)	0.93 (0.72, 1.2)	1.01 (0.77, 1.33)	0.81 (0.57, 1.15)	1.1 (0.83, 1.44)	1.17 (0.81, 1.69)	1.06 (0.74, 1.53)	0.92 (0.69, 1.22)	Moxibustion										
1.27 (0.95, 1.73)	1.3 (0.95, 1.82)	1.08 (0.8, 1.49)	1.06 (0.74, 1.54)	1.05 (0.76, 1.47)	1.08 (0.77, 1.55)	1.22 (0.87, 1.73)	1.13 (0.75, 1.75)	1.19 (0.81, 1.78)	0.99 (0.73, 1.37)	1.2 (0.89, 1.65)	1.07 (0.75, 1.52)	1.22 (0.84, 1.78)	1.03 (0.75, 1.44)	1.12 (0.8, 1.58)	0.9 (0.6, 1.36)	1.21 (0.87, 1.72)	1.29 (0.86, 1.98)	1.17 (0.78, 1.78)	1.01 (0.72, 1.44)	1.1 (0.77, 1.6)	Moxibustion + cupping therapy									
1.03 (0.86, 1.23)	1.06 (0.87, 1.29)	0.88 (0.72, 1.08)	0.86 (0.65, 1.14)	0.85 (0.68, 1.07)	0.88 (0.68, 1.14)	0.99 (0.77, 1.27)	0.92 (0.65, 1.32)	0.97 (0.7, 1.33)	0.81 (0.66, 0.99)	0.98 (0.8, 1.19)	0.87 (0.67, 1.12)	0.99 (0.74, 1.32)	0.84 (0.67, 1.03)	0.91 (0.71, 1.15)	0.73 (0.52, 1.01)	0.98 (0.77, 1.25)	1.05 (0.74, 1.49)	0.95 (0.68, 1.34)	0.82 (0.63, 1.06)	0.9 (0.68, 1.19)	0.81 (0.57, 1.14)	Moxibustion + TCM								
**1.16** (**1.01, 1.34)**	1.19 (0.98, 1.46)	0.99 (0.84, 1.17)	0.97 (0.75, 1.25)	0.96 (0.79, 1.17)	0.99 (0.78, 1.26)	1.11 (0.89, 1.4)	1.03 (0.75, 1.46)	1.09 (0.81, 1.47)	0.91 (0.77, 1.08)	1.1 (0.93, 1.3)	0.98 (0.77, 1.23)	1.11 (0.86, 1.45)	0.95 (0.78, 1.14)	1.02 (0.82, 1.27)	0.82 (0.6, 1.12)	1.11 (0.89, 1.38)	1.18 (0.85, 1.64)	1.07 (0.78, 1.48)	0.93 (0.73, 1.16)	1.01 (0.78, 1.31)	0.92 (0.65, 1.26)	1.13 (0.9, 1.42)	ACE							
**1.16** (**1.06, 1.28)**	**1.19** (**1.03, 1.4)**	1 (0.87, 1.13)	0.97 (0.77, 1.23)	0.96 (0.82, 1.14)	1 (0.8, 1.23)	1.12 (0.92, 1.36)	1.04 (0.76, 1.44)	1.09 (0.83, 1.44)	0.91 (0.79, 1.05)	1.1 (0.97, 1.25)	0.98 (0.79, 1.2)	1.12 (0.88, 1.43)	0.95 (0.81, 1.1)	1.03 (0.84, 1.24)	0.82 (0.61, 1.1)	1.11 (0.92, 1.35)	1.19 (0.87, 1.62)	1.07 (0.8, 1.46)	0.93 (0.75, 1.14)	1.02 (0.8, 1.28)	0.92 (0.67, 1.24)	1.13 (0.93, 1.38)	1 (0.85, 1.19)	ACE + TCM						
1.13 (0.89, 1.45)	1.16 (0.88, 1.54)	0.97 (0.75, 1.26)	0.95 (0.68, 1.31)	0.94 (0.71, 1.25)	0.97 (0.71, 1.32)	1.09 (0.81, 1.47)	1.01 (0.69, 1.5)	1.07 (0.75, 1.52)	0.89 (0.68, 1.16)	1.07 (0.83, 1.39)	0.95 (0.7, 1.29)	1.09 (0.78, 1.52)	0.92 (0.7, 1.21)	1 (0.74, 1.34)	0.8 (0.55, 1.16)	1.08 (0.81, 1.46)	1.16 (0.79, 1.7)	1.05 (0.72, 1.53)	0.9 (0.66, 1.23)	0.99 (0.71, 1.37)	0.89 (0.6, 1.31)	1.1 (0.81, 1.49)	0.98 (0.74, 1.3)	0.97 (0.75, 1.27)	Fire needle + TCM					
1.23 (0.88, 1.78)	1.26 (0.88, 1.87)	1.05 (0.74, 1.53)	1.03 (0.69, 1.57)	1.02 (0.71, 1.51)	1.05 (0.71, 1.58)	1.18 (0.81, 1.78)	1.1 (0.7, 1.77)	1.16 (0.75, 1.81)	0.96 (0.68, 1.41)	1.17 (0.82, 1.7)	1.04 (0.7, 1.56)	1.18 (0.79, 1.82)	1 (0.69, 1.47)	1.08 (0.74, 1.61)	0.87 (0.56, 1.37)	1.18 (0.8, 1.76)	1.26 (0.8, 2)	1.14 (0.73, 1.82)	0.98 (0.67, 1.48)	1.07 (0.72, 1.64)	0.97 (0.61, 1.55)	1.19 (0.81, 1.8)	1.06 (0.73, 1.57)	1.06 (0.74, 1.54)	1.09 (0.71, 1.69)	Warm-needle moxibustion				
1.06 (0.95, 1.18)	1.08 (0.92, 1.27)	0.9 (0.78, 1.04)	0.88 (0.69, 1.12)	0.87 (0.74, 1.03)	0.9 (0.72, 1.12)	1.01 (0.83, 1.24)	0.94 (0.68, 1.31)	0.99 (0.75, 1.31)	0.83 (0.71, 0.95)	1 (0.88, 1.14)	0.89 (0.71, 1.1)	1.01 (0.79, 1.3)	0.86 (0.73, 1.01)	0.93 (0.76, 1.13)	0.75 (0.55, 1)	1.01 (0.83, 1.23)	1.08 (0.79, 1.47)	0.97 (0.72, 1.33)	0.84 (0.67, 1.04)	0.92 (0.72, 1.17)	0.83 (0.6, 1.13)	1.02 (0.85, 1.24)	0.91 (0.76, 1.08)	0.91 (0.79, 1.04)	0.93 (0.71, 1.21)	0.86 (0.58, 1.22)	TCM			
1.32 (0.99, 1.79)	1.35 (0.98, 1.9)	1.13 (0.83, 1.55)	1.1 (0.77, 1.6)	1.09 (0.79, 1.53)	1.13 (0.8, 1.61)	1.27 (0.91, 1.8)	1.18 (0.78, 1.82)	1.24 (0.84, 1.85)	1.03 (0.76, 1.42)	1.25 (0.92, 1.71)	1.11 (0.78, 1.58)	1.27 (0.88, 1.85)	1.07 (0.78, 1.49)	1.16 (0.83, 1.64)	0.93 (0.62, 1.41)	1.26 (0.9, 1.78)	1.35 (0.89, 2.06)	1.22 (0.81, 1.85)	1.05 (0.74, 1.5)	1.15 (0.8, 1.67)	1.04 (0.68, 1.59)	1.28 (0.91, 1.82)	1.14 (0.83, 1.59)	1.13 (0.84, 1.56)	1.16 (0.8, 1.72)	1.07 (0.67, 1.69)	1.25 (0.92, 1.73)	TCM + auricular point sticking		
**1.46** (**1.19, 1.84)**	1.5 (1.18, 1.96)	**1.25** (**1.01, 1.58)**	1.22 (0.91, 1.66)	1.21 (0.95, 1.58)	1.25 (0.95, 1.68)	1.41 (1.08, 1.87)	1.31 (0.91, 1.92)	1.38 (0.99, 1.94)	1.15 (0.91, 1.47)	**1.39** (**1.12, 1.75)**	1.23 (0.93, 1.64)	**1.41** (**1.04, 1.93)**	1.19 (0.93, 1.54)	1.29 (0.99, 1.7)	1.04 (0.73, 1.48)	1.4 (1.08, 1.84)	**1.49** (**1.04, 2.17)**	1.35 (0.96, 1.95)	1.17 (0.88, 1.56)	1.28 (0.95, 1.74)	1.16 (0.8, 1.67)	**1.42** (**1.09, 1.89)**	1.26 (0.98, 1.65)	1.26 (1, 1.61)	1.29 (0.94, 1.8)	1.19 (0.79, 1.79)	**1.39** (**1.1, 1.78)**	1.11 (0.77, 1.6)	Acupuncture + autohemotherapy	
1.03 (0.76, 1.38)	1.05 (0.76, 1.46)	0.88 (0.64, 1.2)	0.86 (0.59, 1.24)	0.85 (0.61, 1.18)	0.88 (0.62, 1.25)	0.99 (0.7, 1.39)	0.92 (0.6, 1.41)	0.97 (0.65, 1.43)	0.81 (0.59, 1.1)	0.97 (0.71, 1.32)	0.86 (0.61, 1.22)	0.99 (0.68, 1.43)	0.84 (0.6, 1.15)	0.9 (0.64, 1.27)	0.73 (0.48, 1.09)	0.98 (0.7, 1.38)	1.05 (0.69, 1.59)	0.95 (0.63, 1.44)	0.82 (0.58, 1.16)	0.9 (0.62, 1.29)	0.81 (0.53, 1.22)	1 (0.71, 1.41)	0.88 (0.64, 1.23)	0.88 (0.65, 1.2)	0.91 (0.62, 1.33)	0.83 (0.52, 1.31)	0.97 (0.71, 1.33)	0.78 (0.51, 1.18)	0.7 (0.48, 1)	Autohemotherapy + point application

Note: The data in the table are the RR (95CrI) of the intervention in the row compared to the intervention in the column. A larger RR represents a higher Therapeutic effect, 1 represents no difference between the two interventions. Bolding represents a statistically significant increase in Therapeutic Effect.

The results of SUCRA probability ranking indicated the following order: acupuncture combined with autohemotherapy (92.79%) > BLT (82.35%) > moxibustion combined with cupping therapy (81.72%). Acupuncture combined with autohemotherapy demonstrated superior efficacy in treating CSU (Figure 5).

**Figure 5:**
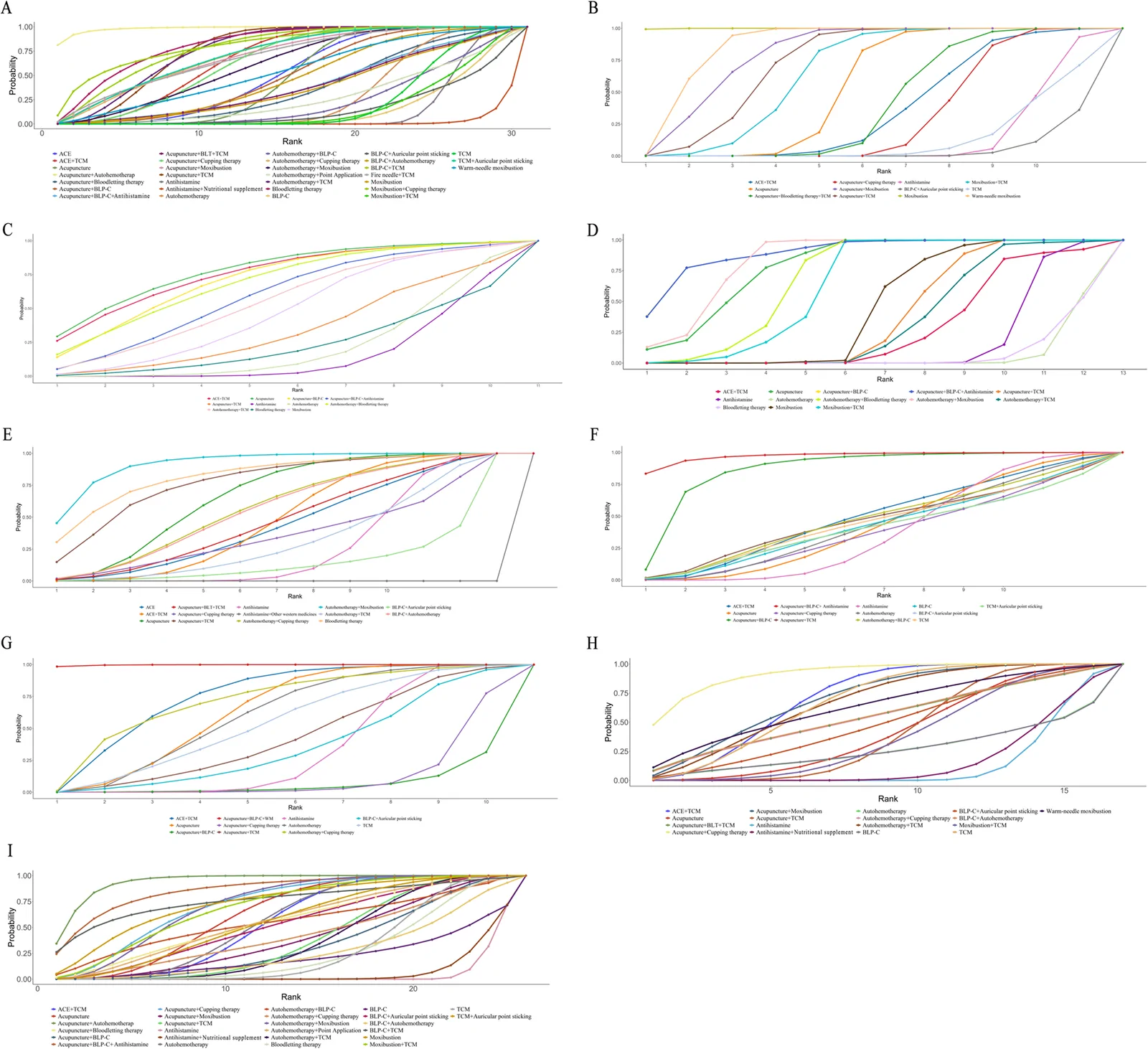
**(A)** SUCRA values for the effect of different interventions on treatment efficacy; **(B)** SUCRA values for the effect of different interventions on DLQI score; **(C)** SUCRA values for the effect of different interventions on wheal count; **(D)** SUCRA values for the effect of different interventions on the severity of pruritus; **(E)** SUCRA values for the effect of different interventions on IgE concentration; **(F)** SUCRA values for the effect of different interventions on IL-4 concentration; **(G)** SUCRA values for the effect of different interventions on IFN-γ concentration; **(H)** SUCRA values for the effect of different interventions on adverse reactions; **(I)** SUCRA values for the effect of different interventions on recurrence rate.

#### 3.3.3 DLQI score

In total, 12 studies reported the DLQI score. The NMA results showed that moxibustion significantly decreased DLQI scores compared with antihistamine [moxibustion vs. antihistamine: MD = −9.45, 95% CrI = (−12.48, −6.44)] (Supplementary Table S5).

The results of SUCRA probability ranking indicated the following order: moxibustion (99.94%) > warm-needle moxibustion (86.80%) > acupuncture combined with moxibustion (80.39%). Moxibustion alone demonstrated superior efficacy in lowering DLQI scores (Figure 5).

#### 3.3.4 Wheal count

In total, 13 studies reported wheal counts. The NMA results showed that most pairwise comparisons among interventions revealed no significant differences, and none of the acupuncture therapies demonstrated statistical superiority over antihistamines (Supplementary Table S6).

The results of SUCRA probability ranking indicated the following order: acupuncture (81.29%) > ACE combined with traditional Chinese medicine (TCM) (79.09%) > autohemotherapy combined with BLT (71.69%) (Figure 5).

#### 3.3.5 Severity of pruritus

In total, 16 studies reported the severity of pruritus. The NMA results showed that, compared with antihistamine, acupuncture combined with moxibustion significantly decreased the severity of pruritus [acupuncture combined with moxibustion vs. antihistamine: MD = −1.03, 95% CrI = (−1.15, −0.91)] (Supplementary Table S7).

The results of SUCRA probability ranking indicated the following order: acupuncture combined with moxibustion (96.27%) > acupuncture (91.87%) > autohemotherapy combined with BLT (83.31%). Acupuncture combined with moxibustion may be superior to other interventions in decreasing the severity of pruritus (Figure 5).

#### 3.3.6 IgE

In total, 27 studies reported IgE. The NMA results showed that, among patients with CSU, autohemotherapy combined with moxibustion significantly reduced the levels of IgE compared with antihistamine [autohemotherapy combined with moxibustion vs. antihistamine: MD = −36.23, 95% CrI = (−55.33, −17.33)] (Table 2).

**Table 2:** Pairwise comparison results of varied interventions on IgE concentration.

Antihistamine													
153.61 (123.37, 183.7)	Antihistamine + nutritional supplements												
**−15.9** (**−33.03, −1.01)**	**−169.53** (**−204.7, −136.46)**	Acupuncture											
−0.76 (−37.3, 35.59)	**−154.25** (**−201.31, −107.42)**	15.31 (−24.1, 55.34)	Acupuncture + cupping therapy										
−7.6 (−32.21, 16.79)	**−161.23** (**−199.69, −122.42)**	8.33 (−19.8, 38.43)	−6.84 (−50.32, 36.8)	Acupuncture + BLT + TCM									
**−25.21** (**−53.11, 2.72)**	**−178.77** (**−219.79, −137.89)**	−9.21 (−40.43, 23.75)	**−24.47** (**−70.05, 21.47)**	**−17.59** (**−54.67, 19.52)**	Acupuncture + TCM								
**−12.43** (**−37.49, 12.5)**	**−166.05** (**−205.03, −127.14)**	3.47 (−25.1, 33.98)	−11.71 (−55.36, 32.07)	−4.86 (−39.89, 30.23)	12.73 (−24.27, 50.04)	Autohemotherapy + cupping therapy							
−0.94 (−24.35, 22.27)	**−154.53** (**−192.69, −116.58)**	14.95 (−12.16, 44.19)	−0.14 (−43.14, 42.73)	6.7 (−27.1, 40.35)	24.27 (−11.92, 60.51)	11.52 (−22.5, 45.43)	Autohemotherapy + TCM						
**−36.23** (**−55.33, −17.33)**	**−189.79** (**−213.46, −166.32)**	**−20.26** (**−44.23, 5.45)**	**−35.47** (**−76.36, 5.64)**	**−28.62** (**−59.45, 2.28)**	**−11.06** (**−44.89, 22.75)**	**−23.8** (**−55.03, 7.18)**	**−35.27** (**−65.44, −5.35)**	Autohemotherapy + moxibustion					
**−30.65** (**−63.12, 2.03)**	**−184.18** (**−228.17, −140.12)**	−14.63 (−50, 22.28)	**−29.89** (**−78.65, 19.15)**	**−22.97** (**−63.65, 17.71)**	−5.45 (−48.19, 37.44)	**−18.13** (**−59.1, 22.61)**	−29.71 (−69.53, 10.38)	5.64 (−32.03, 43.42)	Bloodletting therapy				
14.16 (−19.07, 47.31)	**−139.39** (**−184.04, −94.64)**	30.2 (−5.94, 67.69)	14.94 (−34.48, 64.24)	21.71 (−19.2, 62.68)	39.33 (−4.07, 82.76)	26.61 (−14.68, 67.99)	15.09 (−24.95, 55.44)	50.42 (12.13, 88.51)	44.8 (−1.61, 91.18)	BLP-C + auricular point sticking			
**−12.06** (**−36.99, 12.86)**	**−165.62** (**−204.52, −126.5)**	3.86 (−24.64, 34.6)	−11.29 (−54.98, 32.67)	−4.4 (−39.57, 30.49)	13.12 (−23.94, 50.36)	0.41 (−24.33, 25.05)	**−11.09** (**−45.09, 23.03)**	24.17 (−6.86, 55.37)	18.53 (−22.17, 59.71)	−26.23 (−67.62, 15.09)	BLP-C + autohemotherapy		
−6.28 (−30.34, 18.01)	**−159.88** (**−198.17, −121.27)**	9.66 (−18.43, 39.92)	−5.51 (−48.9, 38)	1.28 (−33.11, 36.16)	18.93 (−17.72, 55.93)	6.21 (−28.31, 40.77)	−5.33 (−38.68, 28.21)	29.93 (−0.54, 60.53)	24.37 (−16.08, 64.76)	**−20.4** (**−60.99, 20.53)**	5.76 (−28.78, 40.53)	ACE	
−9.46 (−21.63, 1.66)	**−163.09** (**−195.7, −131.14)**	6.44 (−12.76, 26.89)	−8.78 (−47.18, 29.14)	−1.89 (−29.42, 24.87)	15.65 (−14.75, 45.6)	2.94 (−24.84, 30.17)	−8.5 (−34.93, 17.2)	26.71 (4.27, 48.64)	21.09 (−13.8, 55.34)	**−23.71** (**−58.98, 11.08)**	2.59 (−25.3, 29.58)	−3.2 (−30.54, 23.19)	ACE + TCM

Note: The data in the table are the MD (95CrI) of the intervention in the row compared to the intervention in the column. A lower mean difference indicates lower IgE levels, with 0 signifying no difference between the two interventions. Bold text indicates a significant reduction in IgE levels.

The results of SUCRA probability ranking indicated the following order: autohemotherapy combined with moxibustion (93.05%) > BLT (83.80%) > acupuncture combined with TCM (78.84%). Autohemotherapy combined with moxibustion demonstrated the greatest reduction in the levels of IgE (Figure 5).

#### 3.3.7 IL-4

In total, 18 studies reported IL-4. The NMA results showed that, compared with antihistamine, the combination of acupuncture, BLT puncture and cupping (BLP-C), and antihistamine significantly reduced the levels of IL-4 [the combination of acupuncture, BLP-C, and antihistamine vs. antihistamine: MD = −194.39, 95% CrI = (−317.68, −71.1)] (Supplementary Table S8).

The results of SUCRA probability ranking indicated the following order: the combination of acupuncture, BLP-C, and antihistamine (97.95%) > acupuncture combined with BLP-C (87.64%) > ACE combined with TCM (46.94%). The combination of acupuncture, BLP-C, and antihistamine exhibited the greatest reduction in the levels of IL-4 (Figure 5).

#### 3.3.8 IFN-γ

In total, 16 studies reported IFN-γ. The NMA results showed that, relative to antihistamine, the combination of acupuncture, BLP-C, and antihistamine significantly increased the levels of IFN-γ [the combination of acupuncture, BLP-C, and antihistamine vs. antihistamine: MD = 32.36, 95% CrI = (19, 46.49)] (Supplementary Table S9).

The results of SUCRA probability ranking indicated the following order: the combination of acupuncture, BLP-C, and antihistamine (99.78%) > ACE combined with TCM (75.09%) > autohemotherapy combined with cupping therapy (71.48%). The combination of acupuncture, BLP-C, and antihistamine achieved the greatest increase in the levels of IFN-γ (Figure 5).

#### 3.3.9 Adverse reactions

In total, 30 studies reported adverse reactions. The NMA results showed that, among patients with CSU, acupuncture combined with cupping therapy significantly diminished the occurrence of adverse reactions compared with antihistamine [acupuncture combined with cupping therapy vs. antihistamine: RR = 0.04, 95% CrI = (0.04, 0.24)] (Table 3).

**Table 3:** Pairwise comparison results of varied interventions on adverse reactions.

Antihistamine																
0.91 (0.34, 2.49)	Antihistamine + nutritional supplements															
0.39 (0.11, 1.14)	0.42 (0.09, 1.79)	Acupuncture														
**0.14** (**0.03, 0.53)**	**0.16** (**0.02, 0.82)**	0.37 (0.05, 2.32)	Acupuncture + moxibustion													
**0.04** (**0, 0.24)**	**0.04** (**0, 0.33)**	**0.1** (**0, 0.91)**	0.25 (0.01, 3.11)	Acupuncture + cupping therapy												
0.25 (0.01, 2.38)	0.26 (0.01, 3.22)	0.62 (0.02, 8.76)	1.7 (0.04, 26.95)	6.81 (0.13, 368.8)	Acupuncture + BLT + TCM											
**0.38** (**0.2, 0.7)**	0.42 (0.14, 1.21)	0.99 (0.31, 3.65)	2.68 (0.62, 14.99)	10.52 (1.44, 303.24)	1.57 (0.15, 50.82)	Acupuncture + TCM										
0.97 (0.02, 38.73)	1.08 (0.02, 49.85)	2.59 (0.05, 124.09)	6.97 (0.14, 379.44)	29.45 (0.44, 3,950.26)	4.44 (0.05, 610.26)	2.55 (0.06, 109.58)	Autohemotherapy									
0.24 (0.01, 2.5)	0.26 (0.01, 3.36)	0.63 (0.02, 8.74)	1.7 (0.05, 28)	6.97 (0.14, 376.75)	1 (0.02, 60.95)	0.64 (0.02, 7.03)	0.23 (0, 18.62)	Autohemotherapy + cupping therapy								
**0.16** (**0.03, 0.67)**	**0.18** (**0.05, 0.52)**	0.42 (0.06, 2.66)	1.14 (0.15, 9.6)	4.57 (0.38, 161.24)	0.68 (0.04, 27.14)	0.42 (0.08, 1.87)	0.16 (0, 8.32)	0.68 (0.04, 24.39)	Autohemotherapy + TCM							
0.98 (0.03, 39.39)	1.06 (0.02, 51.09)	2.59 (0.06, 124.98)	7.05 (0.14, 376.61)	29.59 (0.44, 3,583.27)	4.33 (0.06, 575.86)	2.56 (0.06, 110.17)	1 (0.01, 188.55)	4.43 (0.05, 516.39)	6.13 (0.12, 329.98)	BLP-C						
0.32 (0.04, 1.68)	0.34 (0.03, 2.44)	0.82 (0.08, 6.5)	2.2 (0.19, 21.92)	8.77 (0.5, 341.13)	1.29 (0.06, 58.62)	0.81 (0.09, 4.87)	0.31 (0, 17.42)	1.29 (0.06, 54.85)	1.92 (0.15, 19.26)	0.3 (0, 17.37)	BLP-C + auricular point sticking					
0.24 (0.01, 2.44)	0.26 (0.01, 3.35)	0.62 (0.02, 8.63)	1.66 (0.05, 29.05)	6.76 (0.15, 350.85)	0.99 (0.02, 59.27)	0.63 (0.02, 7.03)	0.23 (0, 18.81)	0.99 (0.03, 41.05)	1.47 (0.04, 25.17)	0.23 (0, 18.45)	0.76 (0.02, 17.98)	BLP-C + autohemotherapy				
0.43 (0.12, 1.55)	0.47 (0.19, 1.11)	1.11 (0.21, 6.46)	3.03 (0.47, 23.33)	11.93 (1.22, 400.8)	1.8 (0.13, 69.46)	1.11 (0.29, 4.35)	0.44 (0.01, 21.42)	1.8 (0.13, 64.29)	2.63 (0.66, 12.58)	0.44 (0.01, 20.61)	1.37 (0.16, 15.53)	1.8 (0.12, 63.86)	Moxibustion + TCM			
**0.16** (**0.07, 0.33)**	**0.17** (**0.06, 0.44)**	0.4 (0.1, 1.67)	1.08 (0.22, 6.4)	4.25 (0.54, 132.28)	0.63 (0.06, 21.74)	0.4 (0.15, 1.06)	0.16 (0, 6.77)	0.64 (0.05, 20.25)	0.95 (0.22, 4.58)	0.16 (0, 6.64)	0.49 (0.08, 4.57)	0.65 (0.06, 19.73)	0.36 (0.1, 1.3)	ACE + TCM		
0.17 (0.01, 1.5)	0.18 (0.01, 2.08)	0.44 (0.01, 5.54)	1.17 (0.03, 18.32)	4.74 (0.09, 242.04)	0.69 (0.01, 39.92)	0.44 (0.01, 4.33)	0.16 (0, 12.25)	0.7 (0.01, 36.63)	1.01 (0.03, 15.1)	0.16 (0, 12.14)	0.54 (0.01, 10.94)	0.7 (0.01, 38.7)	0.39 (0.01, 5.03)	1.09 (0.03, 11.12)	Warm-needle moxibustion	
**0.17** (**0.05, 0.48)**	**0.19** (**0.06, 0.53)**	0.44 (0.09, 2.17)	1.19 (0.21, 8.12)	4.63 (0.57, 136.41)	0.71 (0.05, 25.79)	0.44 (0.12, 1.44)	0.17 (0, 8.05)	0.71 (0.05, 21.89)	1.04 (0.22, 5.28)	0.17 (0, 7.5)	0.55 (0.07, 5.44)	0.71 (0.05, 23.64)	0.4 (0.1, 1.36)	1.1 (0.31, 3.68)	1 (0.08, 32.58)	TCM

Note: The data in the table are the RR (95CrI) of the intervention in the row compared to the intervention in the column. A smaller relative risk value indicates a lower incidence of adverse reactions, while a value of 1 indicates no difference between the two interventions. Bold text indicates a statistically significant reduction in the incidence of adverse reactions.

The results of SUCRA probability ranking indicated the following order: acupuncture combined with cupping therapy (91.69%) > acupuncture combined with moxibustion (70.88%) > ACE combined with TCM (70.76%). Acupuncture combined with cupping therapy exhibited the fewest adverse reactions, while antihistamine alone triggered the most adverse reactions (Figure 5).

#### 3.3.10 Relapse rate

In total, 44 studies reported relapse rates. The NMA results showed that, compared with antihistamine, acupuncture combined with autohemotherapy significantly decreased the relapse rate [acupuncture combined with autohemotherapy vs. antihistamine: RR = 0.02, 95% CrI = (0, 0.09)] (Supplementary Table S10).

The results of SUCRA probability ranking indicated the following order: acupuncture combined with cupping therapy (91.79%) > acupuncture combined with moxibustion (70.68%) > ACE combined with TCM (70.65%). Acupuncture combined with cupping therapy resulted in the lowest relapse rate (Figure 5).

In addition, this study used a Bayesian meta-regression model to specifically examine whether follow-up duration moderated the effects on relapse rate. At a mean follow-up of approximately 4.6 months, the pooled effect size was −2.479 (95% CrI: −4.102, −1.057) for antihistamines, −1.725 (95% CrI: −2.353, −1.134) for acupuncture combined with cupping therapy, and −1.087 (95% CrI: −1.699, −0.467) for ACE combined with TCM. None of these CrIs included zero, suggesting that these interventions were significantly associated with a reduced risk of relapse at typical follow-up durations. Further analysis of the regression slopes over the range of follow-up durations showed that the 95% CrIs of all slope coefficients included zero: beta (5) for antihistamines was −0.576 (95% CrI: −5.403, 3.554), beta (26) for acupuncture combined with cupping therapy was 5.727 (95% CrI: −14.130, 102.437), and beta (22) for ACE combined with TCM was 0.228 (95% CrI: −0.821, 1.214). This finding indicated that follow-up duration had no statistically significant moderating effect on the effect sizes of the interventions. In addition, between-study heterogeneity was very low (*I*^2^ = 0%; posterior mean of sd.d = 0.208, 95% CrI: 0.006, 0.591), indicating high consistency across studies and indirectly supporting the conclusion that the effects were relatively stable over the observed time range.

#### 3.3.11 Publication bias

Publication bias was assessed by plotting adjusted comparison funnel plots. The results showed that the funnel plots were symmetric, indicating no significant publication bias (Figure 6).

**Figure 6:**
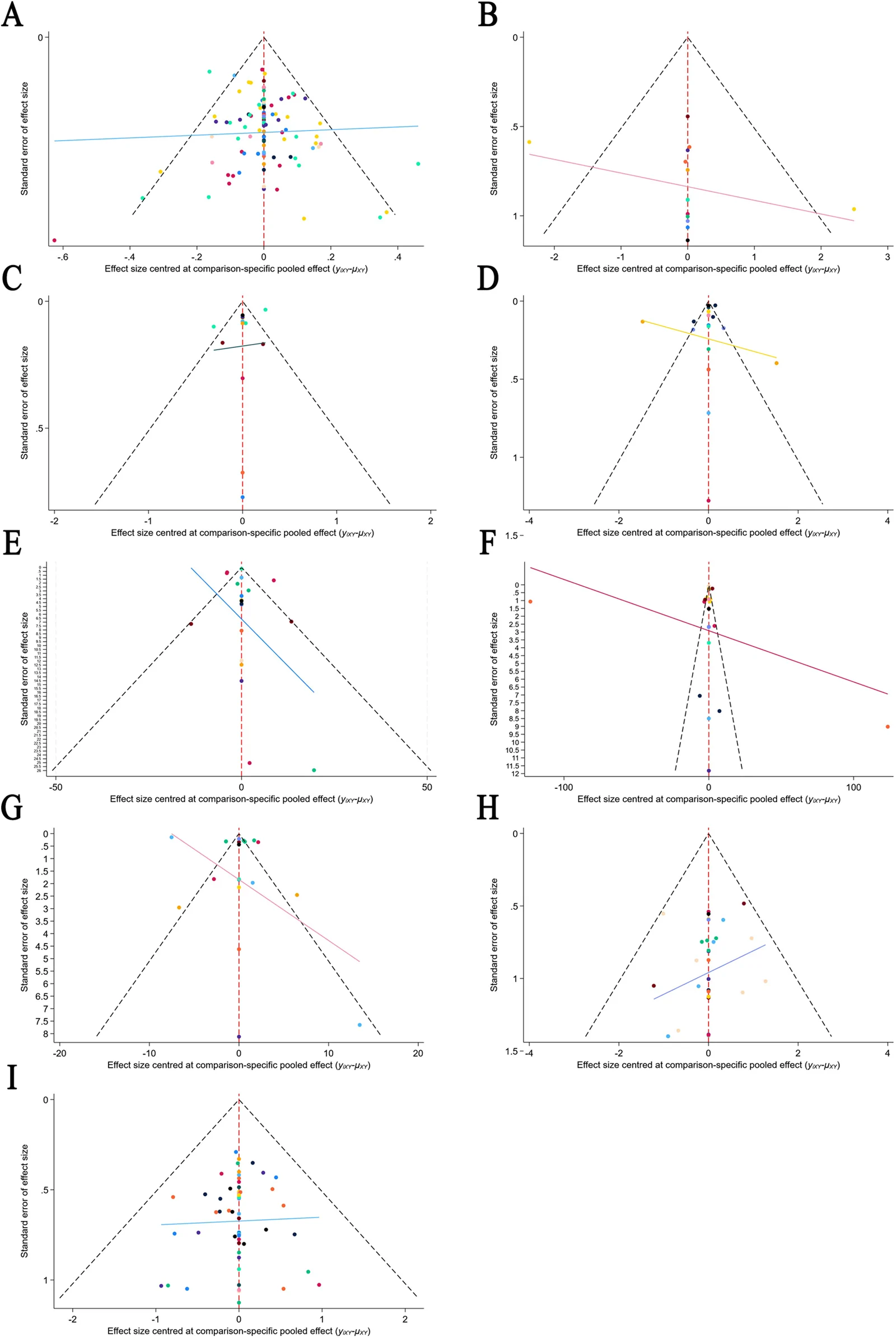
Funnel plot. **(A)** Efficacy; **(B)** DLQI; **(C)** wheal count; **(D)** severity of pruritus; **(E)** IgE; **(F)** IL-4; **(G)** IFN-γ; **(H)** adverse reactions; **(I)** relapse rate.

## 4 Discussion

Acupuncture therapies demonstrate marked efficacy in alleviating clinical symptoms, enhancing QoL, regulating immune function, and improving long-term prognosis in individuals with CSU. With regard to clinical symptoms, combined acupuncture and moxibustion can effectively diminish wheal counts and alleviate the severity of pruritus. Acupuncture can reduce local inflammatory responses, while moxibustion has a warming and soothing effect. Combined acupuncture and moxibustion relieves the core symptoms of CSU, including pruritus, the formation of wheals, and vascular edema. In terms of QoL, moxibustion significantly decreased the DLQI score, indicating that it effectively improves the overall QoL of patients. Moxibustion improves overall comfort by regulating autonomic nervous function and exerting anti-inflammatory effects, thereby enhancing the QoL of patients (120). With regard to improvement in serum total IgE levels, combined autohemotherapy and moxibustion treatment may become the optimal intervention. Autohemotherapy reduces allergic reactions by inducing immune tolerance (induction of anti-idiotypes) (121, 122). Meanwhile, moxibustion enhances its regulatory effects. For the reduction of pro-inflammatory factors IL-4 and IFN-γ, the combination of acupuncture, BLP-C, and antihistamine is likely to be the most effective intervention. Combined acupuncture and BLP-C can correct the imbalance of Th1 and Th2 in serum (111) and lower the levels of IL-4 and IFN-γ, diminishing the release of cell mediators and vascular permeability. The combination of acupuncture, BLP-C, and antihistamine can enhance the anti-inflammatory effects. With regard to overall therapeutic efficacy, combined acupuncture and autohemotherapy demonstrates a significantly elevated overall effectiveness relative to the control group, indicating that it might be the most favorable intervention. For safety, combined acupuncture and cupping therapy is associated with the lowest incidence of adverse reactions, indicating that it is highly safe and suitable for long-term use. In terms of long-term prognosis, combined acupuncture and autohemotherapy is associated with the lowest relapse rate, suggesting its suitability for patients requiring sustained therapy or those with medication intolerance. The marked improvement in wheal count and pruritus observed in this study aligns with the findings reported by Zheng et al. (123), Bi et al. (124), and Wei et al. (125).

### 4.1 Mechanisms of acupuncture therapy in improving clinical symptoms of CSU

The clinical efficacy of acupuncture therapy in treating CSU is closely associated with its multi-target immune regulatory effects. In diminishing wheal count and alleviating the severity of pruritus, combined acupuncture and moxibustion is the most effective. Studies have shown that imbalance in Th1 and Th2 can induce urticaria (126). Acupuncture can reduce the secretion of both Th1 and Th2 cytokines (127), thereby maintaining immune balance. Mast cells are key effector cells in the pathogenesis of CSU. Upon activation, they degranulate and release various inflammatory mediators, causing vasodilation, plasma extravasation, wheals, and pruritus (128). Acupuncture can regulate the immune balance of Th1 and Th2 and inhibit the degranulation of mast cells (127). Moxibustion exerts warming and dredging effects, inhibiting mast cells from releasing inflammatory mediators such as histamine and leukotrienes (129). Combined acupuncture and moxibustion effectively suppresses the inflammatory response, reduces vascular permeability, decreases the wheal count, and alleviates pruritus. Regarding overall therapeutic efficacy, combined acupuncture and autohemotherapy demonstrates superior efficacy among the interventions. Moxibustion significantly lowers the DLQI scores of patients. Autohemotherapy can continuously stimulate acupoints, enhance the effects of the acupoints, inhibit the infiltration of mast cells, expose antigen components in the blood, and activate the immune system, thereby producing a desensitization effect (130). Combined acupuncture and autohemotherapy regulates the immune status of individuals with CSU, improves clinical symptoms, and enhances the QoL of patients.

### 4.2 Mechanisms of acupuncture therapy in regulating IgE, IFN-γ, and IL-4 Levels

IgE is a key mediator in type I hypersensitivity, and IgE-mediated type I allergic reactions constitute one of the primary mechanisms of CSU (131). Combined autohemotherapy and moxibustion, BLT, and acupuncture can significantly reduce serum total IgE levels. Th17 cells and regulatory T cells (Tregs) are crucial for maintaining immune homeostasis. An increased Th17/Treg ratio might promote the production of IgE, thereby triggering urticaria (132, 133). Acupuncture can upregulate the expression of transforming growth factor-beta (TGF-β) and forkhead box protein 3 (Foxp3), reduce IL-17 expression, and correct the imbalance of Th17 and Treg, thereby lowering IgE levels (134). Clinical observations have suggested that patients with CSU receiving acupuncture treatment exhibit a reduction in serum IgE levels and significant improvements in the frequency of wheals and the severity of pruritus (135). The combination of acupuncture, BLP-C, and antihistamine has the most favorable effect on regulating the levels of IFN-γ and IL-4, followed by combined autohemotherapy and cupping therapy. In an animal experiment using type I and type IV skin hypersensitivity models, Han (127) observed that acupuncture reduced the secretion of Th1 and Th2 cytokines, maintained the balance of Th1 and Th2, and exhibited significant bidirectional regulation by increasing IFN-γ levels and decreasing IL-4 levels. An experiment by Zeng et al. (130) suggested that autohemotherapy inhibits the degranulation of diseased mast cells, downregulates IL-4 levels in mouse serum, and upregulates the IFN-γ/IL-4 ratio. The combination of acupuncture, BLP-C, and antihistamine can rapidly control the levels of IFN-γ and IL-4, improving symptoms such as pruritus and wheals in patients with CSU.

### 4.3 Safety of acupuncture therapy

Acupuncture combined with cupping therapy and acupuncture combined with moxibustion demonstrate minimal adverse reactions. The primary adverse reactions of acupuncture include pain at the acupuncture site, local hematoma, or bruising, which typically resolve spontaneously. Cupping therapy may cause localized ecchymosis and a transient burning sensation at cup sites, which generally subside within a short period. The main risk of moxibustion lies in potential burns from improper operation. Compared with pharmacotherapy, these treatments avoid drug toxicities, resistance, and dependency associated with medication, thus demonstrating superior overall safety profiles.

Long-term follow-up confirmed that the relapse rate of acupuncture combined with autohemotherapy is significantly lower compared with antihistamines. This finding may be linked to the sustained immune regulatory effects of this approach through the neuro-endocrine-immune network. Acupuncture can improve the immune microenvironment by inhibiting the excitability of sympathetic nervous system and enhancing the activity of parasympathetic nervous system. Studies have indicated that stimulating Zusanli (ST36) activates the vagal-adrenal pathway, promoting the release of anti-inflammatory factors from dopaminergic neurons and suppressing systemic inflammation (136). Autohemotherapy stimulates the body to produce a non-specific immune response, reducing the sensitivity of the body to allergens. The combined application of the two approaches can relieve symptoms and may lower the relapse rate in the long term.

### 4.4 Clinical significance and application value

This study systematically evaluated the efficacy and safety of various acupuncture therapies, used alone or in combination with antihistamines, in patients with CSU through an NMA. Overall, combination therapies showed advantages in improving clinical symptoms, regulating immune markers, and reducing relapse rates. In particular, acupuncture combined with autohemotherapy, acupuncture combined with moxibustion, and acupuncture combined with cupping therapy ranked highly across multiple outcome measures, suggesting that integrated acupuncture-based interventions may hold important potential value in the management of CSU.

Compared with previous studies, the present findings further expand the evidence for the application of TCM therapies in CSU. A recent systematic review of autohemotherapy combined with external TCM therapies also confirmed that combination treatment significantly increased the total effective rate (RR = 1.17, 95% CI: 1.10–1.24) and reduced the relapse rate (RR = 0.31, 95% CI: 0.23–0.42) (137).

That study also found that autohemotherapy combined with other TCM treatments reduced IgE levels (SMD = −1.33), consistent with the results of autohemotherapy combined with moxibustion in the present study. However, previous reviews primarily focused on comparisons between a single TCM therapy and antihistamines. By constructing a multi-intervention network, this study revealed the relative ranking of different combination therapies, providing a more nuanced reference for clinical decision-making. Furthermore, acupuncture combined with autohemotherapy performed prominently in terms of both efficacy and relapse rates in this study, consistent with previous findings. However, this combination has rarely been systematically summarized in the existing studies, suggesting that it may be an underappreciated but potentially preferred strategy.

In terms of the certainty of evidence, several recent NMAs have raised concerns about the quality of studies in this field. One NMA including 50 RCTs with 5,814 patients used the Confidence in Network Meta-Analysis framework to assess the quality of evidence and found that the certainty of efficacy outcomes was mainly moderate, while the overall certainty of safety outcomes was low (138). That study also reported that most combination regimens were superior to antihistamine monotherapy in terms of efficacy, but improvements in some immune markers, such as IgE and IL-4, did not show statistical superiority. Their findings are consistent with the wide CrIs observed for some immune outcomes in the present study. In the present study, inconsistency was observed for efficacy outcomes, suggesting possible conflicts between direct and indirect evidence. This inconsistency may have affected the credibility of these outcomes. In addition, the wide CrIs for most comparisons reflect limited precision of effect estimates. In particular, in subgroup analyses with small sample sizes, insufficient statistical power may exaggerate the ranking advantages of certain therapies. Therefore, SUCRA rankings are best viewed as exploratory findings; they do not establish definitive conclusions. Clinical decisions should therefore incorporate patient preferences, treatment accessibility, and safety.

In terms of clinical relevance, most combination therapies in this study were superior to antihistamine monotherapy in reducing the severity of pruritus and DLQI scores, with statistically significant differences. These findings suggest practical significance in improving patients' QoL. Notably, although autohemotherapy combined with moxibustion or BLP-C showed statistically significant differences in decreasing the severity of pruritus and reducing wheal counts, some results were affected by significant publication bias. This evidence of publication bias raises concerns about the reliability of these findings and suggests that clinical interpretation should be cautious.

In terms of safety and relapse rates, this study found that acupuncture combined with cupping therapy ranked highly in reducing adverse events and lowering the relapse rates. If subsequent studies confirm its long-term benefits, it may serve as an alternative or adjunctive treatment option for patients who are intolerant of antihistamines or have an inadequate response to them. Meta-regression analysis of the relapse rates did not identify a significant moderating effect of follow-up duration on the effect size, and between-study heterogeneity was very low. This finding enhances the robustness of the conclusions for this outcome and provides preliminary support for the long-term clinical management of CSU. However, the follow-up duration in the existing studies was predominantly short to medium term, averaging approximately 4.6 months. Longer-term maintenance therapy, long-term tolerability, and trends in relapse rates still need to be investigated.

### 4.5 Limitations

This study has several limitations. First, the search was restricted to Chinese and English publications, which may have resulted in the omission of relevant studies in other languages. Second, some interventions involved small sample sizes and lacked adequate randomization and blinding, compromising the overall quality of evidence. Moreover, all included studies were conducted in China and included only Chinese participants, limiting the generalizability of the results. Third, the network evidence for efficacy outcomes exhibited significant local inconsistency. This finding may be attributed to the predominance of two-arm trials and the relatively sparse network structure. Consequently, the statistical power for consistency tests was insufficient, and the *P*-values were easily influenced by random error. Furthermore, this NMA integrated diverse interventions (including acupuncture combined with autohemotherapy; moxibustion; acupuncture combined with moxibustion; autohemotherapy combined with moxibustion; the combination of acupuncture, BLP-C, and antihistamine; and acupuncture combined with cupping therapy) into a single analytical framework. Substantial heterogeneity existed across studies in intervention implementation, including acupoint selection, manipulation techniques, treatment duration, number of sessions, and practitioner dependence. Unlike pharmacological interventions with standardized dosing, the therapeutic effects of acupuncture depend largely on the specific context and the practitioner. Failure to adjust for the stimulation intensity of acupuncture interventions may compromise the robustness of the evidence and limit the interpretability of the comparative rankings.

Fourth, this study was not designed to test whether acupuncture outperforms placebo, but to compare the effectiveness of different acupuncture therapies in routine clinical practice. Therefore, we constructed this network to reflect real-world clinical decision-making, and sham acupuncture was not included as a comparator. However, previous high-quality RCTs have shown that acupuncture exhibits only modest differences in effects compared with sham acupuncture for certain conditions (139). Moreover, it is often difficult to distinguish sham acupuncture from placebo in clinical practice. The use of only drug comparators in this NMA may confound non-specific effects (including expectations from patients, treatment rituals, interactions between patients and practitioners, and the Hawthorne effect) with true specific effects. It is necessary to incorporate sham acupuncture-controlled studies. Therefore, the true efficacy of acupuncture can be assessed more precisely after excluding non-specific effects. Fifth, although this study included efficacy, the DLQI score, the severity of pruritus, and wheal counts as outcome measures, data for other outcomes—including the Chronic Urticaria Quality of Life Questionnaire (CU-Q2oL), UAS7, Angioedema Activity Score, and Urticaria Control Test—were insufficient for inclusion. This lack of data may limit the comprehensiveness of the evaluation of CSU. These limitations constrain the generalizability of our findings.

Sixth, the present analysis has certain limitations in the strategy used for grouping treatment nodes. To ensure network connectivity, we grouped different antihistamines into a single node according to the World Health Organization Anatomical Therapeutic Chemical classification for drugs of the same class. Although we provided the classification rationale for each original study item by item in the Supplementary Materials, and although this grouping strategy has been used in previous NMAs of similar scope, this crude grouping may mask differences in efficacy among specific interventions within the same class. Furthermore, this network included both comparisons of add-on effects and comparisons of substitution effects, which represent fundamentally different clinical decision-making contexts. When interpreting the overall efficacy rankings, readers should therefore carefully distinguish whether an intervention was evaluated as part of a combination regimen or as a standalone option. Future RCTs with larger sample sizes are needed to directly compare the relative effects of these treatment strategies in different patient subgroups and to further validate the conclusions of this exploratory analysis.

Seventh, although the population included in this analysis comprised some adolescents aged 13–17 years, none of the original studies reported outcome data by age subgroup, nor did any study use age as a randomization stratification factor for prespecified subgroup analyses. Therefore, the conclusions of this study mainly apply to adult patients with CSU. The relative effects of different treatment options in adolescent patients still need to be verified by RCTs specifically targeting this age group.

Future research should standardize acupuncture protocols and conduct subgroup analyses to evaluate the impact of acupoint selection, manipulation techniques, and operational methods on efficacy and safety. In addition, studies are needed to examine the effects of acupuncture on the local immune environment of the skin (such as the activity of mast cells and skin biopsy).

## 5 Conclusion

In conclusion, this study provides a relatively comprehensive overview of combined acupuncture therapies for CSU. Although some conclusions should be interpreted with caution, the overall evidence supports the feasibility of acupuncture, moxibustion, cupping therapy, and autohemotherapy as adjunctive or alternative treatment options. These findings highlight the potential value of these therapies for clinical application. However, owing to limitations and heterogeneity in existing studies, it is recommended that future investigations should prioritize larger-sample, multicenter RCTs to strengthen the evidence base. In addition, longer-term follow-up studies are required to assess sustained efficacy and relapse rates.

## Data Availability

The original contributions presented in the study are included in the article/Supplementary Material; further inquiries can be directed to the corresponding author/s.
