# Supplementary material for: Efficacy and safety of acupuncture therapy for chronic spontaneous urticaria: a network meta-analysis based on randomized controlled trials
Source: Front Med (Lausanne). 2026 Sep 30;13:1875737. doi: 10.3389/fmed.2026.1875737 (PMC13662173; doi:10.3389/fmed.2026.1875737)
Supplement: Supplementary file 1 [file Supplementaryfile1.docx]

Supplementary Materials

Content

Supplementary Table S1: The search strategy

Supplementary Table S2: Specific Implementation Methods for Each Intervention Measure and Efficacy

Supplementary Table S3: Assessment Scales for DLQI Score, Wheal Count, and Pruritus Severity

Supplementary Table S4: Basic Features of Included Studies

Supplementary Table S5: Pairwise Comparison Results of Varied Interventions on DLQI Score

Supplementary Table S6: Pairwise Comparison Results of Varied Interventions on Wheal Count

Supplementary Table S7: Pairwise Comparison Results of Varied Interventions on the Severity of Pruritus

Supplementary Table S8: Pairwise Comparison Results of Varied Interventions on IL-4 Concentration

Supplementary Table S9: Pairwise Comparison Results of Varied Interventions on IFN-γ Concentration

Supplementary Table S10: Pairwise Comparison Results of Varied Interventions on Relapse Rate

Supplementary Table S1 The search strategy

(Pubmed)

| Search number | Query | Results |
| --- | --- | --- |
| 1 | Acupuncture Therapy[MeSH Terms] | 30649 |
| 2 | Electroacupuncture[MeSH Terms] | 5313 |
| 3 | Moxibustion[MeSH Terms] | 2952 |
| 4 | auricular acupuncture[MeSH Terms] | 525 |
| 5 | "abdominal needle"[Title/Abstract] OR "ACET acupoint catgut embedding therapy"[Title/Abstract] OR "acupoint catgut embedding"[Title/Abstract] OR "acupoint catgut embedding therapy"[Title/Abstract] OR "acupoint injection"[Title/Abstract] OR "Acupotom*"[Title/Abstract] OR "acupuncture"[Title/Abstract] OR "Acupuncture Therapy"[Title/Abstract] OR "Acupuncture Treatmen*"[Title/Abstract] OR "auricular acupunctur*"[Title/Abstract] OR "auriculo acupuncture"[Title/Abstract] OR "auriculoacupuncture"[Title/Abstract] OR "auriculotherapy"[Title/Abstract] OR "catgut embedding"[Title/Abstract] OR "catgut implantation"[Title/Abstract] OR "Ear Acupunctur*"[Title/Abstract] OR "earlobe acupuncture"[Title/Abstract] OR "electric acupuncture"[Title/Abstract] OR "electrical acupoint stimulation"[Title/Abstract] OR "electrical acupuncture"[Title/Abstract] OR "electro acupuncture"[Title/Abstract] OR "Electroacupuncture"[Title/Abstract] OR "electrode acupuncture"[Title/Abstract] OR "electronic acupuncture"[Title/Abstract] OR "Moxabustion"[Title/Abstract] OR "Moxibustion"[Title/Abstract] OR "Pharmacoacupuncture Therapy"[Title/Abstract] OR "Pharmacoacupuncture Treatment"[Title/Abstract] OR "shonishin"[Title/Abstract] OR "warming needle moxibustion"[Title/Abstract] | 35342 |
| 6 | (#1 OR #2 OR #3 OR #4 OR #5) | 40803 |
| 7 | Chronic Urticaria[MeSH Terms] | 1583 |
| 8 | "Autoimmune Urticaria*"[Title/Abstract] OR "Chronic Autoimmune Urticaria*"[Title/Abstract] OR "chronic hives"[Title/Abstract] OR "Chronic Idiopathic Urticaria*"[Title/Abstract] OR "Chronic Spontaneous Urticaria*"[Title/Abstract] OR "Chronic Urticaria*"[Title/Abstract] OR "Idiopathic Chronic Urticaria*"[Title/Abstract] | 5296 |
| 9 | (#1 OR #2 OR #3 OR #4 OR #5) AND (#7 OR #8) | 80 |

(embase)

| Search number | Query | Results |
| --- | --- | --- |
| 1 | 'acupuncture'/exp | 60806 |
| 2 | 'electroacupuncture'/exp | 10132 |
| 3 | 'moxibustion'/exp | 5095 |
| 4 | 'auricular acupuncture'/exp | 954 |
| 5 | 'catgut embedding'/exp | 227 |
| 6 | 'abdominal needle':ti,ab,kw OR 'acet acupoint catgut embedding therapy':ti,ab,kw OR 'acupoint catgut embedding':ti,ab,kw OR 'acupoint catgut embedding therapy':ti,ab,kw OR 'acupoint injection':ti,ab,kw OR 'acupotom*':ti,ab,kw OR 'acupuncture':ti,ab,kw OR 'acupuncture therapy':ti,ab,kw OR 'acupuncture treatmen*':ti,ab,kw OR 'auricular acupunctur*':ti,ab,kw OR 'auriculo acupuncture':ti,ab,kw OR 'auriculoacupuncture':ti,ab,kw OR 'auriculotherapy':ti,ab,kw OR 'catgut embedding':ti,ab,kw OR 'catgut implantation':ti,ab,kw OR 'ear acupunctur*':ti,ab,kw OR 'earlobe acupuncture':ti,ab,kw OR 'electric acupuncture':ti,ab,kw OR 'electrical acupoint stimulation':ti,ab,kw OR 'electrical acupuncture':ti,ab,kw OR 'electro acupuncture':ti,ab,kw OR 'electroacupuncture':ti,ab,kw OR 'electrode acupuncture':ti,ab,kw OR 'electronic acupuncture':ti,ab,kw OR 'moxabustion':ti,ab,kw OR 'moxibustion':ti,ab,kw OR 'pharmacoacupuncture therapy':ti,ab,kw OR 'pharmacoacupuncture treatment':ti,ab,kw OR 'shonishin':ti,ab,kw OR 'warming needle moxibustion':ti,ab,kw | 48657 |
| 7 | 'chronic urticaria'/exp | 7918 |
| 8 | 'autoimmune urticaria*':ti,ab,kw OR 'chronic autoimmune urticaria*':ti,ab,kw OR 'chronic hives':ti,ab,kw OR 'chronic idiopathic urticaria*':ti,ab,kw OR 'chronic spontaneous urticaria*':ti,ab,kw OR 'chronic urticaria*':ti,ab,kw OR 'idiopathic chronic urticaria*':ti,ab,kw | 8727 |
| 9 | 'autoimmune urticaria*':ti,ab,kw OR 'chronic autoimmune urticaria*':ti,ab,kw OR 'chronic hives':ti,ab,kw OR 'chronic idiopathic urticaria*':ti,ab,kw OR 'chronic spontaneous urticaria*':ti,ab,kw OR 'chronic urticaria*':ti,ab,kw OR 'idiopathic chronic urticaria*':ti,ab,kw | 8727 |
| 10 | (#1 OR #2 OR #3 OR #4 OR #5 OR #6) AND (#7 OR #8) | 72 |

(Web of Science)

| Search number | Query | Results |
| --- | --- | --- |
| 1 | TS=((abdominal needle) OR (ACET acupoint catgut embedding therapy) OR (acupoint catgut embedding) OR (acupoint catgut embedding therapy) OR (acupoint injection) OR (Acupotom*) OR (acupuncture) OR (Acupuncture Therapy) OR (Acupuncture Treatmen*) OR (auricular acupunctur*) OR (auriculo acupuncture) OR (auriculoacupuncture) OR (auriculotherapy) OR (catgut embedding) OR (catgut implantation) OR (Ear Acupunctur*) OR (earlobe acupuncture) OR (electric acupuncture) OR (electrical acupoint stimulation) OR (electrical acupuncture) OR (electro acupuncture) OR (Electroacupuncture) OR (electrode acupuncture) OR (electronic acupuncture) OR (Moxabustion) OR (Moxibustion) OR (Pharmacoacupuncture Therapy) OR (Pharmacoacupuncture Treatment) OR (shonishin) OR (warming needle moxibustion)) | 39220 |
| 2 | TS=((Autoimmune Urticaria*) OR (Chronic Autoimmune Urticaria*) OR (chronic hives) OR (Chronic Idiopathic Urticaria*) OR (Chronic Spontaneous Urticaria*) OR (Chronic Urticaria*) OR (Idiopathic Chronic Urticaria*)) | 9177 |
| 3 | #2 AND #1 | 40 |

(Cochrane Library)

| Search number | Query | Results |
| --- | --- | --- |
| 1 | MeSH descriptor: [Acupuncture Therapy] explode all trees | 7142 |
| 2 | MeSH descriptor: [Electroacupuncture] explode all trees | 1175 |
| 3 | MeSH descriptor: [Moxibustion] explode all trees | 685 |
| 4 | MeSH descriptor: [Acupuncture, Ear] explode all trees | 266 |
| 5 | ('abdominal needle' OR 'ACET acupoint catgut embedding therapy' OR 'acupoint catgut embedding' OR 'acupoint catgut embedding therapy' OR 'acupoint injection' OR 'Acupotom*' OR 'acupuncture' OR 'Acupuncture Therapy' OR 'Acupuncture Treatmen*' OR 'auricular acupunctur*' OR 'auriculo acupuncture' OR 'auriculoacupuncture' OR 'auriculotherapy' OR 'catgut embedding' OR 'catgut implantation' OR 'Ear Acupunctur*' OR 'earlobe acupuncture' OR 'electric acupuncture' OR 'electrical acupoint stimulation' OR 'electrical acupuncture' OR 'electro acupuncture' OR 'Electroacupuncture' OR 'electrode acupuncture' OR 'electronic acupuncture' OR 'Moxabustion' OR 'Moxibustion' OR 'Pharmacoacupuncture Therapy' OR 'Pharmacoacupuncture Treatment' OR 'shonishin' OR 'warming needle moxibustion'):ti,ab,kw | 24651 |
| 6 | MeSH descriptor: [Chronic Urticaria] explode all trees | 129 |
| 7 | ('Autoimmune Urticaria*' OR 'Chronic Autoimmune Urticaria*' OR 'chronic hives' OR 'Chronic Idiopathic Urticaria*' OR 'Chronic Spontaneous Urticaria*' OR 'Chronic Urticaria*' OR 'Idiopathic Chronic Urticaria*'):ti,ab,kw | 1465 |
| 8 | (#1 OR #2 OR #3 OR #4 OR #5) AND (#6 OR #7) | 37 |

(SinoMed)

| Search number | Query | Results |
| --- | --- | --- |
| 1 | ( "针灸"[全部字段:智能] OR "温针灸"[全部字段:智能] OR "电针"[全部字段:智能] OR "艾灸"[全部字段:智能] OR "耳穴"[全部字段:智能] OR "穴位埋线"[全部字段:智能] OR "穴位注射"[全部字段:智能] OR "火针"[全部字段:智能] OR "头针"[全部字段:智能] OR "穴位贴敷"[全部字段:智能] OR "皮内针"[全部字段:智能] OR "微针"[全部字段:智能] OR "针刺"[全部字段:智能] OR "脐灸"[全部字段:智能] OR "经皮穴位电刺激"[全部字段:智能] OR "体针"[全部字段:智能] OR "头皮针"[全部字段:智能] OR "腕踝针激光针灸"[全部字段:智能]) AND "慢性荨麻疹"[全部字段:智能] | 811 |

(CNKI)

| Search number | Query | Results |
| --- | --- | --- |
| 1 | (主题：针灸 + 温针灸 + 电针 + 艾灸 + 耳穴 + 穴位埋线 + 穴位注射 + 火针 + 头针 + 穴位贴敷 + 皮内针 + 微针 + 针刺 + 脐灸 + 经皮穴位电刺激 + 体针 + 头皮针 + 腕踝针激光针灸)AND(主题：慢性荨麻疹) | 764 |

(WanFang)

| Search number | Query | Results |
| --- | --- | --- |
| 1 | (主题：针灸 OR 温针灸 OR 电针 OR 艾灸 OR 耳穴 OR 穴位埋线 OR 穴位注射 OR 火针 OR 头针 OR 穴位贴敷 OR 皮内针 OR 微针 OR 针刺 OR 脐灸 OR 经皮穴位电刺激 OR 体针 OR 头皮针 OR 腕踝针激光针灸)AND(主题：慢性荨麻疹) | 699 |

(VIP)

| Search number | Query | Results |
| --- | --- | --- |
| 1 | (主题：针灸 OR 温针灸 OR 电针 OR 艾灸 OR 耳穴 OR 穴位埋线 OR 穴位注射 OR 火针 OR 头针 OR 穴位贴敷 OR 皮内针 OR 微针 OR 针刺 OR 脐灸 OR 经皮穴位电刺激 OR 体针 OR 头皮针 OR 腕踝针激光针灸)AND(主题：慢性荨麻疹) | 696 |

Supplementary Table S2 Specific Implementation Methods for Each Intervention Measure and Efficacy

| No. | Author | Year | Intervention | | | Criterion of Effect |
| --- | --- | --- | --- | --- | --- | --- |
|  |  |  | C | T1 | T2 |  |
| 1 | YeHu | 2024 | Received treatment through acupuncture on the Governor Vessel, bloodletting, and cupping therapy. Specific procedures: ① During the visual examination of the patient, start from the Baihui acupoint and observe any possible cyanosis, dark congestion, or stagnant blood vessels. After routinely disinfecting the surrounding skin, use the left hand to gently lift the skin in this area to create a stable working environment for the needle insertion, while the right hand holds the needle and inserts it vertically into the skin quickly and gently; ② Prepare fire cups of the appropriate size in advance. After puncturing, quickly attach the fire cup to the location of the blood stasis and keep it in place for 5 minutes; ③ After observing that the blood stasis has been completely drained, remove the fire cup and wipe the patient's skin clean with sterile gauze. Treat continuously for 3 months. | On the basis of the control group, "Xin Baliao" acupuncture treatment was additionally administered. During the acupuncture operation of "New Baoli", the patient was in a prone position. position. Select a 0.25 mm × 75 mm disposable sterile acupuncture needle (Suzhou Dongbang Medical Equipment Co., Ltd.; approved by Suzhou 20192200828), 1 finger below the coccyx and 1 finger lateral to it, starting the first needle. This needle should be inserted close to the bone to ensure the accuracy and effectiveness of the acupuncture. Then, at a 45° angle (33 mm away from the first needle), apply the second needle, followed by the third and fourth needles, and so on. The specific acupoints are Shang0, Xia0, Zhong0 and Xia0 (each pair on the left and right sides), totaling 8 needles in the inverted "eight" shape pattern. The needles are retained for 30 minutes. During the actual acupuncture process, the insertion depth should be 70 to 100 mm according to the patient's body type. With At that time, within 30 minutes after the acupuncture procedure, the patient is required to remain mentally relaxed and focus their attention on the acupuncture site. The treatment will last for 3 months. |  | After 3 months of treatment, the clinical therapeutic effect of the chronic urticaria of the blood deficiency and dryness syndrome can be classified into four levels: complete recovery, marked improvement, effective, and ineffective. Complete recovery: With the treatment, the wheals completely disappear and the pruritus disappears completely; Marked improvement: The wheals basically disappear and the pruritus symptoms basically disappear, and the frequency and duration of the attacks of the patients significantly decrease; Effective: The wheals decrease and the pruritus alleviates, the frequency and duration of the attacks of the patients slightly decrease; Ineffective: After the treatment, the clinical symptoms have not been relieved, and some symptoms even show a tendency to worsen. The total effective rate = recovery rate + marked improvement rate + effective rate Efficacy。 |
| 2 | YuShi | 2023 | Medication: Cetirizine Hydrochloride Tablets. Take once daily, 10mg per dose. One course lasts for 10 days. Administer for 3 consecutive courses, with an interval of 2-3 days between each course. | The acupuncture and cupping therapy was applied for intervention. The acupoints selected were: Quchi (both sides), Hegu (both sides), Zhongwan, Tianshu, Guanyuan, Xuehai (both sides), Zusanli (both sides), Yinlingquan (both sides), Sanyingjiao (both sides). First, the bilateral Zusanli and Sanyingjiao acupoints (30-60 mm) were treated with straight needles, with 60 seconds of twisting and reinforcing therapy. Then, Zhongwan (20-40 mm) was selected, and 60 seconds of low-amplitude high-frequency twisting and reinforcing therapy was performed. The bilateral Quchi (30-50 mm) were treated with 60 seconds of twisting and reinforcing therapy. Hegu, Tianshu, and Guanyuan were treated with conventional acupuncture therapy. Then, the patient was required to lie on their stomach, exposing the back skin. The Dazhui and Geshu (both sides) acupoints were selected. After routine disinfection, the above acupoints were punctured one by one using a blood collection pen. Each acupoint was punctured 5 times, with the depth of penetration being the needle tip penetrating the skin, and then cupping was performed immediately and maintained for 10 minutes. The bleeding volume at each point was approximately 2 ml as the standard. The two methods were used in combination. Frequency: once every other day, for a total of 12 times, forming one course of treatment. |  | According to Urticaria activity score over 7d (UAS7), the efficacy index was calculated. Efficacy index = [(total UAS7 score before therapy - total UAS7 score after therapy)÷total UAS7 score before therapy] ×100%. Cured: the efficacy index was ≥90%. Effective: the efficacy index was ≥20% but <90%. Ineffective: the efficacy index was <20%. Total response rate = (1 - the number of ineffective cases/total cases) ×100% |
| 3 | YingjieWang | 2013 | Mizolastine 10mg, taken orally once a day; one course consists of 14 days of treatment. | Acupoint: Shenque (CV 8). Method: Placed the ignited thunder-fire moxa stick into holes of a moxibustion box (approximately 2-3 cm away from the bottom of the box) and pinned the stick; placed the moxibustion box over the patient’s navel to make the moxa stick aim at Shenque (CV 8) for 15 min until the skin became red and a heat sensation in the deep tissue. The moxibustion was conducted once a day |  | The therapeutic efficacy was assessed according to the symptom scorereducing index (SSRI).SSRI= (Pre-treatment score – Post-treatment score) ÷ Pre-treatment total score × 100%.Recovery: SSRI ≥90%.Marked effect: SSRI between 60% and 89%.Improvement: SSRI between 20% and 59%.Failure: SSRI <20% or became worse.Total effective rate = (Recovery cases + Marked effect cases) ÷ Number of cases in the group × 100%. |
| 4 | JinyeGan | 2018 | Loratadine Tablets, 10 mg. Take 30 minutes after meals, once a day. Take for 8 consecutive days. | (1) Acupoints: Geshu,Feishu,Pishu.(2) Procedure: Instruct the patient to lie on their stomach. Disinfect the diaphragm acupoint, lung acupoint, and spleen acupoint routinely. Then, use a disposable injection needle to puncture each acupoint one by one. Each acupoint should be pricked 3 to 5 times. After slightly squeezing out 2 drops of blood, select a medium-sized fire cupping jar to fix it. Keep the cup on for about 10 minutes. The amount of bleeding should be 3 to 4 mL. After removing the cup, press the puncture hole to stop the bleeding and wipe it with a sterile cotton ball to prevent infection. The bloodletting treatment at the back acupoints is performed once every other day, and a total of 4 treatments are given. |  | Refer to the efficacy evaluation criteria for urticaria in the "Standards for Diagnosis and Treatment of TCM Syndromes and Conditions". Cure: When the wheals have completely disappeared, the clinical symptoms have vanished, and there are no more recurrences; Improvement: When the wheals have decreased by 30% or when they recur with an extended interval after disappearance, and the itching and other symptoms have alleviated; Unhealed: When there is no significant improvement in the wheals and itching, or when the reduction is less than 30%. The total effective rate = Cure rate + Improvement rate. |
| 5 | Yongnan Jia | 2020 | Administer Epinastine tablets, 10 mg orally, 1 tablet each time, once daily. A 3-week period constitutes one treatment course. The treatment will be carried out for a total of 3 courses. | Apply the line-skin therapy combined with autologous blood therapy. 1) Line-skin therapy. Points selected: bilateral Feishu, Xinshu, Geshu, Ganshu, Sanjiaoshu. Instruct the patient to lie on their back, expose the back, the operator first locates the points, then marks and positions them, routinely disinfect with iodophor, wear a mask and sterile gloves, open the embedding package, according to the thickness of the muscle at the embedding site, cut the collagen protein line soaked in 75% ethanol into 1-2 cm segments, put them in the embedding box, rinse with normal saline, then use sterile forceps to insert a disposable 9-gauge sterile embedding needle, slowly insert the embedding needle into the selected points, when the patient feels acid, numbness, distension, or pain, gently and slowly push the needle core, push the collagen protein line into the muscle layer under the selected points, then withdraw the needle, disinfect with iodophor. After all the points are embedded, perform cupping therapy at the embedding point area, leave the cup for 5 minutes before removing it, disinfect again with iodophor, then apply a bottle mouth patch to the embedding area, remove the bottle mouth patch after 2 hours. 2) Autologous blood therapy. Points selected： bilateral Zusanli. Instruct the patient to lie in a supine position, fully expose the outer sides of both lower legs, locate the points and make marks, then follow the venipuncture operation method, use a 5 mL disposable syringe to draw 4 mL of the patient's venous blood, routinely disinfect the acupoints, then quickly insert the needle tip of the syringe into the points, when the patient feels acid, numbness, distension, or pain at the local area, if there is no return blood, inject the venous blood into Zusanli on both sides, 2 mL each, withdraw the needle, apply a bottle mouth patch. Treat once a week, 3 weeks as one course, treat for 3 courses in total. | Treatment consisted of tapping with plum-blossom needles followed by cupping, combined with autologous blood therapy. 1) Tapping with plum-blossom needles followed by cupping. Acupoints selected: bilateral Lung Shu (BL13), Heart Shu (BL15), Diaphragm Shu (BL18), Liver Shu (BL19), and Triple Energizer Shu (BL22). Instruct the patient to lie prone, exposing the Bladder Meridian on the back. Disinfect the acupoint areas with povidone-iodine cotton swabs. Use the plum-blossom needle to perform tapping at the acupoints until slight bleeding occurs or the patient reaches their tolerance limit. Subsequently, apply fire cupping to the tapped areas and leave the cups in place for 5 minutes. Afterward, clean the area and perform routine disinfection. 2) Autologous blood therapy. The procedure, treatment course, and precautions are the same as those for the cupping group. | The Symptom Score Reduction Index (SSRI) is calculated based on the total clinical symptom scores before and after treatment to assess clinical efficacy. SSRI = (Total score before treatment – Total score after treatment) / Total score before treatment × 100%. Recovery: SSRI ≥ 90%. Marked improvement: 60% ≤ SSRI < 90%. Improvement: 30% ≤ SSRI < 60%. No improvement: SSRI < 30%. Overall response rate (%) = (Number of cases with complete recovery + Number of cases with marked improvement + Number of cases with improvement) / Total number of cases × 100% |
| 6 | Jun Zhao | 2018 | Blood-letting and Cupping Therapy: ① Acupoint selection: Dazhui, both Feishu, Fengmen, both Xuehai, and both Quchi; ② Disinfect the skin as usual, then use a three-edged needle to puncture each acupoint several times until blood is drawn; ③ Apply a small glass cupping jar to each acupoint, leaving it in place for 10 minutes; the bloodletting volume should be approximately 5 mL; ④ Remove the cups, wipe away the blood, and disinfect the area. Perform the treatment 3 times per week; one course of treatment consists of 2 weeks, and a total of 4 consecutive courses are recommended. | Based on the control group, autologous blood acupoint injection therapy was administered as follows: ① Disinfect the skin over the median cubital vein; use a 5 mL syringe to draw 3 mL of venous blood; ② Insert the needle into both Zusanli acupoints and perform a lifting-and-thrusting technique until the patient feels a sensation of soreness and distension; ③ Aspirate to confirm no blood remains, then rapidly inject the blood into the acupoints; ④ Remove the needle and apply pressure with an alcohol swab to disinfect the site; ⑤ Administer treatment three times a week; one course of treatment consists of two weeks; continue treatment for four consecutive courses. |  | Efficacy was evaluated according to the *Standards for the Efficacy of Traditional Chinese Medicine Diseases and Syndromes and the Selection of Formulas*: ① Cure: The patient experienced no urticaria episodes during the follow-up period; clinical symptoms and signs disappeared; the skin scratch test was negative; and the score reduction index was ≥90%; ② Improvement: More than 60% of the patient’s wheals resolved; itching was significantly alleviated; the frequency of episodes decreased significantly; and the score reduction index was ≥60%; ③ Effective: More than 30% of the wheals have resolved, itching has improved to some extent, and the score reduction index is ≥20%; ④ Ineffective: No change in the patient’s clinical symptoms and signs, and the score reduction index is <20%. Overall efficacy rate = (Number of cured cases + Number of cases showing improvement + Number of effective cases) / Total number of cases × 100%. |
| 7 | Quan Li | 2015 | Cetirizine hydrochloride 10 mg/tablet, taken orally once daily for 4 consecutive weeks. | Using the blood-letting cupping method, select the following acupoints: Dazhui (GV14), both Feishu (BL13), both Quchi (LI11), and both Xuehai (SP10). After locating and marking the points, perform routine local disinfection. Use a sterilized three-edged needle to make several rapid punctures at the above acupoints; it is best to draw blood. Immediately apply small fire cups, leaving them in place for 10 minutes. The ideal blood loss is 3–5 mL. After removing the cups, wipe away the blood, clear any stasis, and disinfect the area. Treatment is administered every other day, with a 7-day course of treatment, for a total of 4 consecutive weeks. |  | Based on the 1995 MILOR study in Europe, scoring is conducted using a four-point scale ranging from 0 to 3. ① Number of wheals: no wheals, 0 points; 1–10 wheals, 1 point; 10–20 wheals, 2 points; more than 20 wheals, 3 points. ② Size of wheals: no wheals, 0 points; wheal diameter < 1.0 cm, 1 point; diameter 1.0–2.5 cm, 2 points; diameter >2.5 cm, 3 points. ③ Severity of itching: no itching, 0 points; mild itching that does not interfere with sleep, 1 point; moderate itching that interferes with sleep but is tolerable, 2 points; severe itching that prevents sleep and is intolerable, 3 points. ④ Frequency of urticaria: no urticaria, 0 points; occurs once daily, 1 point; 2–5 times, 2 points; >5 times, 3 points. ⑤ Duration of urticaria: no urticaria, 0 points; urticaria lasting <4 hours, 1 point; lasting 4–12 hours, 2 points; lasting >12 hours, 3 points. ⑥ Skin scratch test: no skin lesions, 0 points; slow wheal formation, 1 point; rapid wheal formation, 2 points; very rapid wheal formation, 3 points. Total Score Reduction Index = (Pre-treatment Total Score – Post-treatment Total Score) / Pre-treatment Total Score × 100%. Complete Remission: Total Score Reduction Index ≥ 90%; Marked Improvement: Reduction Index 70%–89%; Improvement: Reduction Index 30%–69%; No Effect: Reduction Index < 30%. Overall Response Rate = Complete Remission Rate + Marked Improvement Rate + Improvement Rate. |
| 8 | Hong Gao | 2009 | Medication: Levocetirizine hydrochloride tablets, 5 mg per dose, taken orally after dinner Stepwise dosing regimen: Weeks 1–2: Once daily; Weeks 3–4: Once every other day; Weeks 5–6: Once every 3 days; The full course of treatment is 6 weeks. | Acupoint: Shendao (below the spinous process of the 5th thoracic vertebra) Needles: .4mm × 75mm thick stainless steel needles Procedure: The patient tilts their head downward and rounds their chest. Insert the needle subcutaneously at a 30° angle, penetrating horizontally along the midline of the spine until the needle passes through the skin. Leave only 0.5 cm of the needle shaft protruding from the skin. Leave the needle in place for 4 hours each session; Frequency: Once daily, 5 times per week, followed by a 2-day rest period; Course of Treatment: 2 consecutive weeks (1 course only). |  | 1. Itching: 0 points = no itching; 1 point = mild, not bothersome; 2 points = moderate, tolerable; 3 points = severe, intolerable 2. Urticaria size: 0 points = no urticaria; 1 point = ≤1.5 cm; 2 points = 1.5–2.5 cm; 3 points = ≥2.5 cm 3. Number of wheals: 0 points = no wheals; 1 point = 1–6 wheals; 2 points = 7–12 wheals; 3 points = >12 wheals The sum of these three scores constitutes the total symptom score; accompanying laboratory test: fasting serum IgE (chemiluminescence assay) Observation time points: Before treatment; 2 weeks, 6 weeks, and 12 weeks after treatment |
| 9 | Hong Gao | 2009 | Lizethilizine Hydrochloride Tablets 5mg, to be taken orally after dinner each day; Course of treatment: 6 weeks. | 1. Acupoint selection: Shen Dao (below the spinous process of the 5th thoracic vertebra) 2. Needle: A 75mm long, 0.4mm thick stainless steel needle 3. Procedure: The patient lowers their head and presses their chest. Insert the needle at a 30° angle under the skin, penetrate along the midline of the spine under the skin, leaving only 0.5cm of the needle body outside the body; keep the needle in place for 4 hours each time. 4. Frequency and treatment course: Treat 5 days a week, rest for 2 days, and continue for 2 weeks. 5. Observation points: Evaluate the therapeutic effect and IgE after 2 weeks (short-term), 6 weeks (mid-term), and 12 weeks (long-term) of treatment. |  | Itching: 0 - No itching; 1 - Mild and not irritating; 2 - Moderate and tolerable; 3 - Severe and unbearable Size of wheals: 0 - No wheals; 1 - Less than 1.5 cm; 2 - 1.5 - 2.5 cm; 3 - More than 2.5 cm Number of wheals: 0 - No wheals; 1 - 1 - 6; 2 - 7 - 12; 3 - More than 12 2. SSRI score decline index determination SSRI = (Total score before treatment - Total score after treatment) / Total score before treatment × 100% Remission: SSRI ≥ 90% Marked effect: 60% ≤ SSRI < 90% Effective: 20% ≤ SSRI < 60% No effect: SSRI < 20% Remission rate = (Remission + Marked effect) / Total number of cases × 100% Comprehensive detection: Serum IgE levels at 2/6/12 weeks of treatment |
| 10 | Yuying Tang | 2015 | 2 mg of cyproheptadine, 5 mg of prednisone, and 0.2 g of vitamin C, taken orally three times daily; one course of treatment lasts 15 days. | 1. Solution preparation: Draw 2 mL of the patient’s own venous blood and mix it with 2 mL of Angelica sinensis injection; 2. Acupoint selection and dosage allocation (bilateral): 0.5 mL per side at Fengchi, 1 mL per side at Quchi, and 0.5 mL per side at Xuehai; 3. Procedure: Routinely disinfect the acupoints; after inserting the needle and obtaining "qi" (a sensation of soreness and distension), slowly inject the solution; 4. Frequency and Course of Treatment: Administer once every 5 days; one course consists of 15 days. |  | Cured: No itching or urticaria-like erythema on the skin; no recurrence after treatment; Marked Improvement: Only 1–5 small wheals and mild itching; Improvement: Reduction in the number of wheals and erythema, with alleviated itching; No Improvement: No improvement in symptoms. Overall response rate = (Number of cases classified as "Cured" + "Marked Improvement") / Total number of cases × 100%. |
| 11 | Zhongxun Wang | 2021 | Cetirizine Hydrochloride Tablets orally, 1 tablet daily, 4 consecutive weeks of treatment. | Body acupoints: Fengchi (GB20), Zhongwan (CV12), Tianshu (ST25), Guanyuan (CV4) - "Abdominal Four Needles", Quchi (LI11), Xuehai (SP10), Zusanli (ST36), Sanyinjiao (SP6). Auricular acupoints: Heart (Xin), Lung (Fei), Shenmen. Procedure: 1. First, seated position - needle Fengchi, twist-rotate for 5 seconds after deqi, then withdraw. 2. Change to supine position - needle remaining body points + auricular points. Retain needles for 30 minutes. 3. Frequency: Every other day, 3 times per week, total of 12 treatments (4-week course). |  | 1. Core SSRI efficacy criteria (referenced from "Guiding Principles for Clinical Research of New Chinese Medicines"). Symptom Score Reduce Index SSRI = (Pre-treatment score - Post-treatment score) / Pre-treatment score × 100%. Cured: SSRI ≥ 90%. Markedly effective: 60% ≤ SSRI < 90%. Ineffective: SSRI < 60%. Total effective rate = (Cured + Markedly effective) ÷ Total cases × 100%. 2. Associated scoring scales: 1. UAS Urticaria Activity Score: Pruritus and wheal number, 0-3 points each. 2. DLQI Dermatology Life Quality Index (0-30 points). 3. HAMA Hamilton Anxiety Rating Scale - assesses anxiety severity. |
| 12 | Qingjuan Guo | 2024 | Epinastine Hydrochloride Capsules (Chongqing Yaoyou Pharmaceutical Co., Ltd., batch No. 13502630; 6 capsules, 10 mg/capsule), 10 mg per dose, once daily. 30 days per course, total of 2 courses. Procedure: ① During visual inspection of the patient, start inspection from Dazhui (GV14) point. Observe for possible purple-blue stasis or dark stagnant collaterals. After routine disinfection of the surrounding skin, gently stretch the skin with the left hand to create a stable working surface for needling. Hold the needle with the right hand and perform rapid, light perpendicular skin punctures. ② Prepare appropriately sized fire cups in advance. After puncturing, rapidly apply cups to the bleeding stasis sites and retain for 5 minutes. ③ After observing that stasis blood is exhausted, remove cups and apply sterile gauze. Combined with Du-moxibustion therapy and autohemotherapy. | Body acupoints: Fengchi (GB20), Zhongwan (CV12), Tianshu (ST25), Guanyuan (CV4) - "Abdominal Four Needles", Quchi (LI11), Xuehai (SP10), Zusanli (ST36), Sanyinjiao (SP6). Auricular acupoints: Heart (Xin), Lung (Fei), Shenmen. Procedure: 1. First, seated position - needle Fengchi, twist-rotate for 5 seconds after deqi, then withdraw. 2. Change to supine position - needle remaining body points + auricular points. Retain needles for 30 minutes. 3. Frequency: Every other day, 3 times per week, total of 12 treatments (4-week course). |  | Efficacy evaluated according to "Guiding Principles for Clinical Research of New Chinese Medicines". Efficacy index = (Pre-treatment UAS score - Post-treatment UAS score) / Pre-treatment UAS score × 100%. Markedly effective: Efficacy index > 60%. Effective: Efficacy index 30%-60%. Ineffective: Efficacy index < 30%. Total effective rate = (Markedly effective + Effective) cases / Total cases × 100%. ③ Recurrence rate: Follow-up period of 6 months. Telephone follow-up every 2 months after trial completion. Recurrence is defined as ≥2 episodes of wheals or pruritus within 1 week. Recurrence rate = Recurrence cases after recovery / Total effective cases × 100%. |
| 13 | Meiling Qiu | 2020 | Mizolastine Tablets, 10 mg per dose, orally 3 times daily. Course: 15 days. | 1. Yangxue Xiaofeng Decoction (Blood-Nourishing Wind-Eliminating Decoction) Formula: Chuanxiong (Rhizoma Chuanxiong) 8 g, Cangzhu (Rhizoma Atractylodis) 10 g, Danggui (Radix Angelicae Sinensis) 10 g, Longgu (Os Draconis) 30 g, Huangqi (Radix Astragali) 15 g, Zhizi (Fructus Gardeniae) 10 g, Heshouwu (Radix Polygoni Multiflori) 15 g. Administration: 1 dose daily, add water and decoct to 200 mL, divide into two warm doses (morning and evening), 15 consecutive days. 2. Acupuncture: Acupoints: Xuehai (SP10), Quchi (LI11), Zusanli (ST36), Sanyinjiao (SP6), Geshu (BL17). Manipulation: Reducing method. Retain needles for 10 minutes. Frequency: Once daily. Course: 15 days. |  | Markedly effective: All wheals, pruritus and other symptoms completely resolved, no recurrence. Effective: Pruritus and wheal symptoms significantly alleviated, minimal impact on daily life. Ineffective: No improvement or worsening of symptoms. Total effective rate = (Markedly effective + Effective) cases ÷ Total cases × 100%. |
| 14 | Bingling Zhang | 2013 | Loratadine Tablets (Claritin) 10 mg, orally once daily. Course: 4 weeks. | 1. Modified Guizhi Decoction (Cinnamon Twig Decoction): Wuzhimaotao (Radix Fici Hirtae) 15 g, Guizhi (Ramulus Cinnamomi) 12 g, Mudanpi (Cortex Moutan) 12 g, Baishao (Radix Paeoniae Alba) 12 g, Danggui (Radix Angelicae Sinensis) 10 g, Jingjie (Herba Schizonepetae) 10 g, Fangfeng (Radix Saposhnikoviae) 10 g, Shengjiang (Rhizoma Zingiberis Recens) 10 g, Zhigancao (Radix Glycyrrhizae Praeparata) 6 g. Administration: 1 dose daily, decoct in water to obtain 400 mL, divide into two warm doses (morning and evening). 2. Acupuncture protocol: Acupoints: Fengchi (GB20), Quchi (LI11), Hegu (LI4), Xuehai (SP10), Zusanli (ST36), Sanyinjiao (SP6). Frequency: Twice weekly. Unified course: 4 weeks. Dietary restrictions same as control group. |  | Clinical recovery: No wheals, no erythema, no pruritus; no recurrence at 3-month follow-up. Markedly effective: Wheals reduced by ≥70%, pruritus significantly alleviated, recurrence intervals prolonged. Effective: Wheals reduced by ≥30%, pruritus somewhat alleviated. Ineffective: No significant improvement in symptoms and signs. Total effective rate = (Recovery + Markedly effective + Effective) ÷ Total cases × 100%. Associated observations: Follow-up at 1 month after treatment completion for recurrence statistics, record adverse reactions such as dry mouth. |
| 15 | Ziwen Dong | 2019 | Loratadine Tablets 10 mg orally, once daily. Course: 4 weeks. | 1. Modified Guizhi Decoction Formula: Wuzhimaotao (Radix Fici Hirtae) 15 g, Guizhi (Ramulus Cinnamomi) 12 g, Mudanpi (Cortex Moutan) 12 g, Baishao (Radix Paeoniae Alba) 12 g, Fangfeng (Radix Saposhnikoviae) 10 g, Shengjiang (Rhizoma Zingiberis Recens) 10 g, Danggui (Radix Angelicae Sinensis) 10 g, Jingjie (Herba Schizonepetae) 10 g, Zhigancao (Radix Glycyrrhizae Praeparata) 6 g. Decoction for oral administration. 2. Acupuncture acupoints and frequency: Acupoints: Sanyinjiao (SP6), Xuehai (SP10), Zusanli (ST36), Quchi (LI11), Hegu (LI4), Fengchi (GB20). Frequency: Twice weekly. Unified course: 4 weeks. |  | Cured: Pruritus, erythema, and wheals completely disappeared, no recurrence for 3 months. Markedly effective: Wheal episodes reduced by ≥75%, pruritus and erythema significantly relieved. Ineffective: No improvement, recurrent episodes or worsening. Total effective rate = (Cured + Markedly effective) ÷ Total cases × 100%. Observation indicators: Adverse reactions after treatment, recurrence rate at 1-month follow-up after treatment completion. |
| 16 | Xiaoling Ye | 2023 | Loratadine Tablets (Chengdu Hengrui Pharmaceutical Co., Ltd., National Medicine Approval No. H20030112). Tapering regimen: Month 1: 10 mg once daily; Month 2: 10 mg every other day; Month 3: 10 mg every 2 days. 3 consecutive months of treatment. | Start with large cups for moxibustion and tuina along the Governor Vessel and Foot Taiyang Bladder Meridian. Then use small cups for point-pressing and scraping techniques. Alternate six techniques: rolling, pushing, plucking, scraping, moxibustion, and point-pressing. Apply point technique at acupoints: Quchi (LI11), Hegu (LI4), Zusanli (ST36), Xuehai (SP10), Sanyinjiao (SP6), Pishu (BL20), Geshu (BL17). |  | Self-made urticaria symptom score sheet for clinical symptom scoring. Symptoms include number of wheals, size, and pruritus severity. Wheal number scoring: 0 points = no wheals; 1 point = ≤10 wheals; 2 points = 10-25 wheals; 3 points = >25 wheals. Wheal size scoring: 0 points = no wheals; 1 point = diameter <1.5 cm; 2 points = diameter 1.5-2.5 cm; 3 points = diameter >2.5 cm. Pruritus severity scoring: 0 points = no pruritus; 1 point = mild pruritus; 2 points = moderate pruritus; 3 points = severe pruritus. |
| 17 | Yanjun Wang | 2022 | Levocetirizine Hydrochloride Oral Solution (Chongqing Huapont Pharmaceutical Co., Ltd., National Medicine Approval No. H20061289, 10 mL). 10 mL per dose, orally once daily, 4 weeks of treatment. | Pricking blood cupping at Dazhui (GV14), Feishu (BL13), Geshu (BL17), Pishu (BL20), once every other day. Auricular acupoints: Lung (Fei), Kidney (Shen), Shenmen, Heart (Xin), Endocrine (Neifenmi), Subcortex (Pizhixia), Wind Stream (Fengxi). Procedure: Disinfect auricle. Apply disposable auricular acupoint patches to selected points. Select unilateral auricular points per treatment, alternate between both ears. Instruct patient to self-press daily: 1-2 minutes per point, until local warmth and redness. Press 3-5 times per day. Change patches every other day. |  | "Guiding Principles for Clinical Research of New Chinese Medicines (Trial)", efficacy criteria based on patient UAS7 scores. Symptom Score Reduce Index (SSRI) = [(Pre-treatment UAS7 total score - Post-treatment UAS7 total score) ÷ Pre-treatment UAS7 total score] × 100%. Cured: SSRI ≥ 90%. Markedly effective: 60% ≤ SSRI < 90%. Effective: 20% ≤ SSRI < 60%. Ineffective: SSRI < 20%. Total effective rate = (Cured + Markedly effective + Effective) cases / Total cases × 100%. |
| 18 | Yanjun Wang | 2021 | Levocetirizine Hydrochloride Oral Solution (Chongqing Huapont Pharmaceutical Co., Ltd., National Medicine Approval No. H20061289, 10 mL). 10 mL per dose, orally once daily, 4 weeks of treatment. | Combined acupuncture and cupping therapy. Acupuncture first, then cupping. Both acupuncture and cupping every other day. Both groups: 4 consecutive weeks. Acupuncture acupoints: Zhongwan (CV12), Xiawan (CV10), Guanyuan (CV4), bilateral Quchi (LI11), bilateral Shousanli (LI10), bilateral Hegu (LI4), bilateral Xuehai (SP10), bilateral Zusanli (ST36), bilateral Sanyinjiao (SP6). Routine needling, even reinforcing-reducing method. Retain needles for 25 minutes after deqi. Cupping acupoints: Shenque (CV8), Dazhui (GV14), bilateral Feishu (BL13), bilateral Geshu (BL17), bilateral Pishu (BL20). Procedure: Shenque - flash cupping until local skin flushing, retain 5 minutes. Dazhui, Feishu, Geshu, Pishu - retain cups 10-15 minutes. |  | UAS7 used to score wheals and pruritus in both groups. Evaluate once daily for 7 consecutive days. Calculate total score over 7 days. Use UAS7 score as symptom score, calculate Symptom Score Reduce Index (SSRI). SSRI = (Pre-treatment UAS7 score - Post-treatment UAS7 score) / Pre-treatment UAS7 score × 100%. Cured: SSRI ≥ 90%. Markedly effective: 60% ≤ SSRI < 90%. Effective: 20% ≤ SSRI < 60%. Ineffective: SSRI < 20%. Total effective rate = (Cured + Markedly effective + Effective) cases / Total cases × 100%. |
| 19 | Zhuo Huang | 2020 | Ebastine Tablets (Industrias Farmacéuticas Almirall, Spain, National Medicine Approval No. H20140855). Oral administration, once daily, 10 mg per dose. 2 months per course, 1 consecutive course of treatment. | First, autohemotherapy: Local skin disinfection with iodophor. Draw 5 mL venous blood with 5 mL disposable sterile syringe. Gently agitate. Inject directly into gluteal muscle without additives. Frequency: Once weekly, alternating bilateral gluteal injection. Course: 2 months. Second, herbal formula: Shenggancao (Radix Glycyrrhizae) 3 g, Pianjianghuang (Rhizoma Wenyujin Concisum) 10 g, Baijiangcan (Bombyx Batryticatus) 10 g, Jingchantui (Periostracum Cicadae) 10 g, Jiuqinhuang (Radix Scutellariae, wine-fried) 15 g, Zijingpi (Cortex Kadsurae) 15 g, Mudanpi (Cortex Moutan) 15 g, Fangfeng (Radix Saposhnikoviae) 15 g, Sangbaipi (Cortex Mori) 15 g, Shengdihuang (Radix Rehmanniae) 30 g, Chishaoyao (Radix Paeoniae Rubra) 30 g, Shengcishi (Magnetitum) 30 g. Add 500 mL water, soak for 30 minutes. Bring to rolling boil over high heat, then simmer for 30 minutes. Extract 300 mL decoction. Add appropriate water again, bring to boil then simmer for 20 minutes. Extract 150 mL decoction. Mix both decoctions thoroughly. Divide into 3 warm doses, all taken 30 minutes after meals. 1 dose daily. |  | Referenced from "Diagnostic and Efficacy Standards for TCM Diseases" issued by the State Administration of Traditional Chinese Medicine. ① Wheal score: 0 points = no wheals; 1 point = ≤20 wheals; 2 points = 20-50 wheals; 3 points = >50 wheals or large confluent patches. Sum scores for 7 days before treatment and 7 days after 1 course of treatment. ② Pruritus score: 0 points = no pruritus; 1 point = mild pruritus, does not interfere with daily life; 2 points = moderate pruritus, interferes with life but does not affect sleep and daily activities; 3 points = severe pruritus, seriously affects sleep and daily activities. Scoring method same as wheal score. ③ Total score = Wheal score + Pruritus score (0-6 points daily; higher score = more severe symptoms) |
| 20 | Baozhen Lin | 2020 | Cetirizine Hydrochloride Tablets (UCB Pharma, Belgium/Switzerland, Import Drug Registration Standard Approval No. Jx20020065, H20100740). Oral administration, 10 mg/tablet, once daily, 1 tablet per dose. | Xiaofeng San (Wind-Eliminating Powder) combined with fire needle therapy. ① Xiaofeng San composition: Huangqi (Radix Astragali) 15 g, Shengdihuang (Radix Rehmanniae) 15 g, Baizhu (Rhizoma Atractylodis Macrocephalae) 9 g, Baishao (Radix Paeoniae Alba) 9 g, Guizhi (Ramulus Cinnamomi) 9 g, Jingjie (Herba Schizonepetae) 9 g, Danggui (Radix Angelicae Sinensis) 9 g, Kushen (Radix Sophorae Flavescentis) 9 g, Beichaihu (Radix Bupleuri) 9 g, Gancao (Radix Glycyrrhizae) 6 g, Chantui (Periostracum Cicadae) 6 g, Fangfeng (Radix Saposhnikoviae) 10 g. Use formula granules from 999 Pharmaceutical Co., Ltd. Divide into 2 oral doses (morning and evening). For severe wheal itching: Add 3 doses of this formula, soak in 250 mL of 53-degree Baijiu (10-year), apply supernatant topically. ② General acupuncture and fire needle therapy: Routine disinfection of lesion area before treatment. Acupuncture acupoints: Hegu (LI4), Chize (LU5), Quchi (LI11), Zusanli (ST36), Taiyuan (LU9). Retain for 30 minutes. After needle withdrawal, gather 10 pieces of 4-cun acupuncture needles, use alcohol lamp for rapid fire needling along both Hand Taiyin Lung and Foot Yangming Stomach meridians, back and forth 3 times. Frequency: Twice weekly. Course: 4 weeks. |  | Cured: All wheals completely disappeared after treatment, no pruritus symptoms. Markedly effective: Wheals significantly reduced by >3/4 during episodes, and skin lesion pruritus significantly improved. Effective: Wheals reduced by 1/3-3/4, and skin lesion pruritus somewhat improved. Ineffective: Wheals reduced by <1/3, and skin lesion itching not improved. |
| 21 | Min Li | 2014 | Loratadine Tablets (Jiangsu Yabang Aipusen Pharmaceutical Co., Ltd., brand name: Claritin), 10 mg per dose, once daily. 4 weeks per course. Avoid spicy, shrimp, and crab foods during treatment. | Oral modified Xiaofeng San combined with acupuncture therapy, 4 weeks per course. Avoid spicy, shrimp, and crab foods during treatment. 1. Modified Xiaofeng San prescription: Jingjie (Herba Schizonepetae) 10 g, Danggui (Radix Angelicae Sinensis) 6 g, Niubangzi (Fructus Arctii) 10 g, Shengdi (Radix Rehmanniae) 15 g, Humaren (Semen Sesami Nigrum) 10 g, Fangfeng (Radix Saposhnikoviae) 10 g, Chantui (Periostracum Cicadae) 6 g, Zhimu (Rhizoma Anemarrhenae) 12 g, Shengshigao (Gypsum Fibrosum, decocted first) 10 g, Kushen (Radix Sophorae Flavescentis) 10 g, Cangzhu (Rhizoma Atractylodis) 6 g, Qinjiao (Radix Gentianae Macrophyllae) 15 g, Cijili (Fructus Tribuli) 15 g, Gancao (Radix Glycyrrhizae) 6 g. 1 dose daily, decoct to 400 mL, divide into two warm doses (morning and evening). 2. Acupuncture protocol: Needle Sanyinjiao (SP6), Quchi (LI11), Xuehai (SP10), Hegu (LI4), Zusanli (ST36). Frequency: Twice weekly. |  | Referenced from chronic urticaria efficacy criteria in "Guiding Principles for Clinical Research of New Chinese Medicines". Clinical recovery: No wheals, no erythema, no pruritus. Markedly effective: Wheal episodes reduced by 70%, pruritus significantly alleviated. Effective: Wheal episodes reduced by >30%, pruritus symptoms alleviated. Ineffective: No significant improvement in symptoms and signs. Total effective rate includes clinical recovery, markedly effective, and effective. |
| 22 | Libin Gu | 2019 | Loratadine Tablets 10 mg orally, once daily. 4 weeks per course, total of 2 courses (8 weeks total). | 1. Acupoints: Bilateral Jianyu (LI15), Yangxi (LI5). 2. Needle: 0.30 mm × 40 mm disposable filiform needle. 3. Yin-hiding-yang manipulation technique: First insert needle to 1 cun depth. Slow press and tight lift, perform six yin counts until deqi. Withdraw needle to 0.5 cun depth. Tight press and slow lift, perform nine yang counts until deqi. Retain needle for 20 minutes, manipulate once every 10 minutes. 4. Course: 4 weeks per course, 8 consecutive weeks (2 courses). |  | Efficacy assessed by Symptom Score Reduce Index (SSRI). SSRI = (Pre-treatment total score - Post-treatment total score) ÷ Pre-treatment score × 100%. Cured: SSRI ≥ 90%. Markedly effective: 60% ≤ SSRI ≤ 89%. Improved: 20% ≤ SSRI ≤ 59%. Ineffective: SSRI < 20%. Total effective rate = (Cured + Markedly effective + Improved) cases / Total cases × 100%. Associated laboratory markers: Serum IL-4, IgE, IFN-γ levels before and after treatment. |
| 23 | Xinghua Lin | 2014 | Ebastine tablets combined with cimetidine orally, standard daily dose. | 1. Treatment group (Jin's Three-Needle Abdominal Three Needles + Shenque Cupping). Primary points: Abdominal Three Needles (Zhongwan CV12, Tianshu ST25, Guanyuan CV4), Shenque (CV8). Supplementary points: Quchi (LI11), Xuehai (SP10), Zusanli (ST36). Procedure: ① Shenque cupping: Size 3 fire cup, retain 5 minutes, remove and repeat 3 times; total cupping time: 15 minutes. ② Acupuncture: Routine disinfection, 1.5 cun filiform needles, retain for 30 minutes after deqi. Warm needle moxibustion with moxa wool at Zhongwan, Guanyuan, and Zusanli. Course: 10 treatments per course, 3 days rest between courses. Course 1: once daily; Courses 2-3: every other day. | Oral modified Guizhi Decoction herbal decoction, 1 dose daily, divided into morning and evening warm doses. Course same as treatment group. | Cured: Wheals and pruritus completely disappeared, no recurrence at follow-up. Improved: Frequency and area of wheal and pruritus episodes significantly reduced. Unimproved: No improvement or worsening. Effective rate = (Cured + Improved) cases / Total cases × 100%. |
| 24 | Hongjun Xiao | 2018 | Cetirizine Hydrochloride Tablets 10 mg, orally once daily. Loratadine Tablets 10 mg, orally once daily. | 1. Kangmin Decoction (Anti-Allergy Decoction) base formula: Jinyinhua (Flos Lonicerae) 10 g, Qiancao (Radix Rubiae) 10 g, Mohanlian (Herba Ecliptae) 10 g, Zicao (Radix Arnebiae) 10 g, Baijili (Fructus Tribuli) 15 g, Fangfeng (Radix Saposhnikoviae) 10 g, Jingjie (Herba Schizonepetae) 10 g, Huangqi (Radix Astragali) 24 g, Baishao (Radix Paeoniae Alba) 15 g, Danggui (Radix Angelicae Sinensis) 10 g, Shudihuang (Radix Rehmanniae Praeparata) 24 g, Gancao (Radix Glycyrrhizae) 6 g. Pattern modifications: Marked pruritus - add Xuchangqing (Radix Cynanchi Paniculati) 10 g, Baixianpi (Cortex Dictamni) 10 g. Wind-cold pattern - add Guizhi (Ramulus Cinnamomi) 9 g, Mahuang (Herba Ephedrae) 6 g. Wind-heat pattern - add Juhua (Flos Chrysanthemi) 15 g, Bohe (Herba Menthae) 6 g; replace Shudihuang with Shengdihuang. Spleen-stomach deficiency-cold - add Ganjiang (Rhizoma Zingiberis) 6 g, Guizhi (Ramulus Cinnamomi) 9 g, Baizhu (Rhizoma Atractylodis Macrocephalae) 10 g. Administration: 1 dose daily, decoct in water, divide into 2 warm doses (morning and evening). 2. Acupoint autohemotherapy: Acupoints - Bilateral Quchi (LI11), Xuehai (SP10), Zusanli (ST36). Procedure: Aseptically draw 5 mL cubital venous blood. Inject 0.5-1 mL per acupoint. Frequency: Twice weekly. |  | Markedly effective: Wheals and pruritus completely disappeared, no recurrence at 6-month follow-up. Effective: Wheals and pruritus significantly improved, occasional episodes within 6 months. Ineffective: No improvement or worsening, frequent recurrences within 6 months. Total effective rate = (Markedly effective + Effective) ÷ Total cases × 100%. Symptom score: 4 items (wheal size, number, duration, pruritus), 0-3 points each. Lower total score = milder condition. Follow-up period: 6 months after treatment completion for recurrence statistics. |
| 25 | Aiying Wei | 2019 | Cetirizine Hydrochloride Tablets 10 mg, orally once daily. Loratadine Tablets 10 mg, orally once daily. Course: 1 month. | 1. Kangmin Decoction base formula: Danggui (Radix Angelicae Sinensis) 10 g, Jingjie (Herba Schizonepetae) 10 g, Zicao (Radix Arnebiae) 10 g, Hanliancao (Herba Ecliptae) 10 g, Qiancao (Radix Rubiae) 10 g, Jinyinhua (Flos Lonicerae) 10 g, Fangfeng (Radix Saposhnikoviae) 10 g, Baishao (Radix Paeoniae Alba) 15 g, Baijili (Fructus Tribuli) 15 g, Shengdi (Radix Rehmanniae) 24 g, Shengqi (Radix Astragali) 24 g, Gancao (Radix Glycyrrhizae) 6 g. Pattern modifications: Severe pruritus - add Xuchangqing, Baixianpi. Wind-cold pattern - add Mahuang, Guizhi. Wind-heat pattern - add Bohe, Juhua. Spleen-stomach deficiency-cold - add Baizhu, Guizhi, Ganjiang. Administration: 1 dose daily, decoct in water, divide into 2 warm doses (morning and evening), 1 consecutive month. 2. Acupoint autohemotherapy: Acupoints - Bilateral Quchi (LI11), Zusanli (ST36), Xuehai (SP10). Procedure: Draw 5 mL cubital venous blood. Inject 0.5-1 mL per acupoint. Frequency: Twice weekly. Course: 1 month. |  | Markedly effective: All symptoms including wheals and pruritus completely disappeared. Effective: Wheals and pruritus symptoms significantly alleviated. Ineffective: No improvement in symptoms. Total effective rate = (Markedly effective + Effective) cases ÷ Total cases × 100%. Associated observations: Adverse reactions during treatment, recurrence rate at 1 month after treatment completion. |
| 26 | Bianying Ren | 2020 | 1. Blood collection: 5 mL syringe draws 2 mL of cubital venous blood. 2. Alternating acupoints: Odd days - Pishu (BL20), Xuehai (SP10); Even days - Geshu (BL17), Zusanli (ST36). 3. Procedure: Acupoint disinfection. Deqi sensation, aspirate to confirm no blood return, then inject 1 mL autologous blood per point. Apply dry cotton swab to needle hole. 4. Frequency and course: Once daily, 10 treatments per course. | Autohemotherapy procedure, acupoints, and course identical to control group, with additional thunder-fire moxibustion at Shenque (CV8). 1. Apparatus: Standard thunder-fire moxa stick. 2. Position: Supine, suspended moxibustion method. 3. Standard: Heat penetrating abdominal cavity, skin flushing as optimal. 4. Duration and frequency: 20 minutes per session, once daily, synchronized with autohemotherapy. Course: 10 treatments per course. |  | Markedly effective: Main symptoms such as wheals and pruritus significantly alleviated. Effective: Symptoms somewhat relieved. Ineffective: No improvement or worsening. Total effective rate = (Markedly effective + Effective) / Total cases × 100%. |
| 27 | Xihua Kui | 2021 | Loratadine Tablets (Yangtze River Pharmaceutical, 10 mg/tablet), 10 mg per dose, once daily. Total course: 24 days. | 1. Acupuncture: Acupoints - Quchi (LI11), Sishencong (EX-HN1), Huaroumen (ST24), Zusanli (ST36), Wailing (ST26), Xuehai (SP10). Routine disinfection, even reinforcing-reducing method. Retain needles for 30 minutes. 2. Shenque cupping: Large glass fire cup - flash cupping 4-5 times, then retain for 10 minutes. 3. Back moving cupping + pricking bloodletting: Apply lubricating oil to back. Move cups along Bladder Meridian and Governor Vessel until skin congestion. Plum blossom needle tapping along Governor Vessel and Bladder Meridian. Flash cupping for 10 minutes. Additional three-edged needle pricking at ear apex, Quchi, Weizhong, etc. Cupping bloodletting. Wipe blood clean after completion. |  | Markedly effective: Clinical symptoms and signs basically disappeared, wheals completely resolved. Improved: Symptoms significantly relieved, wheal resolution >30%. Ineffective: No improvement before and after treatment. Total effective rate = (Markedly effective + Improved) cases ÷ Total cases × 100%. |
| 28 | Yan Liu | 2012 | Cetirizine Hydrochloride Tablets (Minda, National Medicine Approval No. H19990321, Nanjing Jinling Pharmacy Co., Ltd.), 10 mg per dose, once daily. Qufeng Zhiyang Oral Liquid (National Medicine Approval No. B20020371, Sichuan Taihua Pharmaceutical Co., Ltd.), 10 mL per dose, 3 times daily. | Self-composed Yiqi Gubiao Decoction (Qi-Tonifying Exterior-Securing Decoction) orally combined with acupoint thread embedding therapy. 30 days per course. 1. Yiqi Gubiao Decoction formula and administration: Dangshen (Radix Codonopsis) 20 g, Fuling (Poria) 30 g, Jingjie (Herba Schizonepetae) 12 g, Fangfeng (Radix Saposhnikoviae) 12 g, Chanyi (Periostracum Cicadae) 12 g, Shudihuang (Radix Rehmanniae Praeparata) 20 g, Heshouwu (Radix Polygoni Multiflori) 20 g, Danggui (Radix Angelicae Sinensis) 15 g, Baixianpi (Cortex Dictamni) 30 g, Difuzi (Fructus Kochiae) 15 g, Chenpi (Pericarpium Citri Reticulatae) 25 g, Baijili (Fructus Tribuli) 15 g, Chuanxiong (Rhizoma Chuanxiong) 12 g, Gancao (Radix Glycyrrhizae) 9 g. Modifications: Spleen deficiency predominant - add Huangqi (Radix Astragali) 30 g, Chaobaizhu (Rhizoma Atractylodis Macrocephalae, fried) 15 g. Yang deficiency predominant - add Guizhi (Ramulus Cinnamomi) 12 g, Ganjiang (Rhizoma Zingiberis) 15 g. 1 dose daily, decoct to obtain 400 mL, divide into morning and evening doses. 2. Acupoint thread embedding: Acupoints - Bilateral Zusanli (ST36), Xuehai (SP10), Geshu (BL17), Sanyinjiao (SP6). Modifications: Wheals predominantly on head and face - add Fengchi (GB20). On lower back - add Feishu (BL13). On abdomen - add Tianshu (ST25). Predominantly upper limbs - add Waiguan (SJ5). Predominantly lower limbs - add Weizhong (BL40). Procedure: Routine local disinfection. Use size 2 catgut, load into size 12 puncture needle. Rapid subcutaneous insertion. Back-Shu points: oblique insertion. Limb points: perpendicular insertion. Withdraw needle while retracting stylet, embed 2 cm catgut. Apply dressing after needle withdrawal. Frequency: Once every 10 days. |  | Efficacy assessed according to "Diagnostic and Efficacy Standards for TCM Diseases". Cured: Wheals resolved, clinical symptoms disappeared, no recurrence. Markedly effective: Wheals reduced by ≥70% or recurrence interval significantly prolonged, pruritus significantly alleviated. Effective: Wheals reduced by ≥30% or recurrence interval prolonged compared to pre-treatment, pruritus alleviated. Ineffective: No significant improvement in wheals and pruritus. |
| 29 | Juan Wang | 2019 | Acupoints: Bilateral Quchi (LI11), Xuehai (SP10), Yinlingquan (SP9), Sanyinjiao (SP6). Procedure: Supine position. Aseptically draw 4 mL venous blood. Inject 1 mL per acupoint. Deqi sensation, aspirate to confirm no blood return before injecting. Apply dry cotton swab to needle hole. Frequency and course: Once every 3 days, total of 10 treatments per course. | Autohemotherapy procedure, acupoints, frequency, and course same as Group A. Plum blossom needle tapping procedure, frequency, and course same as Group B. Both therapies performed simultaneously. | Procedure: Prone position. Disinfect back Bladder Meridian. Focus tapping on Pishu (BL20), Weishu (BL21). Tapping frequency: 70-90 times/minute. 15-20 minutes per session, until slight skin oozing. Frequency and course: Every other day, 1 consecutive month. | 1. Number of wheals: 0 points = none; 1 point = 1-10 wheals; 2 points = 11-20 wheals; 3 points = >20 wheals. 2. Wheal size: 0 points = none; 1 point = <1 cm; 2 points = 1-2.5 cm; 3 points = >2.5 cm. 3. Pruritus severity: 0 points = none; 1 point = mild; 2 points = moderate; 3 points = severe. 4. Episode duration: 0 points = no wheals; 1 point = <4 hours; 2 points = 4-12 hours; 3 points = >12 hours. Associated observation indicators: CD4⁺, CD8⁺, CD4⁺/CD8⁺ immune T cell subsets. Follow-up at 6 months after treatment completion for recurrence rate statistics. |
| 30 | Xiaoqi Liao | 2016 | Desloratadine Tablets 5 mg, once daily orally. Add Setastine Hydrochloride Tablets 1 mg at bedtime for patients with numerous wheals and severe pruritus. Course: 4 weeks. | Acupoints: Bilateral Quchi (LI11). Procedure: Routine skin disinfection. Rapid pricking with three-edged needle, 5-10 pricks. May express blood or apply small fire cup suction. Approximately 1 mL bleeding per site. Frequency: Every other day. Course: 4 weeks. |  | Efficacy assessed by Symptom Score Reduce Index (SSRI). SSRI = (Pre-treatment total score - Post-treatment total score) ÷ Pre-treatment total score × 100%. Cured: SSRI ≥ 90%. Markedly effective: 60% ≤ SSRI ≤ 89%. Improved: 20% ≤ SSRI ≤ 59%. Ineffective: SSRI < 20%. Total effective rate = (Cured + Markedly effective + Improved) cases / Total cases. Four-grade individual symptom scoring (0-3 points each): 1. Pruritus: 0 = no itching; 1 = mild, does not affect sleep; 2 = moderate, awakens ≤2 times at night; 3 = severe, difficulty falling asleep. 2. Wheal number: 0 = no wheals; 1 = ≤6 wheals; 2 = 7-20 wheals; 3 = >20 wheals. 3. Number of episodes: 0 = none; 1 = once daily; 2 = 2-3 times daily; 3 = >3 times daily. 4. Duration: 0 = no wheals; 1 = ≤4 hours; 2 = 4-12 hours; 3 = >12 hours. |
| 31 | Furong Liu | 2022 | Levocetirizine Hydrochloride Tablets, 10 mg per dose, once daily orally. 4 consecutive weeks per course. | 1. Qufeng Xiaxun Decoction (Wind-Dispelling Urticaria-Eliminating Decoction) oral administration. Base formula: Gegen (Radix Puerariae) 30 g, Sangbaipi (Cortex Mori) 15 g, Chantui (Periostracum Cicadae) 6 g, Baizhi (Radix Angelicae Dahuricae) 10 g, Baixianpi (Cortex Dictamni) 20 g, Zhizi (Fructus Gardeniae) 10 g, Digupi (Cortex Lycii) 10 g, Kushen (Radix Sophorae Flavescentis) 10 g, Huangqi (Radix Astragali) 30 g, Danggui (Radix Angelicae Sinensis) 6 g, Shengdahuang (Radix et Rhizoma Rhei) 3 g. Administration: 1 dose daily, decoct to obtain 200 mL, divide into morning and evening doses, 4 consecutive weeks. 2. Acupoint thread embedding protocol (4 weeks per course, once weekly). Thread length: 1 cm. Rotating acupoint schedule: Weeks 1 & 3: Bilateral Quchi (LI11), Zusanli (ST36), Xuehai (SP10). Weeks 2 & 4: Bilateral Feishu (BL13), Geshu (BL17), Ganshu (BL18). Frequency: Once weekly, total of 4 treatments. |  | Referenced from "Guiding Principles for Clinical Research of New Chinese Medicines", assessed by comprehensive symptom score reduction rate: Cured: Comprehensive score reduction rate ≥ 95%. Markedly effective: 60% ≤ Comprehensive score reduction rate < 95%. Effective: 30% ≤ Comprehensive score reduction rate < 60%. Ineffective: Score reduction rate < 30%. Comprehensive score includes pruritus, wheal size, wheal number, and duration. Associated with 0-10 point NRS pruritus numeric rating scale. Total effective rate = (Cured + Markedly effective + Effective) cases / Total cases × 100%. |
| 32 | Xurui Wang | 2022 | Loratadine Tablets (10 mg/tablet, Henan Tianfang Pharmaceutical, H20067066), orally once daily at fixed time. Light diet throughout treatment, avoid seafood and stimulating foods, avoid wind and cold stimulation, avoid hot water stimulation. | 1. Herbal oral administration: Formula, administration method, and dietary restrictions same as herbal group. 2. Three-Wind Points thread embedding. Acupoints (bilateral): Fengmen (BL12), Fengshi (GB31), Fengshiqian (Dong's extra point, Anterior Fengshi), Quchi (LI11), Xuehai (SP10). Materials: 3-0 absorbable surgical suture, No. 7 gauge flat-tip embedding needle. Thread length: 1.5 cm. Procedure: Strict skin disinfection, supine position. Insert needle 2-3 cm until deqi. Push thread into subcutaneous tissue. Withdraw needle, apply pressure to needle eye, cover with dressing. Keep acupoints dry for 24 hours. Frequency: Every 2 weeks. Total: 2 treatments within 4 weeks. |  | Danggui Yinzi (Angelica Decoction) - Granule form. Formula: Danggui (Radix Angelicae Sinensis) 20 g, Chuanxiong (Rhizoma Chuanxiong) 15 g, Baishao (Radix Paeoniae Alba) 15 g, Shengdihuang (Radix Rehmanniae) 15 g, Jingjie (Herba Schizonepetae) 12 g, Fangfeng (Radix Saposhnikoviae) 15 g, Shengqi (Radix Astragali) 20 g, Jixueteng (Caulis Spatholobi) 15 g, Cijili (Fructus Tribuli) 9 g, Zhiheshouwu (Radix Polygoni Multiflori Praeparata) 10 g, Zhigancao (Radix Glycyrrhizae Praeparata) 6 g. Administration: Dissolve in 100 mL warm water, 3 times daily, 4 consecutive weeks. Dietary restrictions same as western medicine group. |
| 33 | Junying Jiao | 2017 | Cetirizine Hydrochloride Tablets 10 mg, once daily orally. Course: 4 weeks. | Acupoint catgut embedding combined with self-blood therapy. 1. Acupoint catgut embedding: Acupoints - Quchi (LI11), Xuehai (SP10), Zusanli (ST36), Sanyinjiao (SP6). Materials: No. 0 catgut, No. 9 lumbar puncture needle. Procedure: Routine disinfection. Insert needle to appropriate depth, push catgut while withdrawing needle. Apply sterile dressing. Frequency: Once every 7 days. 2. Self-blood therapy: Draw 4 mL venous blood, inject into bilateral Zusanli, 2 mL per side. Frequency: Once every 7 days. Course: 4 weeks. |  | Efficacy criteria: Cured: All symptoms disappeared, no recurrence. Markedly effective: Symptoms significantly improved. Effective: Symptoms improved. Ineffective: No improvement. Total effective rate = (Cured + Markedly effective + Effective) / Total cases × 100%. Laboratory indicators: Serum total IgE, eosinophil count before and after treatment. |
| 34 | Weijie Chen | 2018 | Loratadine 10 mg, once daily orally. Course: 4 weeks. | Fire needle therapy. Acupoints: Quchi (LI11), Xuehai (SP10), Fengshi (GB31), Zusanli (ST36). Procedure: Routine disinfection. Heat needle tip with alcohol lamp until red-hot. Rapidly puncture acupoints. Each point: 3-5 pricks. Depth: 0.5-1 cm. Frequency: Twice weekly. Course: 4 weeks. |  | Efficacy assessed by SSRI. Cured: SSRI ≥ 90%. Markedly effective: 60% ≤ SSRI < 90%. Effective: 30% ≤ SSRI < 60%. Ineffective: SSRI < 30%. Total effective rate = (Cured + Markedly effective + Effective) / Total × 100%. Safety: Observe adverse reactions such as pain, blisters, scarring at treatment sites. |
| 35 | Zhongfang Lin | 2014 | Mizolastine Sustained Release Tablets 10 mg, once daily orally. Course: 4 weeks. | Acupoint injection with Vitamin B12. Acupoints: Quchi (LI11), Xuehai (SP10), Zusanli (ST36), Sanyinjiao (SP6). Procedure: Draw 1 mL Vitamin B12 injection (0.5 mg). Inject 0.25 mL per acupoint. Frequency: Once every other day. Course: 4 weeks. |  | Efficacy criteria: Cured: Wheals completely subsided, pruritus disappeared, no recurrence. Markedly effective: Wheals reduced by ≥60%, pruritus significantly alleviated. Effective: Wheals reduced by 20%-60%, pruritus alleviated. Ineffective: Wheals reduced by <20%, no improvement in pruritus. Total effective rate = (Cured + Markedly effective + Effective) / Total cases × 100%. |
| 36 | Qiong wu | 2017 | Desloratadine Citrate Tablets 5 mg, once daily orally. Course: 28 days. | Back-shu point tapping and cupping. Acupoints: Feishu (BL13), Xinshu (BL15), Geshu (BL17), Ganshu (BL18), Pishu (BL20), Shenshu (BL23). Procedure: Plum blossom needle tapping along Bladder Meridian on both sides of the back. Then apply moving cupping. Retain cups at key points for 5-10 minutes. Frequency: Twice weekly. Course: 4 weeks. |  | UAS7 scoring: Daily assessment of wheals and pruritus for 7 consecutive days. Total score of 7 days. Efficacy: Cured: SSRI ≥ 90%. Markedly effective: 60% ≤ SSRI < 90%. Effective: 20% ≤ SSRI < 60%. Ineffective: SSRI < 20%. Total effective rate = (Cured + Markedly effective + Effective) / Total × 100%. Recurrence rate: Follow up 3 months after treatment. |
| 37 | Jieru He | 2009 | Fexofenadine Hydrochloride Tablets 120 mg, once daily orally. Course: 4 weeks. | Auricular point pressing combined with body acupuncture. Auricular points: Lung, Liver, Spleen, Kidney, Endocrine, Subcortex, Shenmen, Wind Stream. Body points: Quchi (LI11), Xuehai (SP10), Zusanli (ST36), Sanyinjiao (SP6). Procedure: Auricular point pressing with vaccaria seeds. Press 3-5 times daily, 1-2 minutes each time. Change every 3 days. Body acupuncture: retain needles 30 minutes. Frequency: Every other day. Course: 4 weeks. |  | Efficacy assessment: Cured: All symptoms disappeared, skin returned to normal, no recurrence within 1 month. Markedly effective: Symptoms significantly improved, wheals reduced by ≥70%. Effective: Symptoms improved, wheals reduced by 30%-70%. Ineffective: No significant improvement, wheals reduced by <30%. Total effective rate = (Cured + Markedly effective + Effective) / Total × 100%. |
| 38 | Wenna Shu | 2023 | Levocetirizine Hydrochloride Tablets 5 mg, once daily at bedtime. Course: 4 weeks. | Moxibustion at Shenque (CV8). Procedure: Indirect moxibustion with ginger or salt. 3-5 moxa cones per treatment. Frequency: Once daily. Course: 4 weeks. |  | Efficacy criteria (according to "Guidelines for Clinical Research of New Chinese Medicines"): Cured: Wheals completely disappeared, pruritus disappeared, no recurrence for 1 month. Markedly effective: Wheals reduced by ≥70%, pruritus significantly alleviated. Effective: Wheals reduced by 30%-70%, pruritus alleviated. Ineffective: Wheals reduced by <30%, no improvement in pruritus. Total effective rate = (Cured + Markedly effective + Effective) / Total cases × 100%. |
| 39 | Guihong Ye | 2018 | Cetirizine Hydrochloride Tablets 10 mg, once daily orally. Course: 4 weeks. | Cupping at Shenque (CV8). Procedure: Flash cupping method. 5-7 flash suctions per treatment, until local skin flushing. Frequency: Once daily. Course: 4 weeks. |  | Symptom scoring: Wheal number, wheal size, pruritus severity, each 0-3 points. Total score: 0-9 points. Efficacy: Cured: Symptoms completely disappeared, score reduction ≥90%. Markedly effective: Score reduction 60%-89%. Effective: Score reduction 30%-59%. Ineffective: Score reduction <30%. Total effective rate = (Cured + Markedly effective + Effective) / Total × 100%. |
| 40 | Hairong Wang | 2018 | Loratadine Tablets 10 mg, once daily orally. Course: 30 days. | Acupoint injection with placental tissue fluid. Acupoints: Quchi (LI11), Xuehai (SP10), Zusanli (ST36). Procedure: Draw 4 mL placental tissue fluid. Inject 1 mL per acupoint. Frequency: Once every other day. Course: 30 days. |  | Efficacy assessment: Cured: Wheals completely subsided, pruritus disappeared, no recurrence. Markedly effective: Wheals reduced by ≥70%, pruritus significantly improved. Effective: Wheals reduced by 30%-70%, pruritus improved. Ineffective: Wheals reduced by <30%, no improvement in pruritus. Total effective rate = (Cured + Markedly effective + Effective) / Total × 100%. |
| 41 | Zhenqin Zhou | 2015 | Ebastine Tablets 10 mg, once daily orally. Course: 4 weeks. | Wax therapy at Shenque (CV8). Procedure: Apply melted paraffin wax (45-50°C) to umbilical area. Thickness: 1-2 cm. Cover with plastic film and towel. Duration: 30 minutes. Frequency: Once daily. Course: 4 weeks. |  | Efficacy criteria: Cured: All symptoms and signs disappeared, no recurrence within 6 months. Markedly effective: Symptoms and signs basically disappeared, occasional recurrence but mild and self-resolving. Effective: Symptoms and signs improved, but still need medication. Ineffective: No significant improvement. Total effective rate = (Cured + Markedly effective + Effective) / Total × 100%. |
| 42 | Ning Su | 2011 | Chlorpheniramine Maleate Tablets 4 mg, 3 times daily orally. Course: 4 weeks. | Herbal fumigation and washing. Formula: Kushen (Radix Sophorae Flavescentis) 30 g, Baixianpi (Cortex Dictamni) 30 g, Difuzi (Fructus Kochiae) 30 g, Shechuangzi (Fructus Cnidii) 30 g, Fangfeng (Radix Saposhnikoviae) 20 g, Jingjie (Herba Schizonepetae) 20 g, Bohe (Herba Menthae) 15 g. Procedure: Decoct herbs, fumigate affected areas while hot, then wash when warm. Duration: 20-30 minutes. Frequency: Twice daily. Course: 4 weeks. |  | Efficacy assessment: Cured: Skin lesions completely subsided, pruritus disappeared. Markedly effective: Skin lesions subsided by ≥60%, pruritus significantly alleviated. Effective: Skin lesions subsided by 20%-60%, pruritus alleviated. Ineffective: Skin lesions subsided by <20%, no improvement in pruritus. Total effective rate = (Cured + Markedly effective + Effective) / Total cases × 100%. |
| 43 | Zhijun Zhang | 2024 | Cyproheptadine Hydrochloride Tablets 2 mg, 3 times daily orally. Course: 4 weeks. | Acupoint application therapy. Acupoints: Shenque (CV8), Guanyuan (CV4), Qihai (CV6). Formula: Dingchuan powder mixed with ginger juice. Procedure: Apply to acupoints, retain for 4-6 hours. Frequency: Once every other day. Course: 4 weeks. |  | Efficacy criteria: Cured: Wheals completely disappeared, pruritus vanished, no recurrence for 3 months. Markedly effective: Wheals reduced by ≥70%, pruritus significantly relieved. Effective: Wheals reduced by 30%-70%, pruritus relieved. Ineffective: Wheals reduced by <30%, no improvement in pruritus. Total effective rate = (Cured + Markedly effective + Effective) / Total × 100%. |
| 44 | Jin Zhao | 2019 | Promethazine Hydrochloride Tablets 25 mg, 2-3 times daily orally. Course: 4 weeks. | Bloodletting at ear apex points. Procedure: Disinfect ear apex. Prick with three-edged needle. Squeeze out 5-10 drops of blood. Frequency: Once every other day. Course: 4 weeks. |  | Symptom score: Pruritus (0-3 points), wheal number (0-3 points), wheal size (0-3 points), wheal duration (0-3 points). Total: 0-12 points. Efficacy: Cured: Score reduction ≥90%. Markedly effective: Score reduction 60%-89%. Effective: Score reduction 30%-59%. Ineffective: Score reduction <30%. Total effective rate = (Cured + Markedly effective + Effective) / Total × 100%. |
| 45 | Mengyao Yang | 2021 | Diphenhydramine Hydrochloride Tablets 25 mg, 3 times daily orally. Course: 4 weeks. | Gua Sha (scraping therapy) along Bladder Meridian on the back. Procedure: Apply scraping oil. Scrape along Bladder Meridian from top to bottom. Until petechiae appear. Frequency: Once every 3-5 days. Course: 4 weeks. |  | Efficacy assessment according to TCM syndrome efficacy standards: Cured: TCM symptoms and signs disappeared or basically disappeared, syndrome score reduction ≥95%. Markedly effective: TCM symptoms and signs significantly improved, syndrome score reduction ≥70%. Effective: TCM symptoms and signs improved, syndrome score reduction ≥30%. Ineffective: TCM symptoms and signs not significantly improved, syndrome score reduction <30%. Total effective rate = (Cured + Markedly effective + Effective) / Total × 100%. |
| 46 | Yihui Zhang | 2017 | Terfenadine Tablets 60 mg, twice daily orally. Course: 4 weeks. | Cupping therapy along Governor Vessel and Bladder Meridian. Procedure: Moving cupping along the back. Retain cups at Dazhui (GV14), Feishu (BL13), Geshu (BL17), Pishu (BL20), Shenshu (BL23). Retain 10-15 minutes. Frequency: Twice weekly. Course: 4 weeks. |  | Efficacy criteria: Cured: Wheals completely resolved, no pruritus, no recurrence for 2 months. Markedly effective: Wheals reduced by ≥70%, pruritus significantly relieved. Effective: Wheals reduced by 30%-70%, pruritus relieved. Ineffective: Wheals reduced by <30%, no improvement in pruritus. Total effective rate = (Cured + Markedly effective + Effective) / Total cases × 100%. |
| 47 | Fei Liu | 2018 | Astemizole Tablets 10 mg, once daily orally. Course: 4 weeks. | Moxibustion with ginger at Zusanli (ST36). Procedure: Place ginger slice on acupoint. Place moxa cone on ginger. Ignite moxa. 3-5 cones per point. Frequency: Once every other day. Course: 4 weeks. |  | Quality of life assessment: DLQI (Dermatology Life Quality Index) score. Efficacy: Cured: DLQI score reduction ≥90%. Markedly effective: DLQI score reduction 60%-89%. Effective: DLQI score reduction 30%-59%. Ineffective: DLQI score reduction <30%. Total effective rate = (Cured + Markedly effective + Effective) / Total × 100%. |
| 48 | Keping Tan | 2016 | Ketotifen Fumarate Tablets 1 mg, twice daily orally. Course: 4 weeks. | Acupressure at Fengchi (GB20), Hegu (LI4), Quchi (LI11), Xuehai (SP10), Zusanli (ST36). Procedure: Press and knead each acupoint for 1-2 minutes. Until soreness and distension sensation. Frequency: 3 times daily. Course: 4 weeks. |  | Recurrence rate follow-up: Follow up 6 months after treatment. Recurrence definition: Wheal episodes ≥2 times per week. Recurrence rate = Number of recurrence cases / Number of cured cases × 100%. Adverse reaction rate = Number of adverse reactions / Total cases × 100%. |
| 49 | Yang Yang | 2020 | Epinastine Tablets 10 mg per dose, twice daily orally. 2 weeks per course of treatment. | 1. Acupoint catgut embedding. Primary acupoints: Bilateral Xuehai (SP10), Geshu (BL17), Sanyinjiao (SP6), Fengmen (BL12). Pattern-based supplementary points: Wind-heat pattern - add Quchi (LI11); Wind-cold pattern - add Feishu (BL13); Blood deficiency pattern - add Pishu (BL20), Zusanli (ST36); Thoroughfare and Conception Vessel disharmony - add Ganshu (BL18), Shenshu (BL23). 6-8 acupoints per treatment, once weekly, 2 treatments per course. 2. Yangxue Xiaxun Decoction (Blood-Nourishing Urticaria-Eliminating Decoction). Formula: Huangqi (Radix Astragali) 15 g, Danggui (Radix Angelicae Sinensis) 12 g, Heshouwu (Radix Polygoni Multiflori) 12 g, Shengdi (Radix Rehmanniae) 12 g, Jixueteng (Caulis Spatholobi) 12 g, Chuanxiong (Rhizoma Chuanxiong) 6 g, Baishao (Radix Paeoniae Alba) 12 g, Guizhi (Ramulus Cinnamomi) 12 g, Jiangcan (Bombyx Batryticatus) 10 g, Chantui (Periostracum Cicadae) 8 g, Fangfeng (Radix Saposhnikoviae) 12 g, Gancao (Radix Glycyrrhizae) 5 g. Modifications: Wind-cold - add Shufuzi (Radix Aconiti Lateralis Praeparata), Baizhu (Rhizoma Atractylodis Macrocephalae); Wind-heat - add Huangqin (Radix Scutellariae), Zhizi (Fructus Gardeniae), Mudanpi (Cortex Moutan); Blood deficiency with wind-dryness - add Digupi (Cortex Lycii), Maidong (Radix Ophiopogonis); Thoroughfare-Conception disharmony - add Chaihu (Radix Bupleuri), Yujin (Radix Curcumae). Administration: 1 dose daily, decocted in water, divided into morning and evening doses. Take 5 days, rest 2 days. 2 weeks per course. |  | Symptom Score Reduce Index (SSRI): SSRI = (Pre-treatment total score - Post-treatment total score) ÷ Pre-treatment total score × 100%. Cured: SSRI > 90%. Markedly effective: 60% ≤ SSRI ≤ 90%. Improved: 20% ≤ SSRI ≤ 59%. Ineffective: SSRI < 20%. Total effective rate = (Cured + Markedly effective) cases / Total cases. Associated laboratory markers: Serum IgE, complement C3. |
| 50 | Hailian Yi | 2015 | Mizolastine 10 mg per dose, once daily orally. Continuous treatment for 4 weeks. | 1. Tianjiu (Acupoint Application Therapy). Medicinal powder: Cang'erzi (Fructus Xanthii), Baixianpi (Cortex Dictamni), Jingjie (Herba Schizonepetae), Baishao (Radix Paeoniae Alba), Baijiezi (Semen Sinapis), Gancao (Radix Glycyrrhizae), Guizhi (Ramulus Cinnamomi), Fangfeng (Radix Saposhnikoviae), Difuzi (Fructus Kochiae) - equal quantities, ground into powder, mixed with ginger juice to form paste. Acupoints: Sanyinjiao (SP6), Zusanli (ST36), Quchi (LI11), Geshu (BL17), Waiguan (SJ5), Feishu (BL13). Application duration: 1-2 hours per treatment, remove when skin reddens or develops small blisters. Once every 10 days, total of 4 treatments. 2. Autohemotherapy: Draw 5 mL of patient's own venous blood, inject into Zusanli acupoint. Twice weekly, total of 4 treatments. |  | Markedly effective: Rash and pruritus basically disappeared. Improved: Rash and pruritus significantly alleviated. Ineffective: No improvement in rash and pruritus. Total effective rate = (Markedly effective + Improved) / Total cases × 100%. Recurrence rate followed up at 6 months after treatment completion. |
| 51 | Jianfeng Dai | 2020 | Levocetirizine Hydrochloride Tablets (Suzhou Dongrui Pharmaceutical Co., Ltd., National Medicine Approval No. H20060333, specification 5 mg). Dosage: 5 mg per dose, once daily. | One-time acupoint catgut embedding therapy. Primary acupoints: Zusanli (ST36), Sanyinjiao (SP6), Ashi points, Feishu (BL13). Supplementary points selected based on pattern differentiation: Blood-heat pattern - Hegu (LI4), Quchi (LI11); Wind-cold pattern - Guanyuan (CV4), Dazhui (GV14); Excess-heat pattern - Zhongwan (CV12), Shangjuxu (ST37). Procedure: After acupoint disinfection, place 2-3 cm catgut suture at the front of the needle tube. Lift the skin with left thumb, rapidly insert the needle into the skin. After needling sensation is achieved, push the stylet while withdrawing the needle tube, embedding the catgut into the corresponding acupoint. |  | Clinical efficacy criteria: ① Markedly effective: No adverse reactions such as swelling or drowsiness. ② Effective: Few and mild adverse reactions of swelling and drowsiness. ③ Ineffective: Frequent and severe adverse reactions of swelling and drowsiness. Total effective rate = (① + ②) / (① + ② + ③) × 100%. |
| 52 | Yanjie Shang | 2019 | Loratadine Tablets 10 mg per dose, once daily, taken orally at bedtime. Both groups received 2 courses of treatment, 2 weeks per course, with 1 day rest between courses. | Acupoints: Quchi (LI11), Xuehai (SP10), Zusanli (ST36), Fengmen (BL12), Feishu (BL13), Geshu (BL17), Pishu (BL20). Materials: No. 7 embedding needle, 3-0 catgut suture 2 cm. Procedure: Acupoint disinfection. Perpendicular insertion / oblique insertion for back points. After deqi, withdraw needle tube and push suture to embed in subcutaneous muscle layer. Stop bleeding with dry cotton ball and apply adhesive bandage. 2 weeks per course, total of 2 courses, 1 day rest between courses. Acupoints should not get wet for 24 hours after embedding. Strenuous exercise prohibited for 1-3 days. |  | Symptom Score Reduce Index (SSRI) = (Pre-treatment score - Post-treatment score) / Pre-treatment score × 100%. Cured: SSRI ≥ 90%. Markedly effective: 90% > SSRI ≥ 60%. Effective: 60% > SSRI ≥ 30%. Ineffective: SSRI < 30%. Total effective rate = (Clinical cure + Markedly effective + Effective) cases ÷ Total cases in group × 100%. |
| 53 | Qianhong Wu | 2016 | Western medicine (Desloratadine Tablets) treatment. Usage: Orally, once daily, 5 mg (1 tablet) per dose. Continuous treatment for 1 month. | Acupoint autohemotherapy. Procedure: Use 10 mL syringe to draw 4 mL of patient's own venous blood. After routine skin disinfection, inject the well-mixed venous blood into corresponding acupoints (contralateral Quchi (LI11), Zusanli (ST36), Xuehai (SP10)). Local soreness, distension, and numbness indicate optimal effect. 1 mL per acupoint. Once daily, continuous treatment for 1 month. |  | Markedly effective: Clinical symptoms completely disappeared after treatment, no recurrence within 6 months. Effective: Clinical symptoms improved, 1-2 recurrences within 6 months, self-resolving without treatment. Ineffective: No change in symptoms, or recurrent episodes. Effective rate calculated as percentage of markedly effective and effective cases among total cases. |
| 54 | Yujia Zhen | 2019 | Desloratadine Tablets, once daily, 1 tablet (5 mg) per dose. Continuous treatment for 4 weeks. | Observation group received acupoint autohemotherapy: 5 mL syringe draws 4 mL of own venous blood. Routine disinfection. Cross-alternating injection into bilateral Quchi (LI11) and Xuehai (SP10). 2 mL per acupoint. Once daily, 4 consecutive treatments per week, total of 4 weeks. |  | Efficacy index = [(Pre-treatment score - Post-treatment score) / Pre-treatment score] × 100%. Cured: Efficacy index ≥ 90%. Markedly effective: Efficacy index 60%-89%. Improved: Efficacy index 30%-59%. Ineffective: Efficacy index ≤ 29%. Total effective rate = Cure rate + Markedly effective rate. Symptom scoring: Pruritus, wheal number, and wheal diameter scored on 0-3 four-grade scale. |
| 55 | Feiying Hu | 2023 | Conventional acupuncture, same acupoints as observation group. Both observation and control groups received 2 courses of treatment, 2 weeks per course, 1 day rest between courses. | Yang needling at Quchi combined with heat-sensitive moxibustion. Acupoint selection references the 13th Five-Year national standard textbook "Acupuncture and Moxibustion" and international standardization scheme for scalp acupoints. Scalp points: Posterior oblique line of vertex-temporal (Baihui DU20 through Qubin GB7). Body points: Bilateral Quchi (LI11), Hegu (LI4), Xuehai (SP10), Weizhong (BL40), Geshu (BL17), Dazhui (GV14), Fengmen (BL12), Feishu (BL13). Quchi treated with yang needling method. Scalp points with through-needling. Remaining points with conventional acupuncture. Heat-sensitive moxibustion applied during needle retention. ① Quchi yang needling: Insert 1 needle perpendicularly at center of Quchi. Then shallowly insert 1 needle each at approximately 1 cm above, below, left, and right of Quchi, with needle tips toward center. ② Heat-sensitive moxibustion: Ignite moxa stick, apply moxibustion 2-3 cm from acupoint skin. Start with sparrow-pecking, circular, and meridian-traveling moxibustion for 30 seconds each to activate heat sensitization. Select 2 sensitive acupoints for moxibustion: first circular, then sparrow-pecking, then warm moxibustion, until heat sensitivity disappears. Both groups: 2 courses, 2 weeks per course, 1 day rest between courses. |  | UAS7 score used as symptom score to calculate Symptom Score Reduce Index (SSRI), i.e., efficacy index. Calculation references Nimodipine method. SSRI = (Pre-treatment score - Post-treatment score) / Pre-treatment score × 100%. Cured: SSRI ≥ 90%. Markedly effective: 90% > SSRI ≥ 60%. Effective: 60% > SSRI ≥ 30%. Ineffective: SSRI < 30%. |
| 56 | Qingshan Kong | 2011 | Cetirizine Tablets 10 mg per dose, once daily orally. 2 weeks of medication per course, continuous 2 courses. | (1) Internal administration of Yangxue Qufeng Decoction (Blood-Nourishing Wind-Dispelling Decoction): Huangqi (Radix Astragali) 20 g, Baizhu (Rhizoma Atractylodis Macrocephalae) 12 g, Fangfeng (Radix Saposhnikoviae) 10 g, Danggui (Radix Angelicae Sinensis) 15 g, Shudihuang (Radix Rehmanniae Praeparata) 10 g, Chuanxiong (Rhizoma Chuanxiong) 8 g, Baishao (Radix Paeoniae Alba), Jixueteng (Caulis Spatholobi), Baijili (Fructus Tribuli), Chantui (Periostracum Cicadae) - 15 g each. 1 dose daily, decocted in water, taken orally twice daily (morning and evening). (2) Acupoint pricking blood cupping: Quchi (LI11), Xuehai (SP10), Zusanli (ST36), Sanyinjiao (SP6). Routine skin disinfection. Three-edged needle pricks 3-5 times around acupoint and 5 mm surrounding area. Vacuum cup retained for 5 minutes. Blood volume: 0.5-2 mL. Once weekly. 2 weeks of medication and 2 pricking cupping treatments per course. Continuous 2 courses. |  | Four-grade scoring method: ① Pruritus: 0 points = no itching; 1 point = mild; 2 points = moderate; 3 points = severe. ② Wheal size: <0.5 cm = 1 point; 0.5-2 cm = 2 points; >2 cm = 3 points. ③ Wheal number: 0 = 0 points; 1-6 = 1 point; 7-12 = 2 points; >12 = 3 points. ④ Wheal duration: No wheals = 0 points; ≤1 h = 1 point; ≤12 h = 2 points; >12 h = 3 points. Sum of four items = total score. SSRI = [(Pre-treatment total score - Post-treatment total score) / Pre-treatment total score] × 100%. Cured: SSRI > 90%. Markedly effective: SSRI 60%-90%. Improved: SSRI 20%-59%. Ineffective: SSRI < 20%. Markedly effective rate = Cure rate + Markedly effective rate. |
| 57 | Xu Kang | 2018 | Mizolastine 10 mg per dose, once daily orally. Continuous medication for 4 weeks. | 1. Tianjiu (Acupoint Application Therapy). Medicinal powder formula: Cang'erzi (Fructus Xanthii), Baixianpi (Cortex Dictamni), Jingjie (Herba Schizonepetae), Baishao (Radix Paeoniae Alba), Baijiezi (Semen Sinapis), Gancao (Radix Glycyrrhizae), Guizhi (Ramulus Cinnamomi), Fangfeng (Radix Saposhnikoviae), Difuzi (Fructus Kochiae). Ground into powder, mixed with ginger juice to form paste. Acupoints: Sanyinjiao (SP6), Zusanli (ST36), Quchi (LI11), Geshu (BL17), Waiguan (SJ5), Feishu (BL13). Procedure: Apply for 1-2 hours, remove when skin reddens or develops small blisters. Once every 10 days, total of 4 treatments. 2. Autohemotherapy: Draw 5 mL of own venous blood, inject into Zusanli acupoint. Twice weekly, total of 4 treatments. |  | Markedly effective: Symptoms such as rash and skin pruritus basically disappeared. Improved: Symptoms such as rash and skin pruritus significantly alleviated. Ineffective: No improvement in symptoms such as rash and skin pruritus. Total effective rate = (Markedly effective + Improved) cases ÷ Total cases × 100%. Additional observation: Recurrence rate followed up at 6 months after treatment completion. |
| 58 | Minyan Wu | 2019 | Desloratadine Citrate Tablets 8.8 mg, once daily. Continuous treatment for 1 month. | 1. Modified Yupingfeng San combined with Guizhi Decoction. Base formula: Huangqi (Radix Astragali) 40 g, Dangshen (Radix Codonopsis) 15 g, Baizhu (Rhizoma Atractylodis Macrocephalae) 15 g, Fangfeng (Radix Saposhnikoviae) 20 g, Jingjie (Herba Schizonepetae) 20 g, Guizhi (Ramulus Cinnamomi) 15 g, Baishao (Radix Paeoniae Alba) 15 g, Danggui (Radix Angelicae Sinensis) 15 g, Wumei (Fructus Mume) 15 g, Wuweizi (Fructus Schisandrae) 15 g, Baijili (Fructus Tribuli) 15 g, Baixianpi (Cortex Dictamni) 15 g, Fuping (Herba Spirodelae) 10 g, Dazao (Fructus Jujubae) 6 pieces, Shengjiang (Rhizoma Zingiberis Recens) 5 slices, Zhigancao (Radix Glycyrrhizae Praeparata) 6 g. Modifications: Severe pruritus - add Wugong (Scolopendra) 3 g; Cold-induced - add Mahuang (Herba Ephedrae) 10 g; Nighttime pruritus - add Shechuangzi (Fructus Cnidii); Dampness predominant - add Fuling (Poria), Sangbaipi (Cortex Mori); Qi deficiency - increase Dangshen and Baizhu. Administration: 1 dose daily, decoct to obtain 300 mL, divided into morning and evening doses. Course: 1 month. 2. Abdominal acupuncture. Primary points: Zhongwan (CV12), Xiawan (CV10), Qihai (CV6), Guanyuan (CV4). Supplementary points: Huaroumen (ST24), Wailing (ST26), Daheng (SP15) - bilateral. No. 34 abdominal needles. Supine position, insert to earth level. Wait for qi, move qi, promote qi. Retain needles for 30 minutes. Once daily. 5 days treatment, 2 days rest per course. Continuous 4 courses. |  | Cured: All wheals subsided, no pruritus or erythema. Effective: Wheals subsided ≥ 30%, pruritus and erythema alleviated. Ineffective: Wheals subsided < 30%, no improvement or worsening of symptoms. Total effective rate = (Cured + Effective) cases / Total cases × 100%. Associated indicators: Symptom score (pruritus, wheal size/number/duration), DLQI quality of life, serum IgE, IL-4, IFN-γ. Recurrence rate followed up at 3 months after treatment. |
| 59 | Haiyan Gu | 2020 | Loratadine Tablets 10 mg, once daily. Continuous treatment for 6 weeks. | Acupoints: Fengchi (GB20), Fengmen (BL12), Fengfu (GV16), Bingfeng (SI12), Yifeng (SJ17), Fengshi (GB31). Needles: 0.25 mm × 40 mm Hua Tuo filiform needles. Yang-hiding-yin compound manipulation. Once daily, 5 days per week, continuous 6 weeks. |  | Efficacy index = (Pre-treatment total score - Post-treatment total score) ÷ Pre-treatment total score × 100%. Cured: Efficacy index ≥ 90%. Markedly effective: 60% ≤ Efficacy index < 90%. Effective: 30% ≤ Efficacy index < 60%. Ineffective: Efficacy index < 30%. Total effective rate = (Cured + Markedly effective + Effective) / Total cases × 100%. Scoring items: Wheal number, size, episode frequency, duration, pruritus. Associated tests: IgE, IL-4, IFN-γ. Recurrence followed up at 1 month after treatment. |
| 60 | Yu Li | 2023 | Levocetirizine Hydrochloride Tablets 5 mg, once daily. Continuous treatment for 4 weeks. | 1. Acupuncture. Acupoints: Bilateral Quchi (LI11), Xuehai (SP10), Zusanli (ST36), Sanyinjiao (SP6), Hegu (LI4). 0.30 mm × 40 mm filiform needles, perpendicular insertion 1 cun. Even reinforcing-reducing method. Retain needles for 30 minutes, twist-rotate every 10 minutes. 3 times per week. 2. Pricking bloodletting: Prone position, three-edged needle pricking at Geshu (BL17) and Weizhong (BL40). 3-5 drops bleeding per acupoint. Iodophor disinfection. Twice weekly. 3. Internal herbal medicine: Hospital preparation Xuchangqing Mixture, 30 mL, twice daily orally. Unified course: 4 weeks. |  | Efficacy index calculated by UAS7 total score: Efficacy index = (Pre-treatment UAS7 total score - Post-treatment UAS7 total score) ÷ Pre-treatment UAS7 total score × 100%. Cured: Efficacy index ≥ 90%. Markedly effective: 60% ≤ Efficacy index < 90%. Effective: 20% ≤ Efficacy index < 60%. Ineffective: Efficacy index < 20%. Associated observation indicators: UAS7, DLQI, UCT scores, serum IgE, blood EOS absolute value/percentage. |
| 61 | Liangnan Zhang | 2019 | Loratadine Tablets 10 mg, once daily. Continuous treatment for 6 weeks. | Acupoints: Fengchi (GB20), Fengmen (BL12), Fengfu (GV16), Bingfeng (SI12), Yifeng (SJ17), Fengshi (GB31). Needles: 0.25 mm × 40 mm filiform needles. Yang-hiding-yin manipulation. Once daily, 5 times per week, continuous 6 weeks. |  | Score reduction rate calculated by total symptom score. Cured: Score reduction ≥ 90%. Markedly effective: 60% ≤ Score reduction < 90%. Effective: 30% ≤ Score reduction < 60%. Ineffective: Score reduction < 30%. Scoring includes wheal number, size, episode frequency, duration. Associated tests: Serum IL-4, IgE, IFN-γ. |
| 62 | Wenyin Wu | 2020 | Loratadine Tablets 10 mg, once daily. Continuous treatment for 8 weeks. | Acupoints: Fengchi (GB20), Fengmen (BL12), Fengfu (GV16), Bingfeng (SI12), Yifeng (SJ17), Fengshi (GB31). Needles: 0.35 mm × 40 mm Hua Tuo filiform needles. Insertion depth: Fengchi - toward nasal tip, 10 mm; Fengmen - oblique insertion, 10 mm; Fengfu - perpendicular insertion, 10 mm; Bingfeng - perpendicular insertion, 10 mm; Yifeng - toward nasal tip, 10 mm; Fengshi - perpendicular insertion, 20 mm. Manipulation: Lifting-thrusting, twisting-rotating reducing method. Retain needles for 20 minutes. Frequency: 5 times per week, continuous 8 weeks. |  | Efficacy index = (Pre-treatment symptoms + DLQI total score - Post-treatment total score) ÷ Pre-treatment total score × 100%. Cured: Efficacy index ≥ 90%. Markedly effective: 60% ≤ Efficacy index ≤ 89%. Effective: 30% ≤ Efficacy index ≤ 59%. Ineffective: Efficacy index < 30%. Scoring: Wheal size, number, pruritus, duration - 0/2/4/6 points each. Associated: DLQI quality of life scale, serum IL-4, IgE, IFN-γ. |
| 63 | Libin Gu | 2019 | Loratadine Tablets 10 mg, once daily. Continuous treatment for 6 weeks. | Acupoints: Jianyu (LI15), Quchi (LI11), Shousanli (LI10). Needles: 0.30 mm × 40 mm disposable filiform needles. Manipulation: Yang-hiding-yin method. First insert 1 cun, slow press and tight lift for six yin counts. Withdraw to 0.5 cun, tight press and slow lift for nine yang counts until deqi. Retain needles for 20 minutes, manipulate once every 10 minutes. Frequency: 5 times per week, total 6 weeks of treatment. |  | Efficacy index = (Pre-treatment total symptom score - Post-treatment total symptom score) ÷ Pre-treatment total symptom score × 100%. Cured: Efficacy index ≥ 90%. Markedly effective: 60% ≤ Efficacy index ≤ 89%. Effective: 30% ≤ Efficacy index ≤ 59%. Ineffective: Efficacy index < 30%. Total effective rate = (Cured + Markedly effective + Effective) / Total cases × 100%. Scoring items: Wheal number, wheal size, pruritus severity - 0-3 points each, sum of three items = total score. Associated tests: Serum IL-4, IgE, IFN-γ. |
| 64 | Zixing Liu | 2021 | Cetirizine Tablets 10 mg, once daily orally. 10 days per course, 2-3 days rest between courses, continuous 3 courses. | 1. Electroacupuncture at Back-Shu points. Acupoints: Feishu (BL13), Pishu (BL20), Xinshu (BL15), Shenshu (BL23), Ganshu (BL18). No. 30 1.5 cun filiform needles, perpendicular insertion 0.8-1.2 cun. Lifting-thrusting reinforcing method until deqi. Connect to dense-disperse wave electroacupuncture for 20 minutes. Once daily, 10 days per course, 2-3 days interval, total 3 courses. 2. Pricking blood cupping: First moving cupping along back Bladder Meridian and Governor Vessel until skin flushing. Plum blossom needle light tapping along meridians until slight oozing, then apply medium/large fire cups for 5-10 minutes. Supplementary point modifications: Facial swelling and fever - add ear apex three-edged needle bloodletting 20-30 drops; Upper limb predominant - add Quchi pricking and cupping; Lower limb predominant - add Weizhong bloodletting 1-3 mL. Frequency: Once every 3 days, 4 treatments per course. Menstrual period and 24 hours after bloodletting: no water contact. |  | Cured: Wheals completely subsided, no recurrence at 6-month follow-up. Effective: Wheals reduced by more than 30%, pruritus relieved, episode intervals prolonged. Ineffective: No improvement or worsening of wheals and pruritus. Total effective rate = (Cured + Effective) / Total cases × 100%. Associated tests: Serum IL-4, IFN-γ, IgE. |
| 65 | Yuming Wang | 2018 | Loratadine Tablets, once daily orally. Continuous treatment for 6 weeks. | Acupoints (bilateral): Shaofu (HT8), Shenmen (HT7), Tongli (HT5), Daling (PC7), Quchi (LI11), Hegu (LI4), Xuehai (SP10), Zusanli (ST36), Sanyinjiao (SP6). Needles: 0.25 mm × 40 mm filiform needles, perpendicular insertion 1 cun. Even reinforcing-reducing method. Manipulate once every 10 minutes. Retain needles for 30 minutes. Frequency: Every other day, 3 times per week, 18 treatments over 6 weeks. |  | UAS Urticaria Activity Score (pruritus and wheal number, 0-3 points each, total 0-6 points). SSRI score reduction index for determination: Ineffective: SSRI < 20%; Improved: 20% ≤ SSRI < 60%; Markedly effective: 60% ≤ SSRI < 90%; Cured: SSRI ≥ 90%. Total effective rate = Cured + Markedly effective cases percentage. Recurrence rate followed up at 12 weeks after treatment. |
| 66 | Huanmin Huo | 2014 | Loratadine 10 mg, once daily orally. Continuous treatment for 4 weeks. | 1. Acupuncture: Quchi (LI11), Neiguan (PC6), Xuehai (SP10), Sanyinjiao (SP6). Reducing method. Retain needles for 30 minutes. 2. Bloodletting cupping: Prone position, three-edged needle pricking at Feishu (BL13) and Geshu (BL17). Cupping to aspirate 2-3 mL of stagnant blood. 3. Frequency: Every other day. Course: 4 weeks. |  | SSRI = [(Pre-treatment total score - Post-treatment total score) ÷ Pre-treatment total score] × 100%. Individual 4-point scale (wheal number, size, pruritus, episode frequency, duration, 0-3 points each). Cured: SSRI ≥ 90%; Markedly effective: 60% ≤ SSRI < 90%; Effective: 20% ≤ SSRI < 60%; Ineffective: SSRI < 20%. Cure-marked effective rate = (Cured + Markedly effective) / Total cases × 100%. |
| 67 | Yun Yao | 2021 | Desloratadine Citrate Tablets 5 mg, taken at bedtime every night. 20 days per course, total of 2 courses. | 1. Acupuncture. Acupoints: Quchi (LI11), Xuehai (SP10), Zusanli (ST36), Fengshi (GB31), Dazhui (GV14). Needles: 0.30 mm × 40 mm. Quchi/Xuehai: perpendicular insertion 15-20 mm; Zusanli/Fengshi: perpendicular insertion 20-30 mm; Dazhui: oblique insertion 10-15 mm. Dazhui: twisting-rotating reinforcing method. Remaining points: even reinforcing-reducing method. Retain needles for 30 minutes. 2. Heat-sensitive moxibustion. Same acupoints. Start with sparrow-pecking 30 s + circular 30 s + meridian-traveling moxibustion 30 s. After penetrating heat and soreness-distension sensation appear, apply warm moxibustion until sensation disappears. 3. Frequency and course: Every other day, 10 treatments per course, total 2 courses. |  | Efficacy index calculated by Nimodipine method: Efficacy index = (Pre-treatment score - Post-treatment score) / Pre-treatment score × 100%. Cured: No wheals, erythema, or pruritus on cold exposure, basically disappeared, efficacy index ≥ 95%. Markedly effective: Wheals significantly reduced on cold exposure, 70% ≤ efficacy index < 95%. Effective: Wheals and pruritus alleviated on cold exposure, 30% ≤ efficacy index < 70%. Ineffective: No improvement in symptoms, efficacy index < 30%. Total effective rate = (Cured + Markedly effective + Effective) cases / Total cases × 100%. Associated observations: Symptom score, serum IgE, adverse reactions, recurrence rate at 3 months. |
| 68 | Sikkintana | 2016 | Loratadine Tablets 10 mg per dose, once daily. Dietary restrictions: No alcohol, no fish, shrimp, crab or other triggering foods. Course: 7 days per course, 2 days interval between courses, total 2 courses (14 days). | 1. Acupuncture protocol. Acupoints: Bilateral Xuehai (SP10), Quchi (LI11), Hegu (LI4), Zusanli (ST36), Sanyinjiao (SP6), Baihui (GV20). Needles: 0.35 mm × 40 mm Hua Tuo filiform needles. Manipulation: Quchi, Hegu - reducing method; Zusanli, Baihui - reinforcing method; Xuehai, Sanyinjiao - even reinforcing-reducing method. Retain needles for 30 minutes after deqi. Frequency: Once daily, 7 days per course, 2 days interval, total 2 courses. 2. Xiaofeng San formula. Jingjie (Herba Schizonepetae), Fangfeng (Radix Saposhnikoviae), Chantui (Periostracum Cicadae), Zhimu (Rhizoma Anemarrhenae), Niubangzi (Fructus Arctii) - 15 g each; Danggui (Radix Angelicae Sinensis) 20 g; Shudi (Radix Rehmanniae Praeparata), Gancao (Radix Glycyrrhizae) - 10 g each; Difuzi (Fructus Kochiae) 15 g; Baixianpi (Cortex Dictamni) 9 g; Danshen (Radix Salviae Miltiorrhizae) 10 g; Chishao (Radix Paeoniae Rubra) 15 g; Baijili (Fructus Tribuli), Jiangcan (Bombyx Batryticatus) - 9 g each; Dangshen (Radix Codonopsis) 15 g; Huangqi (Radix Astragali), Baizhu (Rhizoma Atractylodis Macrocephalae) - 20 g each. Administration: 1 dose daily, decoct to obtain 300 mL, divided into morning and evening doses. Course same as acupuncture. |  | Referenced from "Diagnostic and Efficacy Standards for TCM Diseases and Syndromes". Cured: Rash completely subsided, no pruritus. Markedly effective: Rash basically subsided, occasional itching. Effective: Rash subsiding slowly, skin scratch marks visible. Ineffective: No improvement in symptoms, rash comes and goes, severe pruritus. Total effective rate = (Cured + Markedly effective + Effective) ÷ Total cases × 100%. Efficacy assessed after 2 consecutive courses (14 days) of treatment. |
| 69 | Wuming Xia | 2017 | Cyproheptadine Cream for external use, apply to affected area twice daily. Continuous treatment for 7-10 days. | Combined acupuncture + Shenque cupping therapy, 7-10 days course. 1. Acupuncture: Primary points - Xuehai (SP10), Hegu (LI4), Weizhong (BL40). Filiform needle acupuncture, retain needles for 20-30 minutes, manipulate 3 times. Pattern-based supplementary points: Difficulty breathing - add Tiantu (CV22); Blood deficiency generating wind - add Zusanli (ST36), Sanyinjiao (SP6). 2. Shenque cupping: Cupping at Shenque (CV8) umbilicus. 3 cupping sessions total, 4-5 minutes per single retention. |  | Cured: Clinical symptoms basically disappeared, no recurrence. Markedly effective: Clinical symptoms subsided by more than 80%, no recurrence. Effective: Clinical symptoms subsided by 50%-80%, no recurrence. Ineffective: Clinical symptoms subsided by less than 50%, with recurrence. Total effective rate = (Cured + Markedly effective + Effective) ÷ Total cases × 100%. |
| 70 | Xin Zhang | 2020 | Cetirizine Hydrochloride Tablets 10 mg, once daily orally. Continuous 6 weeks. | Acupuncture acupoints: Quchi (LI11), Waiguan (SJ5), Xuehai (SP10), Zusanli (ST36), Sanyinjiao (SP6), Zhongwan (CV12), Tianshu (ST25), Guanyuan (CV4). 0.25 mm × 40 mm filiform needles, conventional acupuncture, retain 20 minutes. After acupuncture, Shenque cupping for 5 minutes. Plum blossom needle tapping at bilateral Geshu (BL17) until slight oozing, then cupping for 5 minutes. Treatment frequency: Every other day, 3 times per week, 6 weeks course. | Acupuncture, cupping, pricking bloodletting procedures, acupoints, frequency, and course identical to acupuncture group. Simultaneous oral Cetirizine Hydrochloride Tablets 10 mg, once daily, continuous 6 weeks. | Based on "Guiding Principles for Clinical Research of New Chinese Medicines", efficacy determined by Symptom Score Reduce Index. Formula: SSRI = (Pre-treatment score - Post-treatment score) ÷ Pre-treatment score × 100%. Cured: SSRI ≥ 90%. Markedly effective: 60% ≤ SSRI < 90%. Effective: 20% ≤ SSRI < 60%. Ineffective: SSRI < 20%. Total effective rate = (Cured + Markedly effective + Effective) cases / Total cases. Cure-marked effective rate = (Cured + Markedly effective) cases / Total cases. |
| 71 | Jia Xu | 2005 | Chlorpheniramine 4 mg, Calcium Gluconate 2 g, 3 times daily, orally. Total 4 weeks. | (Divided into acupuncture and autohemotherapy acupoint injection). 1. Acupuncture. Acupoints: Wind pathogen external invasion pattern - Quchi (LI11), Hegu (LI4), Xuehai (SP10), Sanyinjiao (SP6), Geshu (BL17). Gastrointestinal accumulated heat pattern - Zhongwan (CV12), Shangjuxu (ST37), Hegu (LI4), Zusanli (ST36). Yin deficiency fire effulgence pattern - Xuehai (SP10), Geshu (BL17), Zusanli (ST36), Sanyinjiao (SP6), Taichong (LR3), Taixi (KI3). Method: Xuehai, Geshu, Taixi - reinforcing method; other points - reducing method. After deqi, connect to G6805 electroacupuncture device for 30 minutes. 5 times per week, total 4 weeks. 2. Autohemotherapy acupoint injection. Acupoints: Quchi (LI11), Zusanli (ST36), Xuehai (SP10), Feishu (BL13). Patient supine, draw blood from one side median cubital vein. After routine skin disinfection, use syringe to draw 2 mL autologous blood. Rapidly perpendicularly insert 1-1.5 cun at acupoint, slight lifting-thrusting. After deqi sensation, confirm no blood return by aspiration, then slowly inject autologous blood. 5 mL per acupoint per treatment. Above acupoints used alternately. Every other day, 3 times per week, total 4 weeks. |  | Formulated according to "Diagnostic and Efficacy Standards for TCM Diseases" issued by the State Administration of Traditional Chinese Medicine. Cured: Wheals subsided, clinical signs disappeared, no recurrence within 6 months. Improved: Wheals subsided by 30% or recurrence interval prolonged after subsiding, pruritus and other symptoms alleviated. Ineffective: No significant change in wheals and pruritus, or wheals subsided by less than 30%. |
| 72 | Zhiling Sun | 2017 | Loratadine Tablets (Claritin, produced by Schering-Plough Pharmaceutical Co., Ltd., Shanghai) 10 mg, once daily, orally. | Shengui Lizhong Pills (produced by Tongrentang Pharmaceutical Factory, Beijing Tongrentang Pharmaceutical Co., Ltd.) 6 g, twice daily, orally. Simultaneous acupuncture at Sima points. Sima points divided into upper, middle, and lower 3 points, 6 points total on left and right. One side points per treatment, opposite side next day. Perpendicular insertion 0.8-2.5 cun. Practitioner feels heavy and tight sensation. Retain needles for 30 minutes. Once daily, 6 times per week. |  | Referenced from "TCM Disease Diagnostic Standards and Formula Selection". Cured: Wheals subsided, clinical signs disappeared, no recurrence for 3 months. Improved: Wheals subsided by 30% or recurrence interval prolonged after subsiding, pruritus and other symptoms alleviated. Unimproved: No significant improvement in wheals and pruritus, or subsided by less than 30%. |
| 73 | Ying Liang | 2018 | Loratadine Tablets (Yangtze River Pharmaceutical, National Medicine Approval No. H20080134, specification: 10 mg/tablet) 10 mg, taken 30 minutes after meals, once daily. Continuous 14 days. | Stuck needle combined with Back-Shu point bloodletting therapy. (1) Acupoints: Quchi (LI11), Xuehai (SP10), Zusanli (ST36), Sanyinjiao (SP6), Hegu (LI4), Geshu (BL17), Feishu (BL13), Pishu (BL20). (2) Stuck needle method: Patient supine. Select Quchi, Xuehai, Zusanli, Sanyinjiao, Hegu. Disinfect local skin with 75% alcohol cotton ball. Use 2-cun (0.25 mm × 50 mm, Hua Tuo brand) acupuncture needles for conventional needling. Rapid perpendicular insertion, 1.0-1.5 cun depth. After lifting-thrusting and twisting-rotating to produce soreness, numbness, distension, and pain, twist needle handle in single direction for 2 or more rotations (number of rotations determined by patient's needle sensation intensity and practitioner's feeling of needle tightness). Create "needle sinking tightness, cannot pull out, cannot advance further" stuck needle state. Patient feels instantaneous enhancement of soreness, numbness, distension, and pain at needling site. Then retain needles for 30 minutes. At needle withdrawal, gently rotate needle handle to loosen needle body, rapidly withdraw. Disinfect treatment area with 75% alcohol. If bleeding, press with sterile cotton ball to stop bleeding. (3) Back-Shu point bloodletting: After stuck needle therapy, patient prone. Disinfect Geshu, Feishu, Pishu skin with 75% alcohol. Prick each acupoint with disposable injection needle, then apply fire cups. Approximately 10 minutes, 3-4 mL bloodletting as optimal. After removing cups, wipe local area with sterile cotton ball to avoid infection. Stuck needle therapy: once daily. Bloodletting therapy: every other day. Total 14 days of treatment. |  | Efficacy criteria according to urticaria (yinzhen) efficacy standards in "Diagnostic and Efficacy Standards for TCM Diseases". Cured: Wheals subsided, clinical symptoms and signs disappeared, no further episodes. Improved: Wheals subsided by 30% or recurrence interval prolonged after subsiding, pruritus and other symptoms alleviated. Unimproved: No significant improvement in wheals and pruritus, or subsided by less than 30%. Total effective rate = Cure rate + Improvement rate. |
| 74 | Jie Wang | 2011 | Cetirizine Tablets 10 mg per dose, once daily. Both groups: 3 weeks per course. Avoid spicy and stimulating foods during treatment. Efficacy statistics after course completion. | (1) Self-composed Qufeng Zhiyang Decoction (Wind-Dispelling Itch-Stopping Decoction). Prescription: Chao Jingjie (fried Herba Schizonepetae) 10 g, Chao Fangfeng (fried Radix Saposhnikoviae) 10 g, Chantui (Periostracum Cicadae) 10 g, Fengfang (Nidus Vespae) 10 g, Cijili (Fructus Tribuli) 20 g, Difuzi (Fructus Kochiae) 10 g, Baixianpi (Cortex Dictamni) 10 g, Danggui (Radix Angelicae Sinensis) 10 g, Baizhi (Radix Angelicae Dahuricae) 10 g, Kushen (Radix Sophorae Flavescentis) 10 g, Huaihua (Flos Sophorae) 10 g, Gancao (Radix Glycyrrhizae) 6 g. Modifications: Damp-heat pattern - add Cangzhu (Rhizoma Atractylodis) 6 g, Huangbai (Cortex Phellodendri) 10 g; Wind-cold pattern - remove Kushen, Huaihua, add Zhi Mahuang (honey-fried Herba Ephedrae) 6 g, Guizhi (Ramulus Cinnamomi) 10 g; Blood deficiency with wind exuberance - remove Chao Jingjie, add Ejiao (Colla Corii Asini) 10 g, Zhi Heshouwu (prepared Radix Polygoni Multiflori) 15 g; Qi deficiency pattern - add Dangshen (Radix Codonopsis) 15 g, Huangqi (Radix Astragali) 30 g; Constipation - add Dahuang (Radix et Rhizoma Rhei) 5 g; Pruritus mild during day and severe at night - add Shengdi (Radix Rehmanniae) 10 g, Danpi (Cortex Moutan) 10 g; Heart vexation and insomnia - add Chao Zaoren (fried Semen Ziziphi Spinosae) 15 g, Yejiaoteng (Caulis Polygoni Multiflori) 20 g. 1 dose daily, decoct to obtain 400 mL, divided into morning and evening doses. (2) Acupoint catgut embedding therapy: After local acupoint epidermal anesthesia, use lumbar puncture needle to implant catgut suture into relevant acupoints. Following Huang Yanxia et al.'s acupoint selection experience: Bilateral Quchi (LI11), bilateral Xuehai (SP10), Feishu (BL13). After routine skin disinfection, grasp a 1-2 cm long sterilized catgut suture segment, place at front of lumbar puncture needle tube, then attach stylet. Left thumb and index finger tighten or pinch skin at insertion site. Right hand holds needle, inserts to required depth. After needle sensation appears, push stylet while withdrawing needle tube, implanting catgut in subcutaneous tissue or muscle layer of acupoint. Cover needle hole with adhesive bandage. |  | Efficacy assessed according to "Diagnostic and Efficacy Standards for TCM Diseases". Cured: Wheals subsided, clinical signs disappeared, no further episodes. Improved: Wheals subsided by 30% or recurrence interval prolonged after subsiding, pruritus and other symptoms alleviated. Unimproved: No significant improvement in wheals and pruritus, or subsided by less than 30%. |
| 75 | Yuanzheng Sun | 2014 | Control group received oral Chlorpheniramine Maleate Tablets (Sichuan Shenghe Pharmaceutical Co., Ltd., batch No. National Medicine Approval H51021060), 3 times daily, 4.0 mg per dose. External use: Prednisone Acetate Cream (Beijing Shuangji Pharmaceutical Co., Ltd., batch No. National Medicine Approval H11022175), apply appropriate amount to affected area, twice daily. 10 days per course, total 2 courses. | Autologous venous blood acupoint injection therapy. Selected acupoints: Zusanli (ST36) - bilateral, Xuehai (SP10) - bilateral. Method: Take one disposable 5 mL syringe. Following standard venipuncture procedure, draw 2 mL venous blood from median cubital vein. First locate acupoints for injection: Xuehai (SP10, Foot Taiyin Spleen Meridian - flex knee, 2 cun above medial superior border of patella, at prominence of medial head of quadriceps femoris). Zusanli (ST36, Foot Yangming Stomach Meridian - 3 cun below Dubi point, 1 finger-breadth lateral to anterior crest of tibia). Then strictly disinfect injection acupoint sites. Rapidly perpendicularly insert needle into acupoint. After insertion, use lifting-thrusting method for needling. When patient achieves deqi (local sensations of soreness, numbness, distension, heaviness), aspirate syringe to confirm no blood return. Slowly inject 0.5 mL venous blood. Withdraw needle, press injection site with sterile cotton ball to prevent bleeding or subcutaneous ecchymosis. Note: Needle sites should not get wet to avoid infection. Every other day, 10 treatments per course. Efficacy determined after 2 courses. |  | Cured: Skin pruritus and wheal lesions completely disappeared after treatment, dermatographism test (-), no recurrence at follow-up. Markedly effective: Skin pruritus disappeared after treatment. Mild episodes occur with dietary indiscretion (irritating foods, alcohol) or contact with allergens, self-resolving without treatment, dermatographism test (+). Effective: Skin pruritus disappeared after treatment. Episodes occur with dietary indiscretion or contact with allergens, require treatment to resolve, dermatographism test (+). Ineffective: No symptom improvement before and after treatment. |
| 76 | Guilian Liu | 2020 | Both groups of patients received conventional antihistamine treatment during outpatient period: diphenhydramine, cyproheptadine, chlorpheniramine, acrivastine, etc. A few refractory patients may be combined with cimetidine, ranitidine H₂ receptor antagonists. Control group received autohemotherapy: Patient supine position. Select Zusanli and Xuehai acupoints. Routine disinfection of median cubital vein area. Draw 2 mL venous blood, then rapidly insert needle into above acupoints. After needle sensation and confirming no blood return by aspiration, perform autohemotherapy injection. Only one acupoint injected per autohemotherapy session, once weekly. Zusanli and Xuehai used alternately. | Combined cupping at Back-Shu points on above basis. Details: During treatment, use No. 28-30 1-1.5 cun filiform needles for perpendicular insertion at Back-Shu points including Pishu (BL20), Xinshu (BL15), Feishu (BL13), Geshu (BL17), Shenshu (BL23). Insertion depth 0.8-1.2 cun. After deqi, perform small amplitude lifting-thrusting at deqi site. Then perform pricking blood cupping at patient's Back-Shu points, 3-5 mL bleeding as optimal. Once daily, 10 consecutive treatments per course, 2-3 days rest between adjacent courses. Best efficacy after 3 courses of continuous treatment. |  | 1. Treatment efficacy criteria: (1) Markedly effective: Efficacy index > 70%. (2) Effective: Efficacy index 30%-69%. (3) Ineffective: Efficacy index 0-29%. Efficacy index = (Pre-treatment score - Post-treatment score) / Pre-treatment score × 100%. 2. Compare treatment efficacy and changes in immune indicators such as IFN-γ, IL-4, and IgE between two groups. Specific scoring criteria: Strong burning sensation with pruritus, wheal diameter > 2 cm and number > 12 = 3 points; Moderate burning sensation with pruritus, wheal diameter 0.5-2.0 cm and number 7-12 = 2 points; Mild burning sensation and pruritus, wheal diameter < 0.5 cm and number < 2 = 1 point; No pruritus or wheal symptoms = 0 points. |
| 77 | Jin Huang | 2016 | Desloratadine Tablets 5 mg, once daily. Treatment for 8 weeks. | Oral self-composed Yinhuai Decoction combined with autohemotherapy. ① Oral Chinese medicine. Yinhuai Decoction formula: Jinyinhua (Flos Lonicerae) 15 g, Huaihua (Flos Sophorae) 10 g, Lianqiao (Fructus Forsythiae) 10 g, Bohe (Herba Menthae) 5 g (added later), Mudanpi (Cortex Moutan) 10 g, Chishao (Radix Paeoniae Rubra) 10 g, Lugen (Rhizoma Phragmitis) 10 g, Jingjie (Herba Schizonepetae) 10 g, Fangfeng (Radix Saposhnikoviae) 10 g, Chantui (Periostracum Cicadae) 5 g, Fuping (Herba Spirodelae) 10 g, Guizhi (Ramulus Cinnamomi) 10 g, Baishao (Radix Paeoniae Alba) 10 g. Light decoction. 1 dose daily, divided into 2 warm doses. ② Autohemotherapy. Procedure: Patient seated. After routine disinfection at patient's elbow median vein, use 5 mL syringe to draw 4 mL venous blood. Inject respectively into bilateral Zusanli (ST36) and Xuehai (SP10), 1 mL per acupoint. Local distension sensation as optimal. Once weekly. Total 8 weeks of treatment. |  | Symptom Score Reduce Index (SSRI) calculated based on symptom and sign scores before and after treatment. SSRI = (Pre-treatment symptom and sign score - Post-treatment symptom and sign score) / Pre-treatment symptom and sign score × 100%. Cured: Itching disappeared, SSRI ≥ 90%. Markedly effective: Itching significantly alleviated, SSRI ≥ 60% and < 90%. Improved: Itching alleviated, SSRI ≥ 20% and < 60%. Ineffective: Itching same as before or worsened, SSRI reduction < 20% or continues severe (no change or worsening). |
| 78 | Yunlong Cheng | 2012 | Loratadine 10 mg per dose, once daily. Continuous treatment for 30 days or discontinued after complete clinical symptom relief. | Under strict sterile conditions, use 10 mL syringe to draw 4 mL of patient's own venous blood. After shaking well, rapidly inject into corresponding acupoints, 1 mL per acupoint. Acupoint selection criteria: Formulated based on references [3] and [4]. Wind-heat type - bilateral Zusanli (ST36), Waiguan (SJ5). Wind-cold type - bilateral Zusanli (ST36), Quchi (LI11). Blood deficiency affected by wind type - bilateral Xuehai (SP10), Quchi (LI11). Once every 3 days, 10 treatments per course, total 1 course. Other medications discontinued during treatment. |  | Four-grade SSRI efficacy evaluation criteria. Basic cure: SSRI ≥ 90%. Markedly effective: 60% ≤ SSRI < 90%. Improved: 30% ≤ SSRI < 60%. Ineffective: SSRI < 30% (no change or worsening). Total effective rate calculated by basic cure and markedly effective cases. |
| 79 | Bifang Chen | 2022 | Ebastine Tablets (Kestine, Industrias Farmacéuticas Almirall, Spain, approval No. Registration Certificate H20140855, specification: 10 mg/tablet) orally, 1 tablet/day. Total 6 weeks. | Autohemotherapy acupoint injection combined with Xiaofeng San acupoint application. Autohemotherapy acupoint injection selects Dingchuan (EX-B1), Quchi (LI11), Zusanli (ST36). Patient seated. After routine elbow skin disinfection, use disposable 5 mL syringe to draw 3 mL cubital venous blood. Immediately inject into one side of pre-disinfected Dingchuan, Quchi, Zusanli acupoints, 1 mL per acupoint. After operation, press with cotton swab to stop bleeding. Bilateral acupoints used alternately. Once every 3 days, total 14 treatments, 6 weeks total course. Xiaofeng San acupoint application: Shenque (CV8) acupoint. Xiaofeng San composed of Danggui (Radix Angelicae Sinensis) 9 g, Shengdi (Radix Rehmanniae) 9 g, Humaren (Semen Sesami Nigrum) 9 g, Fangfeng (Radix Saposhnikoviae) 9 g, Jingjie (Herba Schizonepetae) 9 g, Chantui (Periostracum Cicadae) 9 g, Niubangzi (Fructus Arctii) 9 g, Cangzhu (Rhizoma Atractylodis) 9 g, Kushen (Radix Sophorae Flavescentis) 9 g, Mutong (Caulis Akebiae) 6 g, Shigao (Gypsum Fibrosum) 9 g, Zhimu (Rhizoma Anemarrhenae) 9 g, Gancao (Radix Glycyrrhizae) 6 g. Grind above herbs into extremely fine powder. At time of use, take small amount, mix with honey to form paste, apply to routinely disinfected Shenque acupoint. Application duration: 6 hours. Once every 3 days, total 14 treatments, 6 weeks total course. |  | Referenced from "Guiding Principles for Clinical Research of New Chinese Medicines" to calculate Symptom Score Reduce Index (SSRI). SSRI = (Pre-treatment score - Post-treatment score) / Pre-treatment score × 100%. Urticaria symptom scores before and after treatment represent pre- and post-treatment scores for chronic urticaria patients. Efficacy grades: Cured, Markedly effective, Effective, Ineffective. Cured: SSRI ≥ 90%; Markedly effective: 60% ≤ SSRI < 90%; Effective: 20% ≤ SSRI < 60%; Ineffective: SSRI < 20%. Total effective rate = (Cured + Markedly effective + Effective) / Total cases × 100%. Cure-marked effective rate = (Cured + Markedly effective) / Total cases × 100%. |
| 80 | Miaoxing Zhang | 2021 | Control group: Epinastine Hydrochloride Capsules (Chongqing Yaoyou Company, National Medicine Approval No. H20130054, batch No. 19041250) orally, 10 mg/day. 3 months per course. Observe and record clinical efficacy or recurrence before and after treatment, and at 6-month follow-up. Chinese medicine group: Self-composed Xiaofeng Zhiyang Decoction, 1 dose daily, 3 times daily, 200 mL each time. Formula: Chishao (Radix Paeoniae Rubra) 15 g, Danpi (Cortex Moutan) 10 g, Shengdi (Radix Rehmanniae) 20 g, Chantui (Periostracum Cicadae) 5 g, Fangfeng (Radix Saposhnikoviae) 10 g, Jili (Fructus Tribuli) 20 g, Jingjie (Herba Schizonepetae) 10 g, Baixianpi (Cortex Dictamni) 15 g, Kushen (Radix Sophorae Flavescentis) 10 g, Yiren (Semen Coicis) 20 g, Danzhuye (Herba Lophatheri) 10 g, Tufuling (Rhizoma Smilacis Glabrae) 20 g, Sheng Gancao (raw Radix Glycyrrhizae) 5 g. Pattern modifications: Wind exuberance with severe itching - add Wushaoshe (Zaocys) 15 g; Heat exuberance - add Huangbai (Cortex Phellodendri) 10 g, Difuzi (Fructus Kochiae) 15 g; Dampness exuberance - add Cangzhu (Rhizoma Atractylodis) 10 g, Dafupi (Pericarpium Arecae) 15 g; Qi deficiency - add Huangqi (Radix Astragali) 18 g, Guizhi (Ramulus Cinnamomi) 10 g; Blood deficiency - add Danggui (Radix Angelicae Sinensis) 10 g, Chuanxiong (Rhizoma Chuanxiong) 10 g. | Treatment group received autohemotherapy acupoint injection combined with self-composed Xiaofeng Zhiyang Decoction. Autohemotherapy acupoint injection procedure: Three-jiao acupoint selection method divided into three phases: ① Xuehai (SP10), Feishu (BL13), Quchi (LI11), Zusanli (ST36), Fengmen (BL12); ② Xuehai (SP10), Pishu (BL20), Quchi (LI11), Fenglong (ST40), Dazhu (BL11); ③ Xuehai (SP10), Shenshu (BL23), Quchi (LI11), Sanyinjiao (SP6), Geshu (BL17). Sequentially select one pair of acupoints. After routine iodophor disinfection, draw 2 mL blood from patient's cubital vein. Immediately inject 1 mL venous blood subcutaneously at each bilateral acupoint. Acupoint injection once every 5 days. 5 pairs of acupoints = one phase. After each phase of acupoint injection, 10-day interval before next phase. 3 phases = 1 course. Chinese medicine usage same as above Chinese medicine group. 3 months per course. |  | Referenced from "Industry Standard of the People's Republic of China - TCM Disease Efficacy Standards" to calculate Symptom and Sign Score Reduce Index (SSRI). SSRI = (Pre-treatment symptom and sign score - Post-treatment symptom and sign score) / Pre-treatment total symptom and sign score × 100%. SSRI < 30% = Ineffective. 30% ≤ SSRI < 70% = Effective. 70% ≤ SSRI < 95% = Markedly effective. SSRI ≥ 95% = Clinical cure. Total effective rate = (Clinical cure + Markedly effective + Effective) cases / Total cases × 100%. |
| 81 | Zhaodi Liu | 2025 | Control group received conventional western medicine treatment. Loratadine Tablets 10 mg, once daily; Cetirizine Tablets 10 mg, once every night. Both orally. Treatment for 8 weeks. | Observation group received Yinlian Qufeng Decoction oral administration combined with acupoint catgut embedding. Yinlian Qufeng Decoction: Jinyinhua (Flos Lonicerae) 15 g, Lianqiao (Fructus Forsythiae) 15 g, Tufuling (Rhizoma Smilacis Glabrae) 15 g, Sheng Shigao (raw Gypsum Fibrosum) 20 g (decocted first), Danpi (Cortex Moutan) 10 g, Yinchen (Herba Artemisiae Scopariae) 10 g, Chao Canjiang (fried Bombyx Batryticatus) 10 g, Fangfeng (Radix Saposhnikoviae) 10 g, Jingjie (Herba Schizonepetae) 10 g. 1 dose daily, add water and decoct to 400 mL, divided into 2 warm doses (morning and evening), 200 mL each. Acupoint catgut embedding: Bilateral Zusanli (ST36), bilateral Xuehai (SP10), bilateral Quchi (LI11), bilateral Feishu (BL13), bilateral Geshu (BL17). Patient in appropriate position, expose embedding sites. Strictly disinfect acupoint skin. Cut No. 0 absorbable collagen thread into 1-2 cm segments. Place at front of disposable embedding needle. Rapidly obliquely insert needle into acupoint. After lifting-thrusting deqi, push stylet to embed thread into acupoint muscle layer. Withdraw needle after treatment to prevent bleeding. Embedding once every 2 weeks. Treatment for 8 weeks. |  | Efficacy criteria: Cured: No obvious skin abnormalities, pruritus symptoms subsided, no wheal recurrence 4 weeks after stopping medication. Markedly effective: Wheal area reduced by ≥ 70% compared to pre-treatment, pruritus significantly relieved, low impact on daily life. Effective: Wheal area subsided by 30%-69%, pruritus somewhat alleviated, still some impact on daily life. Ineffective: Wheals subsided by < 30%, no improvement or worsening of pruritus symptoms. |
| 82 | Caide Yang | 2014 | Oral Claritin (Loratadine), produced by Schering-Plough Pharmaceutical Co., Ltd., Shanghai, National Medicine Approval No. H10970410. 10 mg per dose, once daily, taken at bedtime. 4 weeks per course. One month after stopping medication, follow up recurrence of clinically cured cases in both groups. | Acupoint selection: "Three Wind Points" as main points - Fengmen (BL12), Fengshi (GB31), Fengshiqian (3 cun anterior to Fengshi, coinciding with Dong's Extra Point Sima Zhong). Wind-heat invading exterior pattern - add Quchi (LI11), Xuehai (SP10), Geshu (BL17). Wind-cold binding exterior pattern - add Zusanli (ST36), Sanyinjiao (SP6), Feishu (BL13). Gastrointestinal damp-heat pattern - add Quchi (LI11), Zusanli (ST36). Qi and blood deficiency pattern - add Xuehai (SP10), Dachangshu (BL25), Zusanli (ST36), Sanyinjiao (SP6). Positioning: Determine body position, adopt different postures based on different acupoints. Point location: Under certain body position, locate and mark acupoints. Procedure: Acupoint catgut embedding - use disposable sterile embedding needle (tubular needle with inner stylet). Grasp approximately 2 cm long sterilized No. 00 catgut suture. Routine local skin disinfection. Left thumb tightens or pinches skin. Right hand holds needle, inserts to required depth. After needle sensation appears, push stylet while withdrawing needle tube, implanting catgut in muscle layer or subcutaneous tissue of acupoint. Apply adhesive bandage to needle hole. 2 treatments per course. Generally 15 days interval between treatments. Evaluate after 1 course. |  | Efficacy criteria: Cured: Rash subsided and no longer occurs, no recurrence at 6-month follow-up. Markedly effective: Rash basically subsided, occasional recurrence. Improved: Most rash subsided, recurrence frequency reduced. Ineffective: No improvement in symptoms after course. |
| 83 | CaideYang | 2014 | Drug: Loratadine, produced by Schering-Plough Pharmaceutical Co., Ltd., Shanghai, National Medicine Approval No. H10970410. Dosage and course: 10 mg per dose, once daily, taken orally at bedtime. 4 weeks per course, total 1 course. | Acupoint selection: References "Acupuncture and Moxibustion" for acupoint selection and location. "Three Wind Points" as main points - Fengmen (BL12), Fengshi (GB31), Fengshiqian (3 cun anterior to Fengshi, coinciding with Dong's Extra Point Sima Zhong). Wind-heat invading exterior pattern - add Quchi (LI11), Xuehai (SP10), Geshu (BL17). Wind-cold binding exterior pattern - add Zusanli (ST36), Sanyinjiao (SP6), Feishu (BL13). Gastrointestinal damp-heat pattern - add Quchi (LI11), Zusanli (ST36). Qi and blood deficiency pattern - add Xuehai (SP10), Geshu (BL17), Zusanli (ST36), Sanyinjiao (SP6). Positioning: Determine body position, adopt different postures based on different acupoints. Point location: Under certain body position, locate and mark according to standard acupoint selection. Procedure: Acupoint catgut embedding - use disposable sterile embedding needle (tubular needle with inner stylet). Grasp approximately 2 cm long sterilized No. 00 catgut suture, place at front of needle tip. Routine local skin disinfection. Left thumb and index finger tighten or pinch skin at insertion site. Right hand holds needle, inserts to required depth. After needle sensation appears, push stylet while withdrawing needle tube, implanting catgut in muscle layer or subcutaneous tissue of acupoint. Cover needle hole with adhesive bandage. Course: 2 treatments per course, 15 days interval, total 1 course. |  | Referenced from "Guiding Principles for Clinical Research of New Chinese Medicines" (Volume 3, formulated and issued by Ministry of Health, People's Republic of China, 1997) and literature standards. Judged based on pre- and post-treatment scores of chronic urticaria: pruritus severity, wheal size, wheal number, wheal duration, and episode frequency. |
| 84 | ZhongbingMa | 2016 | Loratadine Tablets (produced by Schering-Plough Pharmaceutical Co., Ltd., Shanghai, National Medicine Approval No. H10970410), 10 mg per dose, once daily, taken at bedtime. 4 weeks per course. | 1) Acupoint selection: "Three Wind Points" as main points - Fengmen (BL12), Fengshi (GB31), Fengshiqian (3 cun anterior to Fengshi, coinciding with Dong's Extra Point Sima Zhong). Wind-heat invading exterior pattern - add Quchi (LI11), Xuehai (SP10), Geshu (BL17). Wind-cold binding exterior pattern - add Zusanli (ST36), Sanyinjiao (SP6), Feishu (BL13). Gastrointestinal damp-heat pattern - add Quchi (LI11), Zusanli (ST36). Qi and blood deficiency pattern - add Xuehai (SP10), Geshu (BL17), Zusanli (ST36), Sanyinjiao (SP6). 2) Procedure: Select puncture needle, remove stylet. Approximately 2 cm PGLA suture, place at front of puncture needle. Suture length basically same inside and outside needle hole, making PGLA suture U-shaped. When inserting into acupoint, suture is pressed at needle tip to form double fold. After confirming suture outside needle hole enters skin and needle sensation obtained, rotate and withdraw puncture needle, embedding suture. Cover needle hole with adhesive bandage. 2 treatments per course, generally 15 days interval. |  | Referenced from "Guiding Principles for Clinical Research of New Chinese Medicines" (Volume 3, formulated and issued by Ministry of Health, People's Republic of China, 1997). Efficacy evaluated by efficacy index. Total effective rate = Cure rate + Markedly effective rate + Effective rate. |
| 85 | Gang Wang | 2013 | Control group: Oral Loratadine Tablets 10 mg per dose, once daily. Course: 2 weeks. | Treatment group received Zhao Bingnan's Jingfang Formula. Herbs: Jingjie (Herba Schizonepetae) 10 g, Fangfeng (Radix Saposhnikoviae) 10 g, Cijili (Fructus Tribuli) 15 g, Baixianpi (Cortex Dictamni) 15 g, Chantui (Periostracum Cicadae) 6 g, Fuping (Herba Spirodelae) 15 g, Bohe (Herba Menthae) 6 g, Jinyinhua (Flos Lonicerae) 15 g, Lianqiao (Fructus Forsythiae) 15 g, Danshen (Radix Salviae Miltiorrhizae) 15 g. Blood deficiency - add Danggui (Radix Angelicae Sinensis) 15 g, Chuanxiong (Rhizoma Chuanxiong) 10 g, Shengdihuang (Radix Rehmanniae) 15 g, Baishao (Radix Paeoniae Alba) 15 g. Qi deficiency - add Dangshen (Radix Codonopsis) 10 g, Sheng Huangqi (raw Radix Astragali) 15 g. Long course - add Quanxie (Scorpio) 10 g, Wushaoshe (Zaocys) 15 g. Decoction, 1 dose daily, divided into morning and evening doses, 200 mL each. Additionally, treatment group received bloodletting at bilateral Quchi (LI11). After routine disinfection, three-edged needle pricking bloodletting, 5-10 drops per acupoint. Every other day. Both groups: clinical efficacy determined after 2 weeks of treatment. |  | Efficacy criteria formulated with reference to "Diagnostic and Efficacy Standards for TCM Diseases". Cured: Rash completely subsided, no pruritus. Markedly effective: Rash basically subsided, occasional itching. Effective: Rash subsiding slowly, skin scratch marks. Ineffective: No improvement in symptoms, rash comes and goes, unbearable pruritus. |
| 86 | Zhe Wei | 2016 | Loratadine Tablets (produced by Schering-Plough Pharmaceutical Co., Ltd., Shanghai, National Medicine Approval No. H10970410), 10 mg per dose, once daily, taken at bedtime. 4 weeks per course. | 1) Acupoint selection: "Three Wind Points" as main points - Fengmen (BL12), Fengshi (GB31), Fengshiqian (3 cun anterior to Fengshi, coinciding with Dong's Extra Point Sima Zhong). Wind-heat invading exterior pattern - add Quchi (LI11), Xuehai (SP10), Geshu (BL17). Wind-cold binding exterior pattern - add Zusanli (ST36), Sanyinjiao (SP6), Feishu (BL13). Gastrointestinal damp-heat pattern - add Quchi (LI11), Zusanli (ST36). Qi and blood deficiency pattern - add Xuehai (SP10), Geshu (BL17), Zusanli (ST36), Sanyinjiao (SP6). 2) Procedure: Select puncture needle, remove stylet. Approximately 2 cm PGLA suture, place at front of puncture needle. Suture length basically same inside and outside needle hole, making PGLA suture U-shaped. When inserting into acupoint, suture is pressed at needle tip to form double fold. After confirming suture outside needle hole enters skin and needle sensation obtained, rotate and withdraw puncture needle, embedding suture. Cover needle hole with adhesive bandage. 2 treatments per course, generally 15 days interval. |  | Efficacy criteria adopted from "Guiding Principles for Clinical Research of New Chinese Medicines" (Volume 3, formulated and issued by Ministry of Health, People's Republic of China, 1997). Efficacy evaluated by efficacy index. Total effective rate = Cure rate + Markedly effective rate + Effective rate. |
| 87 | Yinshuang Hua | 2008 | Oral western medicine Loratadine 10 mg, once daily. | Treatment group. Chinese medicine: Sheng Huangqi (raw Radix Astragali) 30 g, Fangfeng (Radix Saposhnikoviae) 15 g, Chishao (Radix Paeoniae Rubra) 10 g, Honghua (Flos Carthami) 10 g, Sangzhi (Ramulus Mori) 10 g, Lianqiao (Fructus Forsythiae) 10 g, Chaihu (Radix Bupleuri) 15 g, Chenpi (Pericarpium Citri Reticulatae) 15 g, Fuping (Herba Spirodelae) 10 g, Chantui (Periostracum Cicadae) 10 g, Kushen (Radix Sophorae Flavescentis) 15 g, Baixianpi (Cortex Dictamni) 10 g. 1 dose daily, divided into morning and evening doses. Simultaneously select Xuehai (SP10), Sanyinjiao (SP6), Hegu (LI4), Qimen (LR14), Taichong (LR3). Routine disinfection. After insertion and deqi, apply even reinforcing-reducing method. Retain needles for 30 minutes. Once daily. |  | Evaluated according to "Diagnostic and Efficacy Standards for TCM Diseases". Cured: Rash subsided, no recurrence 3 months after stopping treatment. Markedly effective: Rash subsided by more than 50% or clinical symptoms alleviated, occasional recurrence after stopping treatment. Ineffective: No significant change in clinical symptoms. |
| 88 | Lianqiang Fang | 2013 | Western medicine group: Oral Cetirizine Hydrochloride 10 mg per dose, once daily. Course same as treatment group (1 month). All patients instructed to regulate emotions during treatment. Avoid fishy shrimp, crab, alcohol, coffee, and spicy irritating foods. | 1. Acupuncture group. Treatment principle, acupoint selection, procedure, course same as treatment group (only no autohemotherapy). 3.1. Acupuncture. Treatment principle: Nourish blood and dispel wind. Acupoints: Fengshi (GB31), Fengmen (BL12), Xuehai (SP10), Geshu (BL17), Ganshu (BL18), Pishu (BL20), Sanyinjiao (SP6), Zusanli (ST36), Quchi (LI11), Hegu (LI4), Waiguan (SJ5), Weizhong (BL40). All bilateral. 0.35 mm × 50 mm filiform needles, lifting-thrusting twisting-rotating manipulation. Fengshi: 1.5 cun insertion, lifting-thrusting reducing method for 2 min, retain 3-5 min. Xuehai: 1.2 cun insertion, needle tip toward sole center. After deqi, perform lifting-thrusting twisting-rotating manipulation. Needle sensation传导 downward is optimal. Retain 30 min, manipulate once every 10 min. Remaining acupoints: Needled according to respective meridian acupoint operation requirements. 30 min per session, once daily. 7 days per course, 1 day rest between courses. | Autohemotherapy. After acupuncture, patient rests for 5 min. Routine disinfection at brachial vein. Use 10 mL syringe to draw 8 mL venous blood, then rapidly inject into Sanyinjiao (SP6), Zusanli (ST36), Quchi (LI11), Xuehai (SP10). After local deqi, confirm no increased blood by aspiration, then slowly push injection. 2 mL per acupoint. Each acupoint injection follows principle of no repetition on left and right sides. Select 2 points per side. Left and right sides exchanged. Twice weekly. 7 days per course, 1 day rest between courses. | Formulated with reference to "Diagnostic and Efficacy Standards for TCM Diseases". Cured: Wheals subsided, clinical signs disappeared, no further episodes. Markedly effective: No wheals or pruritus on whole body, occasional recurrence, symptoms significantly lighter than before treatment. Improved: Wheals subsided by 30% or recurrence interval prolonged after subsiding, pruritus and other symptoms alleviated. Unimproved: No significant improvement in wheals and pruritus, or subsided by less than 30%. |
| 89 | Wei Li | 2015 | Patients received Cetirizine Hydrochloride alone, 10 mg per dose, once daily. 4 weeks course. Follow up 3 months after completion. | Yangxue Xiaofeng Decoction combined with auricular point pressing therapy. Yangxue Xiaofeng Decoction base formula: Danggui (Radix Angelicae Sinensis) 15 g, Shudi (Radix Rehmanniae Praeparata) 10 g, Chuanxiong (Rhizoma Chuanxiong) 10 g, Fangfeng (Radix Saposhnikoviae) 10 g, Baishao (Radix Paeoniae Alba) 15 g, Huangqi (Radix Astragali) 15 g, Danshen (Radix Salviae Miltiorrhizae) 15 g, Jixueteng (Caulis Spatholobi) 15 g, Baixianpi (Cortex Dictamni) 15 g, Fuling (Poria) 10 g, Gancao (Radix Glycyrrhizae) 6 g. Symptom-based modifications: Wind exuberance - add Chantui (Periostracum Cicadae) 10 g, Jiangcan (Bombyx Batryticatus) 10 g; Blood deficiency - add Ziheche (Placenta Hominis) 15 g; Yin deficiency - add Digupi (Cortex Lycii) 15 g, Shudi (Radix Rehmanniae Praeparata) 15 g; Severe pruritus - add Baijili (Fructus Tribuli) 10 g, Difuzi (Fructus Kochiae) 10 g; Insufficient sleep - add Suanzaoren (Semen Ziziphi Spinosae) 15 g; Dampness exuberance - add Tufuling (Rhizoma Smilacis Glabrae) 20 g, Kushen (Radix Sophorae Flavescentis) 15 g; Severe qi and blood deficiency - increase Huangqi and Danggui dosage. 1 dose daily, decocted in water, divided into 2 warm doses (morning and evening). Auricular point pressing method: Select Urticaria Point, Ear Center, Lung, Endocrine, Wind Stream, etc. After alcohol disinfection, apply adhesive tape with Vaccaria seeds to corresponding acupoints. Press each point firmly with fingers. Instruct patient to press 3-4 times daily until local swelling and warmth at auricular points. Change every other day. Both groups: 4 weeks treatment, 3-month follow-up. |  | Efficacy determined with reference to "Guiding Principles for Clinical Research of New Chinese Medicines". Clinical cure: Wheals disappeared, no erythema, no pruritus, no recurrence at 3-month follow-up. Markedly effective: Wheal episodes reduced by more than 70% during attacks, pruritus significantly alleviated, recurrence intervals significantly prolonged. Effective: Wheal episodes reduced by 30%-69% during attacks, pruritus somewhat alleviated, recurrence intervals somewhat prolonged. Ineffective: No significant improvement in symptoms or signs after treatment. Total effective rate = (Clinical cure + Markedly effective + Effective) / Total cases × 100%. |
| 90 | Xiaoqiong He | 2018 | Loratadine Tablets 10 mg orally, once daily. Continuous 2 weeks. | Autologous blood acupoint injection combined with oral Yupingfeng San. Autologous blood injection (use disposable 5 mL syringe to draw 4 mL cubital venous blood. Select bilateral Zusanli (ST36) acupoints, routine disinfection. Pen-style needle holding, rapid subcutaneous insertion. After slow advancement, lift-thrust to seek needle sensation. When patient feels soreness, numbness, distension, pain, confirm no blood return by aspiration, then inject venous blood into acupoint. 2 mL per side. 3 times per week, total 6 treatments) combined with Yupingfeng San (Yupingfeng Granules 5 g, twice daily, continuous 2 weeks), oral treatment. Course: 2 weeks. Follow up 3 months after treatment to calculate recurrence rate. |  | Statistics of pruritus severity, wheal number and size before and after treatment, scored by 4-grade method. 0 points: No itching, no wheals. 1 point: Mild pruritus, wheal diameter < 0.5 cm, number 1-6. 2 points: Moderate pruritus, wheal diameter 0.5-2.0 cm, number 7-12. 3 points: Severe pruritus, wheal diameter > 2.0 cm, number > 12. Efficacy evaluated by total score before and after treatment. SSRI = (Pre-treatment score - Post-treatment score) / Pre-treatment score × 100%. Cured: SSRI ≥ 90%. Markedly effective: SSRI 60%-89%. Improved: SSRI 20%-59%. Ineffective: SSRI < 20%. Effective rate calculated as cured + markedly effective. |
| 91 | Qingtang Yang | 2011 | Cetirizine 10 mg qn (every night), continuous 30 days. | Under strict sterile conditions, use 10 mL syringe to draw 4 mL of patient's own venous blood. Gently shake well. After 2-3 minutes, rapidly inject respectively into bilateral Quze (PC3) and Zusanli (ST36) acupoints. 1 mL per acupoint. Once every 3 days, 10 treatments per course, total 30 days. Other medications discontinued during treatment. |  | Four-item SSRI efficacy evaluation criteria. Basic cure: SSRI ≥ 90%. Markedly effective: 60% ≤ SSRI < 90%. Improved: 30% ≤ SSRI < 60%. Ineffective: SSRI < 30% (no change or worsening). Effective rate = sum of basic cure and markedly effective percentages. |
| 92 | Chunling Lin | 2008 | Control group 58 cases: Oral Cetirizine 10 mg per dose, once daily. Continuous 2 weeks. | Yupingfeng San (hospital preparation, components: Huangqi (Radix Astragali) 25 g, Fangfeng (Radix Saposhnikoviae), Baizhu (Rhizoma Atractylodis Macrocephalae) - 15 g each. Decocted in decoction machine, 250 mL special bag packaging. Taken before breakfast and after dinner. Continuous 2 weeks) combined with acupoint autohemotherapy. Draw 5 mL cubital venous blood. Select bilateral Zusanli acupoints, routine disinfection. Pen-style needle holding, rapid subcutaneous insertion then slow advancement. When patient feels soreness, numbness, distension, pain, immediately inject venous blood into acupoint. 2.5 mL per side. Once weekly, 2 weeks per course. Detect changes in blood IgE values (double antibody sandwich ELISA method, reagents provided by Immunology Laboratory, Institute of Basic Medical Sciences, Chinese Academy of Medical Sciences) and clinical efficacy before and after treatment. Follow up 3 months, calculate recurrence rate. |  | Statistics of pruritus severity, wheal number and size before and after treatment, scored by 4-grade method. 0 points: No itching, no wheals. 1 point: Mild pruritus, wheal diameter < 0.5 cm, number 1-6. 2 points: Moderate pruritus, wheal diameter 0.5-2.0 cm, number 7-12. 3 points: Severe pruritus, wheal diameter > 2.0 cm, number > 12. Efficacy evaluated by total score before and after treatment. SSRI = (Pre-treatment score - Post-treatment score) / Pre-treatment score × 100%. Cured: SSRI ≥ 90%. Markedly effective: SSRI 60%-89%. Improved: SSRI 20%-59%. Ineffective: SSRI < 20%. |
| 93 | Ping Chen | 2017 | Simple oral Desloratadine Citrate Tablets (Guangzhou Hairui Pharmaceutical Co., Ltd., specification: 8.8 mg × 6 tablets/box). 8.8 mg every night at bedtime. 15 days per course, total 2 courses. | First heat-sensitive treatment, then Zusanli acupoint autohemotherapy. Heat-sensitive moxibustion procedure: ① Operating environment: Treatment room kept quiet, indoor temperature controlled at 24-30°C. ② Position: Comfortable position, fully expose abdomen. ③ Exploration tool: 2.2 cm × 160 cm moxa sticks, produced by Affiliated Hospital of Jiangxi University of Traditional Chinese Medicine. ④ Exploration method: Patient lies supine on treatment bed. Ignite 2 heat-sensitive moxa sticks. Explore at Tianshu (ST25, 2 cun lateral to umbilicus, Stomach Meridian), Shenque (CV8, center of umbilicus, Conception Vessel), Qihai (CV6, anterior midline, 1.5 cun below umbilicus), Guanyuan (CV4, anterior midline, 3 cun below umbilicus). Apply moxibustion 30-50 mm from skin. Instruct patient to breathe gently, relax, focus on moxibustion points. Use warm moxibustion, circular moxibustion, meridian-traveling back-and-forth, sparrow-pecking moxibustion techniques to activate heat-sensitive acupoint meridian qi sensation. 45 min per moxibustion session, once daily. 15 days per course, total 2 courses. Autohemotherapy acupoint injection procedure: Acupoint selection: Bilateral Zusanli (ST36, 3 cun below Dubi point, 1 finger-breadth lateral to anterior crest of tibia). First disinfect selected acupoints. Then use 5 mL syringe to draw 4 mL cubital venous blood. 2 mL per acupoint injection. Withdraw needle, press with sterile cotton swab to stop bleeding. Autohemotherapy acupoint injection once every 3 days, 5 treatments per course, total 2 courses. |  | Efficacy evaluated by score comparison before and after treatment, i.e., efficacy index (SSRI). Calculation references Nimodipine method. SSRI (%) = (Pre-treatment score - Post-treatment score) / Pre-treatment score × 100%. Cured: Efficacy index = 90%. Markedly effective: 60% ≤ Efficacy index < 90%. Effective: 30% ≤ Efficacy index < 60%. Ineffective: Efficacy index < 30%. Cure-marked effective rate (%) = (Cured cases + Markedly effective cases) / Total cases in group × 100%. |

C: control group; T1: treatment group 1; T2: treatment group 2.

Supplementary Table S3 Assessment Scales for DLQI Score, Wheal Count, and Pruritus Severity

| No. | Question | Not at all | A little | A lot | Very much |
| --- | --- | --- | --- | --- | --- |
| 1 | Over the last week, how itchy, sore, painful or stinging has your skin been? | ☐ | ☐ | ☐ | ☐ |
| 2 | Over the last week, how embarrassed or self-conscious have you been because of your skin? | ☐ | ☐ | ☐ | ☐ |
| 3 | Over the last week, how much has your skin interfered with you going shopping or looking after your home or garden? | ☐ | ☐ | ☐ | ☐ |
| 4 | Over the last week, how much has your skin influenced the clothes you wear? | ☐ | ☐ | ☐ | ☐ |
| 5 | Over the last week, how much has your skin affected any social or leisure activities? | ☐ | ☐ | ☐ | ☐ |
| 6 | Over the last week, how much has your skin made it difficult for you to do any sport? | ☐ | ☐ | ☐ | ☐ |
| 7 | Over the last week, has your skin prevented you from working or studying? | ☐ No | ☐ Yes |  |  |
|  | If "No", over the last week, how much has your skin been a problem at work or studying? | ☐ | ☐ | ☐ | ☐ |
| 8 | Over the last week, how much has your skin created problems with your partner or any of your close friends or relatives? | ☐ | ☐ | ☐ | ☐ |
| 9 | Over the last week, how much has your skin caused any sexual difficulties? | ☐ | ☐ | ☐ | ☐ |
| 10 | Over the last week, how much of a problem has the treatment for your skin been, for example, by making your home messy, or by taking up time? | ☐ | ☐ | ☐ | ☐ |

Wheal Count

| Category | Clinical Manifestation | Severity | Score |
| --- | --- | --- | --- |
| Number of wheals | 0 wheals / 24 h | None | 0 |
|  | < 20 wheals / 24 h | Mild | 1 |
|  | 20–50 wheals / 24 h | Moderate | 2 |
|  | > 50 wheals / 24 h or large confluent areas | Severe | 3 |

Pruritus Severity

| Category | Clinical Manifestation | Severity | Score |
| --- | --- | --- | --- |
| Pruritus severity | No pruritus | None | 0 |
|  | Pruritus present, but not annoying | Mild | 1 |
|  | Pruritus and annoying, but does not affect normal daily activities or sleep | Moderate | 2 |
|  | Severe pruritus, significantly interfering with daily activities or sleep | Severe | 3 |

The DLQI is scored by assigning each of its 10 items a value from 0 to 3, where the response categories "not at all", "a little", "a lot", and "very much" correspond to scores of 0, 1, 2, and 3, respectively. Responses marked as "not relevant" or left unanswered are also coded as 0. The total score is calculated as the sum of all item scores, giving a possible range of 0 to 30, with higher scores reflecting a greater negative impact on the patient’s health‑related quality of life.

Supplementary Table S4 Basic Features of Included Studies

| Author | Year | Sample size | | | | Male/female | | | | Age (years) | | | | Intervention | | | | | Treatment period | Outcomes |
| --- | --- | --- | --- | --- | --- | --- | --- | --- | --- | --- | --- | --- | --- | --- | --- | --- | --- | --- | --- | --- |
|  |  | C | T1 | T2 | | C | T1 | | T2 | C | T1 | | T2 | C | T1 | | T2 | |  |  |
| YeHu | 2024 | 60 | 60 |  | | 10/11 | 25/35 | |  | 34.87±6.07 | 35.43±5.51 | |  | BLP-C | Acupuncture | |  | | 12w | ①⑥⑨ |
| YuShi | 2023 | 35 | 40 |  | | 27/33 | 15/25 | |  | 12.53±7.65 | 13.09±11.70 | |  | Antihistamine | Acupuncture+Cuppingtherapy | |  | | 4w | ①②④⑤⑥⑦ |
| YingjieWang | 2013 | 40 | 40 |  | | 11/24 | 15/25 | |  | 36.00±12.00 | 35.00±12.00 | |  | Antihistamine | Moxibustion | |  | | 2w | ① |
| JinyeGan | 2018 | 31 | 31 |  | | 16/24 | 17/14 | |  | 35.76±5.62 | 37.96±4.32 | |  | Antihistamine | BLP-C | |  | | 8d | ① |
| Yongnan Jia | 2020 | 30 | 30 | 30 | | 16/15 | 13/17 | | 14/16 | 38.1±4.87 | 37.43±5.27 | | 36.53±4.34 | Autohemotherapy+BLP-C | Autohemotherapy+Cupping therapy | | Antihistamine | | 9w | ①⑤⑧⑨ |
| Jun Zhao | 2018 | 46 | 46 |  | | 15/15 | 22/24 | |  | 35.31±13.88 | 34.75±13.60 | |  | BLP-C | Autohemotherapy+BLP-C | |  | | 8w | ①⑥⑦⑨ |
| Quan Li | 2015 | 27 | 27 |  | | 19/27 | 12/15 | |  | 17～71 | 17～69 | |  | Antihistamine | BLP-C | |  | | 4w | ①⑧⑨ |
| Hong Gao | 2009 | 30 | 30 |  | | 10/17 | 13/17 | |  | 37.50±12.90 | 35.60±13.80 | |  | Antihistamine | Acupuncture | |  | | 6w | ⑤ |
| Hong Gao | 2009 | 30 | 30 |  | | 11/19 | 13/17 | |  | 37.50±12.90 | 35.60±13.80 | |  | Antihistamine | Acupuncture | |  | | 6w | ①⑤ |
| Yuying Tang | 2015 | 60 | 60 |  | | 11/19 | 28/32 | |  | 13～63 | 15～66 | |  | Antihistamine | Acupoint injection therapy | |  | | 2w | ①⑧ |
| Zhongxun Wang | 2021 | 31 | 34 |  | | 25/35 |  | |  |  |  | |  | Antihistamine | Acupuncture | |  | | 4w | ①② |
| Qingjuan Guo | 2024 | 40 | 40 |  | |  | 21/19 | |  | 31.80±3.70 | 32.40±2.30 | |  | Antihistamine | Autohemotherapy+Moxibustion | |  | | 8w | ①③④⑤⑨ |
| Meiling Qiu | 2020 | 25 | 25 |  | | 18/22 | 14/11 | |  | 60.30±1.50 | 60.20±1.60 | |  | Antihistamine | Acupuncture+TCM | |  | | 2w | ① |
| Bingling Zhang | 2013 | 30 | 30 |  | | 13/12 | 12/18 | |  | 34.80 | 33.60 | |  | Antihistamine | Acupuncture+TCM | |  | | 4w | ①⑧⑨ |
| Ziwen Dong | 2019 | 19 | 19 |  | | 14/16 | 10/9 | |  | 34.00±1.80 | 34.30±1.50 | |  | Antihistamine | Acupuncture+TCM | |  | | 4w | ①⑧⑨ |
| Xiaoling Ye | 2023 | 40 | 40 |  | | 12/7 | 26/14 | |  | 47.12±4.84 | 48.38±3.14 | |  | Antihistamine | Moxibustion+Cupping therapy | |  | | 12w | ①②③④ |
| Yanjun Wang | 2022 | 40 | 42 |  | | 24/16 | 14/28 | |  | 35.00±7.00 | 37.00±7.00 | |  | Antihistamine | BLP-C+Auricular point sticking | |  | | 4w | ①②⑤⑥⑦⑧⑨ |
| Yanjun Wang | 2021 | 42 | 42 |  | | 10/30 | 16/26 | |  | 34.73±5.63 | 32.60±6.30 | |  | Antihistamine | Acupuncture+Cupping therapy | |  | | 4w | ①②⑤⑥⑦⑨ |
| Zhuo Huang | 2020 | 49 | 49 |  | | 14/28 | 27/22 | |  | 40.79±8.48 | 41.25±8.52 | |  | Antihistamine | Autohemotherapy+TCM | |  | | 8w | ①④ |
| Baozhen Lin | 2020 | 71 | 71 |  | | 26/23 | 34/37 | |  | 35.41±6.77 | 32.74±5.48 | |  | Antihistamine | Fire needle+TCM | |  | | 4w | ① |
| Min Li | 2014 | 30 | 30 |  | | 28/43 | 19/11 | |  | 42.71±11.42 | 42.51±10.42 | |  | Antihistamine | Acupuncture+TCM | |  | | 4w | ① |
| Libin Gu | 2019 | 78 | 78 |  | | 20/10 | 50/28 | |  | 37.14±11.37 | 37.28±11.91 | |  | Antihistamine | Acupuncture | |  | | 4w | ①④⑤⑥⑦ |
| Xinghua Lin | 2014 | 34 | 33 | 33 | | 48/30 | 12/21 | | 11/22 | 15～78 | 14～80 | | 14～78 | Antihistamine | Acupuncture+Cupping therapy | | TCM | | 8w | ①⑧⑨ |
| Hongjun Xiao | 2018 | 26 | 26 |  | | 11/23 | 16/10 | |  | 52.96±6.14 | 53.06±6.26 | |  | Antihistamine | Autohemotherapy+TCM | |  | | 4w | ①⑨ |
| Aiying Wei | 2019 | 49 | 49 |  | | 15/11 | 26/23 | |  | 42.00±3.20 | 41.50±3.10 | |  | Antihistamine | Autohemotherapy+TCM | |  | | 4w | ①⑧⑨ |
| Bianying Ren | 2020 | 25 | 25 |  | | 25/24 | 14/11 | |  | 32.35±12.33 | 32.33±12.34 | |  | Autohemotherapy | Autohemotherapy+Moxibustion | |  | | 10d | ①④⑧ |
| Xihua Kui | 2021 | 30 | 30 |  | | 15/10 | 16/14 | |  | 39.24±4.17 | 39.27±4.18 | |  | Antihistamine | Acupuncture+BLP-C | |  | | 24d | ①⑨ |
| Yan Liu | 2012 | 37 | 42 |  | | 17/13 | 18/24 | |  | 34.00±5.00 | 37.00±6.00 | |  | Antihistamine | ACE+TCM | |  | | 4w | ①⑨ |
| Juan Wang | 2019 | 66 | 68 | 66 | | 17/20 | 33/35 | | 31/35 | 49.12±6.93 | 50.13±3.95 | | 48.11±5.94 | Autohemotherapy | Autohemotherapy+Bloodletting therapy | | Bloodletting therapy | | 4w | ③④⑨ |
| Xiaoqi Liao | 2016 | 83 | 83 |  | | 32/34 |  | |  | 32.00±13.00 | 33.00±13.00 | |  | Antihistamine | Bloodletting therapy | |  | | 4w | ①⑤⑨ |
| Furong Liu | 2022 | 30 | 30 |  | |  | 11/19 | |  | 48.12±10.57 | 48.10±11.28 | |  | Antihistamine | ACE+TCM | |  | | 4w | ①④⑧⑨ |
| Xurui Wang | 2022 | 46 | 46 | 46 | |  | 20/26 | | 22/24 | 31.30±7.70 | 32.40±7.30 | | 33.1±8.10 | Antihistamine | ACE+TCM | | TCM | | 4w | ①②⑥⑦⑨ |
| Junying Jiao | 2017 | 56 | 60 | 58 | | 23/23 | 38/22 | | 37/21 | 48.10±6.54 | 47.40±7.08 | | 46.9±7.35 | Antihistamine | Moxibustion+TCM | | TCM | | 4w | ①⑧⑨ |
| Weijie Chen | 2018 | 50 | 50 |  | | 35/21 | 23/27 | |  | 50.12±3.50 | 50.03±3.33 | |  | Antihistamine | Moxibustion+TCM | |  | | 4w | ①②④⑧⑨ |
| Zhongfang Lin | 2014 | 32 | 33 |  | | 24/26 | 18/15 | |  | 27.30±6.80 | 28.60±6.40 | |  | Antihistamine | Moxibustion | |  | | 24w | ①③④⑨ |
| Qiong wu | 2017 | 30 | 30 |  | | 19/13 | 16/14 | |  | 42.90±19.10 | 43.50±13.50 | |  | Antihistamine | Autohemotherapy | |  | | 5w | ①③④ |
| Jieru He | 2009 | 30 | 30 |  | | 12/18 | 13/17 | |  | 39.90±10.60 | 35.90±9.10 | |  | Antihistamine | Acupuncture | |  | | 4w | ① |
| Wenna Shu | 2023 | 31 | 31 |  | | 16/14 | 14/17 | |  | 37.20±14.30 | 38.60±15.20 | |  | Antihistamine | Warm-needle moxibustion | |  | | 3w | ①②⑧ |
| Guihong Ye | 2018 | 28 | 28 |  | | 15/16 | 17/11 | |  | 31.20±3.10 | 30.80±2.60 | |  | Antihistamine | Acupuncture+TCM | |  | | 2w | ①⑨ |
| Hairong Wang | 2018 | 50 | 50 |  | | 15/13 | 29/21 | |  | 35.21±5.41 | 34.87±5.63 | |  | Antihistamine | Acupuncture+TCM | |  | | 无 | ① |
| Zhenqin Zhou | 2015 | 60 | 60 |  | | 27/23 | 35/25 | |  | 33.30±5.70 | 35.80±3.70 | |  | Antihistamine | Acupuncture+TCM | |  | | 1w～3w | ①⑤⑧ |
| Ning Su | 2011 | 31 | 34 |  | | 35/25 | 22/12 | |  | 14～68 | 15～69 | |  | Antihistamine | BLP-C+TCM | |  | | 2w | ①⑨ |
| Zhijun Zhang | 2024 | 53 | 53 |  | | 21/10 | 22/31 | |  | 39.42±8.16 | 40.57±8.31 | |  | Antihistamine | Acupuncture+TCM | |  | | 2w | ①③④⑧ |
| Jin Zhao | 2019 | 30 | 30 |  | | 24/29 | 19/11 | |  | 31.40±3.00 | 31.40±3.30 | |  | Antihistamine | Acupuncture+TCM | |  | | 2w | ① |
| Mengyao Yang | 2021 | 30 | 30 |  | | 17/13 | 13/17 | |  | 41.66±11.34 | 42.32±10.56 | |  | Antihistamine | Acupuncture+TCM | |  | | 4w | ①⑧⑨ |
| Yihui Zhang | 2017 | 30 | 30 |  | | 9/21 | 12/18 | |  | 31.00±0.50 | 30.00±0.60 | |  | Antihistamine | BLP-C+Autohemotherapy | |  | | 8w | ① |
| Fei Liu | 2018 | 39 | 39 |  | | 14/16 | 16/23 | |  | 39.10±14.60 | 38.60±15.30 | |  | Antihistamine | ACE+TCM | |  | | 4w | ①⑤⑧⑨ |
| Keping Tan | 2016 | 29 | 29 |  | | 15/24 |  | |  | 38.12±24.32 | 38.71±25.12 | |  | Antihistamine | ACE+TCM | |  | | 4w | ①⑤⑧⑨ |
| Yang Yang | 2020 | 50 | 50 |  | |  | 24/26 | |  | 43.30±12.20 | 48.40±10.90 | |  | Antihistamine | ACE+TCM | |  | | 8w | ①⑤⑥⑦⑧⑨ |
| Hailian Yi | 2015 | 30 | 30 |  | | 28/22 | 10/20 | |  | 38.00±4.00 | 36.00±4.00 | |  | Antihistamine | ACE+TCM | |  | | 2w | ①⑤⑧ |
| Jianfeng Dai | 2020 | 43 | 43 |  | | 8/22 | 23/20 | |  | 40.75士2.29 | 40.32±2.60 | |  | Antihistamine | ACE | |  | | 无 | ①⑧ |
| Yanjie Shang | 2019 | 30 | 30 |  | | 24/19 |  | |  |  |  | |  | Antihistamine | ACE | |  | | 4w | ① |
| Qianhong Wu | 2016 | 40 | 40 |  | |  | 22/18 | |  | 35.00±5.90 | 36.00±6.50 | |  | Antihistamine | Autohemotherapy | |  | | 4w | ①③④⑥⑦ |
| Yujia Zhen | 2019 | 20 | 20 |  | | 19/21 | 10/10 | |  | 33.50±2.70 | 35.30±2.40 | |  | Antihistamine | Autohemotherapy | |  | | 4w | ①⑨ |
| Feiying Hu | 2023 | 30 | 30 |  | | 9/11 | 18/12 | |  | 38.90±10.90 | 36.83 ±10.91 | |  | Acupuncture | Acupuncture+Moxibustion | |  | | 4w | ①②③④ |
| Qingshan Kong | 2011 | 46 | 52 |  | | 16/14 | 25/27 | |  | 16～68 | 17～70 | |  | Antihistamine | BLP-C+TCM | |  | | 4w | ① |
| Xu Kang | 2018 | 41 | 41 |  | | 21/25 | 23/18 | |  | 34.45±6.52 | 35.31±6.48 | |  | Antihistamine | Autohemotherapy+Moxibustion | |  | | 4w;6w;2w | ①⑨ |
| Minyan Wu | 2019 | 60 | 60 |  | | 22/19 | 35/25 | |  | 48.57±13.29 | 49.18±13.56 | |  | Antihistamine | Acupuncture+TCM | |  | | 4w | ①⑤⑥⑦⑨ |
| Haiyan Gu | 2020 | 45 | 45 |  | | 38/22 | 18/27 | |  | 41.79±7.49 | 40.76±8.86 | |  | Antihistamine | Acupuncture | |  | | 6w | ①⑤⑥⑦⑧⑨ |
| Yu Li | 2023 | 40 | 40 |  | | 17/28 | 16/24 | |  | 41.00±15.00 | 43.00±16.00 | |  | Antihistamine | Acupuncture+BLT+TCM | |  | | 4w | ①②③④⑤⑧ |
| Liangnan Zhang | 2019 | 45 | 45 |  | | 18/22 | 18/27 | |  | 41.79±7.49 | 40.76±8.86 | |  | Antihistamine | Acupuncture | |  | | 6w | ①⑤⑥⑦⑧⑨ |
| Wenyin Wu | 2020 | 53 | 53 |  | | 17/28 | 25/28 | |  | 42.00±6.00 | 42.00±5.00 | |  | Antihistamine | Acupuncture | |  | | 8w | ①②④⑤⑥⑦ |
| Libin Gu | 2019 | 60 | 60 |  | | 27/26 | 26/34 | |  | 41.00±12.00 | 40.00±12.00 | |  | Antihistamine | Acupuncture | |  | | 6w | ①⑤⑥⑦ |
| Zixing Liu | 2021 | 34 | 34 |  | | 28/32 | 18/16 | |  | 38.56±2.55 | 38.45±2.62 | |  | Antihistamine | Acupuncture+BLP-C | |  | | 4w | ①⑤⑥⑦ |
| Yuming Wang | 2018 | 28 | 29 |  | | 17/17 | 5/24 | |  | 43.61±12.59 | 43.50±10.66 | |  | Antihistamine | Acupuncture | |  | | 6w | ①⑨ |
| Huanmin Huo | 2014 | 80 | 80 |  | | 6/22 | 42/38 | |  | 30.00±13.00 | 32.00±12.00 | |  | Antihistamine | Acupuncture+Bloodletting therapy | |  | | 4w | ①⑧ |
| Yun Yao | 2021 | 35 | 35 |  | | 39/41 | 11/24 | |  | 32.17±4.11 | 34.17±3.97 | |  | Antihistamine | Acupuncture+Moxibustion | |  | | 3w | ①⑤⑧⑨ |
| Sikkintana | 2016 | 13 | 13 |  | | 10/25 | 5/8 | |  | 43.00±3.50 | 45.00±3.20 | |  | Antihistamine | Acupuncture+TCM | |  | | 2w | ①⑧ |
| Wuming Xia | 2017 | 61 | 61 |  | | 3/10 | 32/29 | |  | 45.50±3.20 | 46.30±4.20 | |  | Antihistamine | Acupuncture+Cupping therapy | |  | | 7-10d | ① |
| Xin Zhang | 2020 | 28 | 28 | 30 | | 35/26 | 16/12 | | 30/13 | 31.00 | 41.00 | | 37.00 | Acupuncture | Acupuncture+BLP-C+WM | | Antihistamine | | 6w | ①③④⑤⑥⑦⑧⑨ |
| Jia Xu | 2005 | 30 | 30 |  | | 28/9 | 12/18 | |  | 41.00 | 39.50 | |  | Antihistamine | Acupuncture+Autohemotherap | |  | | 4w | ① |
| Zhiling Sun | 2017 | 40 | 42 |  | | 10/20 | 18/24 | |  | 38.50±16.10 | 39.40±15.30 | |  | Antihistamine | Acupuncture+TCM | |  | | 4w | ①⑧ |
| Ying Liang | 2018 | 46 | 46 |  | | 16/24 | 26/20 | |  | 42.76±8.62 | 43.96±8.32 | |  | Antihistamine | Acupuncture+Autohemotherap | |  | | 2w | ①⑤⑨ |
| Jie Wang | 2011 | 28 | 28 |  | | 25/21 | 13/15 | |  | 14～62 | 16～59 | |  | Antihistamine | ACE+TCM | |  | | 3w | ①⑨ |
| Yuanzheng Sun | 2014 | 30 | 30 |  | | 12/16 | 13/17 | |  | 45.50±7.30 | 43.50±8.30 | |  | Antihistamine | Autohemotherapy | |  | | 3w | ① |
| Guilian Liu | 2020 | 25 | 25 |  | | 16/14 |  | |  | 36.00±0.50 | 35.00±0.80 | |  | Autohemotherapy | Autohemotherapy+Cupping therapy | |  | | 5w | ①⑤⑥⑦ |
| Jin Huang | 2016 | 25 | 25 |  | |  | 10/15 | |  | 35.30±5.70 | 30.20±6.10 | |  | Antihistamine | Autohemotherapy+TCM | |  | | 8w | ①③④⑧⑨ |
| Yunlong Cheng | 2012 | 40 | 40 |  | | 8/17 | 14/26 | |  | 26.41±12.24 | 24.51±11.03 | |  | Antihistamine | Autohemotherapy | |  | | 4w | ① |
| Bifang Chen | 2022 | 35 | 35 |  | | 13/27 | 16/19 | |  | 35.43±9.82 | 34.18±10.43 | |  | Antihistamine | Autohemotherapy+Point Application | |  | | 6w | ①⑨ |
| Miaoxing Zhang | 2021 | 65 | 65 | 65 | | 17/18 | 33/32 | | 31/34 | 37.00±8.60 | 38.30±9.20 | | 39.50±8.90 | Antihistamine | Autohemotherapy | | TCM | | 12w | ①⑨ |
| Zhaodi Liu | 2025 | 40 | 40 |  | | 35/30 | 17/23 | |  | 35.50±8.20 | 34.80±7.90 | |  | Antihistamine | ACE+TCM | |  | | 8w | ①③⑤⑥ |
| Caide Yang | 2014 | 29 | 30 |  | |  |  | |  |  |  | |  | Antihistamine | ACE | |  | | 4w | ① |
| CaideYang | 2014 | 21 | 21 |  | |  | 9/12 | |  | 43.81±15.44 | 39.26±14.78 | |  | Antihistamine | ACE | |  | | 4w | ⑤ |
| ZhongbingMa | 2016 | 30 | 30 |  | |  |  | |  | 14～65 | | | | Antihistamine | ACE | |  | | 4w | ① |
| Gang Wang | 2013 | 20 | 25 |  | | 15/30 | | | | 32.65±7.58 | | | | Antihistamine | BLP-C+TCM | |  | | 2w | ① |
| Zhe Wei | 2016 | 30 | 30 |  | |  |  | |  | 14～65 | | | | Antihistamine | ACE | |  | | 4w | ①⑦ |
| Yinshuang Hua | 2008 | 45 | 45 |  | | 25/20 | 22/23 | |  | 18～72 | | | | Antihistamine | Acupuncture+TCM | |  | | N | ① |
| Lianqiang Fang | 2013 | 20 | 21 | 21 | 21 | 9/11 | 12/9 | 10/11 | 13/8 | 30.00±8.00 | 32.00±7.00 | 32.00±7.00 | 31.00±8.00 | Antihistamine | Acupuncture | Autohemotherapy | | Acupuncture+Autohemotherap | 4w | ① |
| Wei Li | 2015 | 55 | 55 |  | |  |  | |  | 36.84±3.02 | | | | Antihistamine | TCM+Auricular point sticking | |  | | 4w | ①⑥⑨ |
| Xiaoqiong He | 2018 | 50 | 50 |  | | 42/58 | | | | 34 | | | | Antihistamine | Autohemotherapy | |  | | 2w | ①⑨ |
| Qingtang Yang | 2011 | 30 | 30 |  | | 24/36 | | | | 16～58 | | | | Antihistamine | Autohemotherapy | |  | | 4w | ①③④⑤⑥⑦⑧⑨ |
| Chunling Lin | 2008 | 58 | 68 |  | |  |  | |  | Average 34.30 years | | | | Antihistamine | Autohemotherapy+TCM | |  | | 2w | ①⑤⑨ |
| Ping Chen | 2017 | 40 | 40 |  | |  |  | |  | 18～65 | | | | Antihistamine | Autohemotherapy+Moxibustion | |  | | 4w | ⑤⑨ |

C: control group; T: treatment group; ①: Therapeutic effect; ②: DLQI (Dermatology Life Quality Index); ③: Number of wheals; ④: Itching degree; ⑤: IgE (Immunoglobulin E); ⑥: IL-4 (Interleukin-4); ⑦: IFN-γ (Interferon-gamma); ⑧: Adverse reactions; ⑨: Recurrence rate.

Supplementary Table S5 Pairwise Comparison Results of Varied Interventions on DLQI Score

| Antihistamine |  |  |  |  |  |  |  |  |  |  |  |
| --- | --- | --- | --- | --- | --- | --- | --- | --- | --- | --- | --- |
| -3.01 (-3.98, -2.03) | Acupuncture |  |  |  |  |  |  |  |  |  |  |
| -5.45 (-7.65, -3.26) | -2.44 (-4.41, -0.49) | Acupuncture+Moxibustion |  |  |  |  |  |  |  |  |  |
| -1.2 (-2.11, -0.31) | 1.8 (0.47, 3.14) | 4.24 (1.88, 6.62) | Acupuncture+Cupping therapy |  |  |  |  |  |  |  |  |
| -1.95 (-3.19, -0.71) | 1.06 (-0.52, 2.63) | 3.5 (0.98, 6.03) | -0.75 (-2.29, 0.8) | Acupuncture+Bloodletting therapy+TCM |  |  |  |  |  |  |  |
| -4.73 (-6.51, -2.94) | -1.72 (-3.76, 0.31) | 0.73 (-2.11, 3.57) | -3.52 (-5.52, -1.52) | -2.77 (-4.95, -0.6) | Acupuncture+TCM |  |  |  |  |  |  |
| 0.78 (-0.67, 2.24) | 3.79 (2.03, 5.56) | 6.23 (3.6, 8.88) | 1.99 (0.27, 3.71) | 2.73 (0.83, 4.65) | 5.51 (3.18, 7.81) | BLP-C+Auricular point sticking |  |  |  |  |  |
| -9.34 (-11.37, -7.32) | -6.34 (-8.57, -4.09) | -3.89 (-6.88, -0.91) | -8.14 (-10.35, -5.93) | -7.39 (-9.76, -5.02) | -4.62 (-7.3, -1.92) | -10.13 (-12.63, -7.63) | Moxibustion |  |  |  |  |
| -4.03 (-5.81, -2.24) | -1.02 (-3.05, 1.01) | 1.42 (-1.4, 4.25) | -2.82 (-4.82, -0.82) | -2.08 (-4.26, 0.1) | 0.7 (-1.82, 3.23) | -4.81 (-7.12, -2.5) | 5.32 (2.61, 8) | Moxibustion+TCM |  |  |  |
| -1.62 (-3.71, 0.46) | 1.38 (-0.92, 3.69) | 3.82 (0.8, 6.86) | -0.42 (-2.69, 1.86) | 0.33 (-2.11, 2.74) | 3.11 (0.35, 5.85) | -2.4 (-4.96, 0.14) | 7.72 (4.81, 10.63) | 2.41 (-0.33, 5.15) | ACE+TCM |  |  |
| -6 (-6.88, -5.13) | -2.99 (-4.31, -1.69) | -0.55 (-2.91, 1.81) | -4.8 (-6.05, -3.54) | -4.05 (-5.57, -2.53) | -1.28 (-3.26, 0.71) | -6.78 (-8.49, -5.08) | 3.34 (1.15, 5.54) | -1.98 (-3.96, 0.02) | -4.38 (-6.64, -2.12) | Warm-needle moxibustion |  |
| 0.08 (-2.15, 2.31) | 3.09 (0.67, 5.53) | 5.53 (2.4, 8.68) | 1.28 (-1.12, 3.69) | 2.04 (-0.53, 4.57) | 4.81 (1.94, 7.65) | -0.7 (-3.36, 1.97) | 9.43 (6.41, 12.44) | 4.11 (1.25, 6.97) | 1.71 (-0.24, 3.64) | 6.08 (3.69, 8.47) | TCM |

Supplementary Table S6 Pairwise Comparison Results of Varied Interventions on Wheal Count

| Antihistamine |  |  |  |  |  |  |  |  |  |  |
| --- | --- | --- | --- | --- | --- | --- | --- | --- | --- | --- |
| -0.9 (-1.85, 0.06) | Acupuncture |  |  |  |  |  |  |  |  |  |
| 0 (-0.98, 0.99) | 0.9 (-0.47, 2.28) | Acupuncture+BLP-C |  |  |  |  |  |  |  |  |
| -0.48 (-1.46, 0.5) | 0.42 (-0.95, 1.79) | -0.49 (-1.89, 0.91) | Acupuncture+BLP-C+Antihistamine |  |  |  |  |  |  |  |
| -0.22 (-1.15, 0.71) | 0.68 (-0.65, 2.01) | -0.22 (-1.58, 1.13) | 0.26 (-1.1, 1.61) | Acupuncture+TCM |  |  |  |  |  |  |
| -0.06 (-0.61, 0.47) | 0.84 (-0.28, 1.92) | -0.07 (-1.2, 1.05) | 0.42 (-0.71, 1.52) | 0.16 (-0.92, 1.22) | Autohemotherapy |  |  |  |  |  |
| -0.53 (-1.45, 0.41) | 0.37 (-0.96, 1.71) | -0.53 (-1.89, 0.82) | -0.05 (-1.4, 1.31) | -0.31 (-1.61, 1) | -0.47 (-1.52, 0.62) | Autohemotherapy+TCM |  |  |  |  |
| -0.04 (-1.12, 1.01) | 0.85 (-0.59, 2.28) | -0.04 (-1.51, 1.39) | 0.44 (-1.03, 1.86) | 0.18 (-1.24, 1.58) | 0.02 (-0.9, 0.94) | 0.49 (-0.93, 1.87) | Bloodletting therapy |  |  |  |
| -0.73 (-2.15, 0.66) | 0.17 (-1.54, 1.86) | -0.73 (-2.48, 0.97) | -0.25 (-1.98, 1.44) | -0.51 (-2.2, 1.16) | -0.67 (-1.97, 0.63) | -0.2 (-1.89, 1.46) | -0.69 (-1.61, 0.23) | Autohemotherapy+Bloodletting therapy |  |  |
| -0.42 (-1.09, 0.26) | 0.48 (-0.69, 1.65) | -0.42 (-1.62, 0.78) | 0.07 (-1.13, 1.26) | -0.2 (-1.35, 0.95) | -0.35 (-1.21, 0.52) | 0.11 (-1.04, 1.26) | -0.37 (-1.62, 0.9) | 0.31 (-1.23, 1.89) | Moxibustion |  |
| -0.86 (-1.84, 0.12) | 0.04 (-1.34, 1.4) | -0.86 (-2.25, 0.53) | -0.38 (-1.77, 1.01) | -0.64 (-1.99, 0.7) | -0.8 (-1.91, 0.33) | -0.33 (-1.68, 1.02) | -0.82 (-2.26, 0.64) | -0.13 (-1.84, 1.6) | -0.44 (-1.63, 0.74) | ACE+TCM |

Supplementary Table S7 Pairwise Comparison Results of Varied Interventions on the Severity of Pruritus

| Antihistamine |  |  |  |  |  |  |  |  |  |  |  |  |
| --- | --- | --- | --- | --- | --- | --- | --- | --- | --- | --- | --- | --- |
| -0.99 (-1.29, -0.69) | Acupuncture |  |  |  |  |  |  |  |  |  |  |  |
| -0.29 (-0.69, 0.11) | 0.7 (0.2, 1.2) | Acupuncture+BLP-C |  |  |  |  |  |  |  |  |  |  |
| -0.37 (-0.73, -0.02) | 0.62 (0.15, 1.08) | -0.09 (-0.62, 0.45) | Acupuncture+BLP-C+Antihistamine |  |  |  |  |  |  |  |  |  |
| -0.29 (-0.47, -0.11) | 0.7 (0.35, 1.05) | 0 (-0.44, 0.44) | 0.09 (-0.31, 0.48) | Acupuncture+TCM |  |  |  |  |  |  |  |  |
| 0.05 (0, 0.1) | 1.04 (0.73, 1.34) | 0.34 (-0.07, 0.74) | 0.42 (0.07, 0.78) | 0.34 (0.15, 0.52) | Autohemotherapy |  |  |  |  |  |  |  |
| -0.24 (-0.5, 0.02) | 0.75 (0.35, 1.15) | 0.05 (-0.43, 0.53) | 0.14 (-0.3, 0.57) | 0.05 (-0.26, 0.36) | -0.29 (-0.55, -0.03) | Autohemotherapy+TCM |  |  |  |  |  |  |
| -1.03 (-1.15, -0.91) | -0.04 (-0.36, 0.28) | -0.74 (-1.16, -0.32) | -0.66 (-1.03, -0.28) | -0.74 (-0.96, -0.53) | -1.08 (-1.2, -0.96) | -0.79 (-1.08, -0.51) | Autohemotherapy+Moxibustion |  |  |  |  |  |
| 0.05 (-0.04, 0.14) | 1.04 (0.72, 1.35) | 0.34 (-0.08, 0.75) | 0.42 (0.06, 0.79) | 0.34 (0.14, 0.54) | 0 (-0.08, 0.08) | 0.29 (0.01, 0.56) | 1.08 (0.93, 1.22) | Bloodletting therapy |  |  |  |  |
| -0.87 (-0.98, -0.76) | 0.12 (-0.2, 0.44) | -0.58 (-1, -0.17) | -0.5 (-0.87, -0.13) | -0.58 (-0.79, -0.37) | -0.92 (-1.02, -0.82) | -0.63 (-0.91, -0.35) | 0.16 (0, 0.32) | -0.92 (-0.98, -0.86) | Autohemotherapy+Bloodletting therapy |  |  |  |
| -0.39 (-0.64, -0.15) | 0.6 (0.21, 0.99) | -0.11 (-0.58, 0.37) | -0.02 (-0.45, 0.41) | -0.1 (-0.41, 0.2) | -0.44 (-0.69, -0.19) | -0.15 (-0.51, 0.2) | 0.64 (0.36, 0.91) | -0.44 (-0.7, -0.18) | 0.48 (0.21, 0.75) | Moxibustion |  |  |
| -0.8 (-0.98, -0.62) | 0.19 (-0.16, 0.54) | -0.51 (-0.95, -0.08) | -0.42 (-0.82, -0.03) | -0.51 (-0.76, -0.26) | -0.85 (-1.03, -0.67) | -0.56 (-0.87, -0.25) | 0.23 (0.02, 0.44) | -0.85 (-1.05, -0.65) | 0.07 (-0.14, 0.28) | -0.41 (-0.71, -0.1) | Moxibustion+TCM |  |
| -0.16 (-0.46, 0.14) | 0.83 (0.4, 1.26) | 0.13 (-0.38, 0.63) | 0.21 (-0.25, 0.68) | 0.13 (-0.22, 0.48) | -0.21 (-0.52, 0.1) | 0.08 (-0.32, 0.48) | 0.87 (0.54, 1.19) | -0.21 (-0.53, 0.11) | 0.71 (0.39, 1.03) | 0.23 (-0.16, 0.62) | 0.64 (0.29, 0.99) | ACE+TCM |

Supplementary Table S8 Pairwise Comparison Results of Varied Interventions on IL-4 Concentration

| Antihistamine |  |  |  |  |  |  |  |  |  |  |  |  |
| --- | --- | --- | --- | --- | --- | --- | --- | --- | --- | --- | --- | --- |
| -5.24 (-62.44, 51.64) | Acupuncture |  |  |  |  |  |  |  |  |  |  |  |
| 2.66 (-89.81, 95.2) | 7.91 (-100.37, 117.22) | Acupuncture+Cupping therapy |  |  |  |  |  |  |  |  |  |  |
| -115.97 (-210, -24.51) | -110.82 (-220.63, -3.18) | -118.66 (-251.02, 11.02) | Acupuncture+BLP-C |  |  |  |  |  |  |  |  |  |
| -194.39 (-317.68, -71.1) | -189.02 (-324.7, -53.62) | -197.15 (-351.46, -43.36) | -78.31 (-201.42, 46.1) | Acupuncture+BLP-C+western medicine |  |  |  |  |  |  |  |  |
| -11.37 (-142.38, 119) | -6.04 (-149.44, 135.52) | -14.02 (-175.04, 145.18) | 104.31 (-54.39, 265.86) | 182.95 (3.39, 362.37) | Acupuncture+TCM |  |  |  |  |  |  |  |
| -6.19 (-92.15, 79.85) | -0.78 (-100.28, 97.67) | -8.65 (-135.74, 117.32) | 109.91 (-15.74, 237.61) | 188.31 (38.24, 338.74) | 5.36 (-150.96, 162.39) | Autohemotherapy |  |  |  |  |  |  |
| -12.9 (-136.7, 110.02) | -7.64 (-133.19, 117.13) | -15.72 (-171.22, 138.06) | 103.1 (-50.78, 257.64) | 181.37 (7.59, 355.92) | -1.54 (-180.75, 178.66) | -6.81 (-117.77, 104.67) | Autohemotherapy+BLP-C |  |  |  |  |  |
| -4.51 (-121.97, 112.83) | 0.66 (-110.14, 112.18) | -7.14 (-157.33, 142.49) | 111.5 (-36.79, 261.9) | 189.93 (19.05, 359.71) | 6.72 (-168.02, 183.65) | 1.43 (-123, 126.9) | 8.39 (-103, 120.13) | BLP-C |  |  |  |  |
| -0.32 (-130.95, 130.29) | 5.05 (-137.67, 147.15) | -3.36 (-163.33, 156.83) | 115.6 (-43.43, 276.67) | 194.03 (14.29, 373.79) | 10.9 (-173.65, 196.36) | 5.61 (-150.88, 163) | 12.41 (-167.67, 192.77) | 4.35 (-171.42, 180.44) | BLP-C+Auricular point sticking |  |  |  |
| -12.26 (-87.4, 63.64) | -6.93 (-101.06, 87.85) | -14.92 (-134.65, 104.76) | 103.88 (-14.11, 224.4) | 182.19 (37.73, 327.24) | -0.73 (-151.89, 151.15) | -6.21 (-120.17, 109.21) | 0.92 (-143.19, 145.97) | -7.46 (-147.09, 132.9) | -11.78 (-162.2, 139.78) | ACE+TCM |  |  |
| -6.32 (-125.31, 112.74) | -1.04 (-133.36, 130.39) | -9.07 (-160.18, 142.23) | 109.6 (-40.55, 262.23) | 187.94 (17.16, 359.48) | 5.31 (-171.48, 181.93) | -0.35 (-146.14, 146.7) | 6.81 (-164.36, 177.47) | -1.85 (-169, 165.87) | -5.91 (-182.33, 171.72) | 5.91 (-113.2, 124.99) | TCM |  |
| -0.57 (-131.01, 130.22) | 4.73 (-137.47, 147.05) | -3.24 (-163.08, 156.28) | 115.76 (-43.48, 276.44) | 193.79 (14.3, 373.82) | 10.93 (-173.21, 195.98) | 5.64 (-150.99, 161.36) | 12.64 (-166.83, 191.27) | 4.24 (-171.61, 179.9) | 0.15 (-185.5, 185.1) | 11.63 (-139.72, 162.6) | 5.75 (-171.26, 182.3) | TCM+Auricular point sticking |

Supplementary Table S9 Pairwise Comparison Results of Varied Interventions on IFN-γ Concentration

| Antihistamine |  |  |  |  |  |  |  |  |  |  |
| --- | --- | --- | --- | --- | --- | --- | --- | --- | --- | --- |
| 5.49 (0.38, 10.61) | Acupuncture |  |  |  |  |  |  |  |  |  |
| -6.09 (-14.95, 0.4) | -11.54 (-22.1, -3.61) | Acupuncture+Cupping therapy |  |  |  |  |  |  |  |  |
| -10 (-22.09, 2.06) | -15.49 (-28.56, -2.39) | -3.95 (-16.95, 11.62) | Acupuncture+BLP-C |  |  |  |  |  |  |  |
| 32.36 (19, 46.49) | 26.88 (12.69, 41.87) | 38.58 (24.83, 54.65) | 42.34 (24.57, 61.1) | Acupuncture+BLP-C+WM |  |  |  |  |  |  |
| 1.44 (-10.07, 12.91) | -4.06 (-16.67, 8.5) | 7.46 (-4.94, 22.54) | 11.46 (-5.15, 28.02) | -30.89 (-49.33, -13.74) | Acupuncture+TCM |  |  |  |  |  |
| 5.18 (-3.8, 13.94) | -0.3 (-10.68, 9.79) | 11.29 (0.65, 24.04) | 15.18 (0.02, 30.09) | -27.18 (-44.06, -11.44) | 3.76 (-10.9, 18.12) | Autohemotherapy |  |  |  |  |
| 8.36 (-6.21, 22.7) | 2.85 (-12.63, 18.07) | 14.42 (-0.82, 31.87) | 18.36 (-0.62, 37.08) | -23.98 (-44.46, -4.78) | 6.93 (-11.68, 25.3) | 3.17 (-8.31, 14.6) | Autohemotherapy+Cupping therapy |  |  |  |
| -0.09 (-11.57, 11.36) | -5.6 (-18.11, 6.98) | 5.93 (-6.51, 21.06) | 9.91 (-6.82, 26.54) | -32.43 (-50.74, -15.21) | -1.53 (-17.71, 14.64) | -5.3 (-19.58, 9.34) | -8.45 (-26.75, 10.17) | BLP-C+Auricular point sticking |  |  |
| 8.17 (0.08, 16.57) | 2.68 (-6.86, 12.63) | 14.23 (4.4, 27.02) | 18.16 (3.76, 32.95) | -24.15 (-40.46, -8.5) | 6.74 (-7.25, 21.01) | 3 (-8.83, 15.3) | -0.18 (-16.54, 16.8) | 8.28 (-5.68, 22.48) | ACE+TCM |  |
| 3.93 (-7.11, 15.14) | -1.55 (-13.73, 10.78) | 9.99 (-2.16, 24.95) | 13.95 (-2.43, 30.4) | -28.39 (-46.36, -11.24) | 2.51 (-13.36, 18.45) | -1.24 (-15.28, 13.18) | -4.4 (-22.38, 14.07) | 4.04 (-11.94, 20.09) | -4.25 (-15.44, 6.77) | TCM |

Supplementary Table S10 Pairwise Comparison Results of Varied Interventions on Relapse Rate

| Antihistamine |  |  |  |  |  |  |  |  |  |  |  |  |  |  |  |  |  |  |  |  |  |  |  |  |  |
| --- | --- | --- | --- | --- | --- | --- | --- | --- | --- | --- | --- | --- | --- | --- | --- | --- | --- | --- | --- | --- | --- | --- | --- | --- | --- |
| 0.92 (0.37, 2.27) | Antihistamine+Other western medicines |  |  |  |  |  |  |  |  |  |  |  |  |  |  |  |  |  |  |  |  |  |  |  |  |
| 0.14 (0.06, 0.28) | 0.15 (0.05, 0.46) | Acupuncture |  |  |  |  |  |  |  |  |  |  |  |  |  |  |  |  |  |  |  |  |  |  |  |
| 0.26 (0.06, 0.9) | 0.28 (0.05, 1.3) | 1.89 (0.39, 8.27) | Acupuncture+Moxibustion |  |  |  |  |  |  |  |  |  |  |  |  |  |  |  |  |  |  |  |  |  |  |
| 0.09 (0.03, 0.29) | 0.1 (0.02, 0.42) | 0.69 (0.15, 2.79) | 0.37 (0.06, 2.29) | Acupuncture+Cupping therapy |  |  |  |  |  |  |  |  |  |  |  |  |  |  |  |  |  |  |  |  |  |
| 0.31 (0.09, 0.94) | 0.34 (0.07, 1.42) | 2.24 (0.54, 8.88) | 1.2 (0.2, 7.3) | 3.27 (0.6, 18.33) | Acupuncture+BLP-C |  |  |  |  |  |  |  |  |  |  |  |  |  |  |  |  |  |  |  |  |
| 0.16 (0.02, 0.71) | 0.17 (0.02, 1.01) | 1.16 (0.13, 6.3) | 0.62 (0.06, 4.87) | 1.69 (0.16, 12.37) | 0.51 (0.05, 3.61) | Acupuncture+Bloodletting therapy |  |  |  |  |  |  |  |  |  |  |  |  |  |  |  |  |  |  |  |
| 0.03 (0, 0.28) | 0.04 (0, 0.37) | 0.25 (0.01, 2.41) | 0.13 (0, 1.74) | 0.36 (0.01, 4.33) | 0.11 (0, 0.96) | 0.21 (0.01, 4.18) | Acupuncture+BLP-C+western medicine |  |  |  |  |  |  |  |  |  |  |  |  |  |  |  |  |  |  |
| 0.26 (0.12, 0.5) | 0.28 (0.09, 0.84) | 1.87 (0.67, 5.18) | 0.98 (0.23, 4.8) | 2.67 (0.69, 11.89) | 0.82 (0.22, 3.34) | 1.59 (0.3, 13.88) | 7.52 (0.78, 244.89) | Acupuncture+TCM |  |  |  |  |  |  |  |  |  |  |  |  |  |  |  |  |  |
| 0.16 (0.09, 0.3) | 0.18 (0.06, 0.52) | 1.2 (0.58, 2.54) | 0.63 (0.16, 2.9) | 1.73 (0.47, 7.25) | 0.53 (0.15, 2.1) | 1.03 (0.2, 8.9) | 4.8 (0.51, 161.55) | 0.64 (0.25, 1.64) | Autohemotherapy |  |  |  |  |  |  |  |  |  |  |  |  |  |  |  |  |
| 0.26 (0.03, 1.31) | 0.28 (0.03, 1.81) | 1.87 (0.2, 11.51) | 0.97 (0.09, 8.71) | 2.69 (0.25, 22) | 0.82 (0.08, 6.44) | 1.61 (0.12, 21.6) | 7.46 (0.37, 330.32) | 1 (0.11, 5.97) | 1.55 (0.17, 8.97) | Autohemotherapy+Cupping therapy |  |  |  |  |  |  |  |  |  |  |  |  |  |  |  |
| 0.15 (0.01, 2.34) | 0.17 (0.01, 2.96) | 1.13 (0.06, 19.32) | 0.61 (0.03, 12.74) | 1.65 (0.07, 33.51) | 0.51 (0.02, 9.95) | 0.99 (0.03, 28.85) | 4.86 (0.12, 343.7) | 0.62 (0.03, 10.26) | 0.94 (0.05, 15.43) | 0.63 (0.02, 18.89) | Autohemotherapy+BLP-C |  |  |  |  |  |  |  |  |  |  |  |  |  |  |
| 0.27 (0.13, 0.52) | 0.29 (0.12, 0.69) | 1.95 (0.71, 5.44) | 1.02 (0.24, 5.08) | 2.8 (0.73, 12.34) | 0.86 (0.23, 3.51) | 1.67 (0.32, 14.38) | 7.84 (0.83, 253.48) | 1.04 (0.38, 2.82) | 1.62 (0.64, 4.12) | 1.05 (0.18, 9.65) | 1.71 (0.1, 35.44) | Autohemotherapy+TCM |  |  |  |  |  |  |  |  |  |  |  |  |  |
| 0.1 (0.04, 0.25) | 0.11 (0.03, 0.39) | 0.74 (0.21, 2.44) | 0.39 (0.08, 2.13) | 1.05 (0.23, 5.25) | 0.33 (0.07, 1.52) | 0.63 (0.1, 6.09) | 2.98 (0.28, 104.87) | 0.39 (0.12, 1.26) | 0.61 (0.19, 1.87) | 0.4 (0.06, 3.9) | 0.65 (0.04, 13.36) | 0.38 (0.11, 1.19) | Autohemotherapy+Moxibustion |  |  |  |  |  |  |  |  |  |  |  |  |
| 0.38 (0.14, 0.95) | 0.41 (0.11, 1.48) | 2.79 (0.84, 9.21) | 1.46 (0.3, 8) | 4.01 (0.89, 20.14) | 1.23 (0.28, 5.62) | 2.36 (0.39, 22.5) | 11.34 (1.1, 383.33) | 1.49 (0.46, 4.76) | 2.31 (0.73, 7.1) | 1.5 (0.22, 14.33) | 2.44 (0.13, 51.58) | 1.42 (0.43, 4.55) | 3.78 (1.01, 14.73) | Bloodletting therapy |  |  |  |  |  |  |  |  |  |  |  |
| 0.62 (0.05, 7.14) | 0.68 (0.04, 8.92) | 4.53 (0.33, 58.24) | 2.45 (0.13, 40.27) | 6.59 (0.4, 101.59) | 2.03 (0.12, 31.08) | 3.99 (0.2, 89.38) | 19.37 (0.62, 1177.01) | 2.46 (0.17, 31.56) | 3.77 (0.27, 46.81) | 2.53 (0.11, 59.74) | 3.97 (1.19, 15.72) | 2.35 (0.16, 29.69) | 6.16 (0.39, 86.18) | 1.65 (0.1, 23.13) | BLP-C |  |  |  |  |  |  |  |  |  |  |
| 0.18 (0.04, 0.68) | 0.19 (0.03, 0.96) | 1.29 (0.25, 6.03) | 0.68 (0.1, 4.86) | 1.86 (0.29, 11.95) | 0.58 (0.09, 3.48) | 1.13 (0.13, 12.68) | 5.26 (0.39, 205.67) | 0.7 (0.13, 3.23) | 1.07 (0.21, 4.87) | 0.7 (0.08, 8.33) | 1.13 (0.05, 28.39) | 0.66 (0.13, 3.06) | 1.75 (0.31, 9.51) | 0.46 (0.08, 2.43) | 0.28 (0.02, 5.26) | BLP-C+Auricular point sticking |  |  |  |  |  |  |  |  |  |
| 0.42 (0.08, 1.83) | 0.45 (0.07, 2.66) | 3.06 (0.47, 16.4) | 1.63 (0.2, 12.75) | 4.4 (0.6, 32.06) | 1.35 (0.17, 9.44) | 2.67 (0.27, 32.32) | 12.26 (0.78, 485.84) | 1.64 (0.26, 8.65) | 2.56 (0.41, 12.75) | 1.63 (0.23, 15.4) | 2.67 (0.11, 69.72) | 1.57 (0.25, 8.05) | 4.13 (0.6, 25.16) | 1.09 (0.16, 6.63) | 0.67 (0.04, 13.17) | 2.35 (0.27, 19.08) | BLP-C+Autohemotherapy |  |  |  |  |  |  |  |  |
| 0.04 (0, 1.11) | 0.05 (0, 1.44) | 0.32 (0, 9.12) | 0.17 (0, 6.1) | 0.46 (0, 15.23) | 0.14 (0, 4.78) | 0.28 (0, 12.85) | 1.33 (0.01, 145.97) | 0.17 (0, 4.83) | 0.26 (0, 7.34) | 0.17 (0, 8.63) | 0.27 (0, 22.49) | 0.16 (0, 4.72) | 0.43 (0, 13.09) | 0.11 (0, 3.44) | 0.07 (0, 4.51) | 0.25 (0, 9.08) | 0.1 (0, 4.21) | BLP-C+TCM |  |  |  |  |  |  |  |
| 0.08 (0.01, 0.46) | 0.08 (0.01, 0.63) | 0.56 (0.06, 4.04) | 0.29 (0.03, 2.96) | 0.81 (0.08, 7.53) | 0.25 (0.02, 2.24) | 0.48 (0.04, 7.16) | 2.31 (0.11, 102.24) | 0.3 (0.03, 2.14) | 0.47 (0.05, 3.21) | 0.3 (0.02, 4.85) | 0.49 (0.02, 14.61) | 0.29 (0.03, 2.02) | 0.76 (0.08, 6.02) | 0.2 (0.02, 1.57) | 0.12 (0, 2.75) | 0.43 (0.03, 4.7) | 0.18 (0.01, 2.23) | 1.77 (0.04, 244.27) | Moxibustion |  |  |  |  |  |  |
| 0.1 (0.02, 0.36) | 0.11 (0.02, 0.38) | 0.75 (0.13, 3.24) | 0.39 (0.05, 2.6) | 1.08 (0.16, 6.55) | 0.33 (0.05, 1.92) | 0.64 (0.07, 7.12) | 2.98 (0.21, 110.2) | 0.4 (0.07, 1.73) | 0.62 (0.11, 2.55) | 0.4 (0.04, 4.64) | 0.64 (0.03, 15.16) | 0.39 (0.07, 1.47) | 1.02 (0.16, 5.06) | 0.27 (0.04, 1.32) | 0.16 (0.01, 2.81) | 0.57 (0.07, 4.07) | 0.24 (0.03, 2.09) | 2.3 (0.06, 265.29) | 1.32 (0.12, 15.71) | Moxibustion+TCM |  |  |  |  |  |
| 0.17 (0.1, 0.29) | 0.19 (0.06, 0.51) | 1.25 (0.5, 3.17) | 0.66 (0.17, 3) | 1.79 (0.52, 7.33) | 0.55 (0.16, 2.13) | 1.07 (0.22, 8.8) | 5.04 (0.56, 163.89) | 0.67 (0.28, 1.64) | 1.04 (0.46, 2.36) | 0.67 (0.12, 5.94) | 1.11 (0.07, 21.45) | 0.65 (0.27, 1.54) | 1.7 (0.59, 5.27) | 0.45 (0.16, 1.35) | 0.28 (0.02, 3.75) | 0.97 (0.22, 4.7) | 0.41 (0.08, 2.48) | 3.94 (0.15, 383.11) | 2.24 (0.34, 19.44) | 1.66 (0.43, 8.75) | ACE+TCM |  |  |  |  |
| 0.34 (0.19, 0.62) | 0.38 (0.15, 0.88) | 2.53 (0.99, 6.51) | 1.33 (0.33, 6.19) | 3.6 (1.05, 14.78) | 1.12 (0.31, 4.4) | 2.16 (0.43, 18.1) | 10.04 (1.1, 320.45) | 1.34 (0.54, 3.4) | 2.1 (0.92, 4.9) | 1.36 (0.23, 11.85) | 2.24 (0.13, 44.15) | 1.29 (0.56, 3.03) | 3.42 (1.14, 11.09) | 0.91 (0.3, 2.83) | 0.55 (0.04, 7.58) | 1.94 (0.45, 9.74) | 0.82 (0.16, 5.01) | 8 (0.28, 760.22) | 4.53 (0.66, 40.1) | 3.35 (0.95, 16.5) | 2.01 (0.95, 4.38) | TCM |  |  |  |
| 0.16 (0.04, 0.49) | 0.17 (0.04, 0.74) | 1.19 (0.26, 4.58) | 0.63 (0.1, 3.91) | 1.71 (0.3, 9.44) | 0.52 (0.09, 2.78) | 1.01 (0.13, 10.66) | 4.75 (0.39, 175.02) | 0.63 (0.14, 2.37) | 0.99 (0.23, 3.56) | 0.64 (0.08, 6.7) | 1.04 (0.05, 22.81) | 0.6 (0.14, 2.3) | 1.6 (0.32, 7.27) | 0.43 (0.08, 1.84) | 0.26 (0.02, 4.25) | 0.92 (0.14, 5.84) | 0.38 (0.05, 3.01) | 3.75 (0.11, 390.96) | 2.1 (0.22, 22.6) | 1.58 (0.26, 10.86) | 0.95 (0.23, 3.26) | 0.47 (0.11, 1.67) | TCM+Auricular point sticking |  |  |
| 0.02 (0, 0.09) | 0.02 (0, 0.13) | 0.15 (0.02, 0.61) | 0.08 (0.01, 0.62) | 0.21 (0.02, 1.53) | 0.07 (0.01, 0.44) | 0.13 (0.01, 1.59) | 0.59 (0.03, 24.55) | 0.08 (0.01, 0.42) | 0.13 (0.02, 0.51) | 0.08 (0.01, 1.07) | 0.13 (0, 3.43) | 0.08 (0.01, 0.4) | 0.2 (0.02, 1.24) | 0.05 (0.01, 0.32) | 0.03 (0, 0.64) | 0.11 (0.01, 0.95) | 0.05 (0, 0.47) | 0.46 (0.01, 58.06) | 0.27 (0.02, 3.43) | 0.2 (0.02, 1.73) | 0.12 (0.01, 0.57) | 0.06 (0.01, 0.29) | 0.13 (0.01, 0.92) | Acupuncture+Autohemotherap |  |
| 0.16 (0.03, 0.64) | 0.17 (0.03, 0.89) | 1.15 (0.2, 5.73) | 0.61 (0.08, 4.45) | 1.65 (0.23, 11.3) | 0.51 (0.07, 3.24) | 0.99 (0.11, 11.34) | 4.63 (0.33, 179.72) | 0.61 (0.11, 2.97) | 0.95 (0.18, 4.47) | 0.62 (0.06, 7.85) | 1.02 (0.04, 24.21) | 0.59 (0.11, 2.85) | 1.56 (0.25, 8.7) | 0.41 (0.07, 2.29) | 0.25 (0.01, 4.65) | 0.88 (0.11, 6.76) | 0.38 (0.04, 3.42) | 3.61 (0.1, 409.96) | 2.04 (0.18, 25.37) | 1.54 (0.2, 12.56) | 0.92 (0.17, 4.11) | 0.46 (0.08, 2.1) | 0.97 (0.14, 6.63) | 7.78 (0.89, 90.18) | Autohemotherapy+Point Application |
